# Catalytic enantioselective bromohydroxylation of cinnamyl alcohols†

**DOI:** 10.1039/d1ra02297k

**Published:** 2021-04-07

**Authors:** Jing Li, Yian Shi

**Affiliations:** Institute of Natural and Synthetic Organic Chemistry, Changzhou University Changzhou 213164 P. R. China shiyian@cczu.edu.cn; Department of Chemistry, Colorado State University Fort Collins Colorado 80523 USA

## Abstract

This work describes an effective enantioselective bromohydroxylation of cinnamyl alcohols with (DHQD)_2_PHAL as the catalyst and H_2_O as the nucleophile, providing a variety of corresponding optically active bromohydrins with up to 95% ee.

## Abstract 2

Optically active bromohydrins are obtained with up to 95% ee *via* asymmetric bromohydroxylation of cinnamyl alcohols with H_2_O as nucleophile.

## Graphical abstract

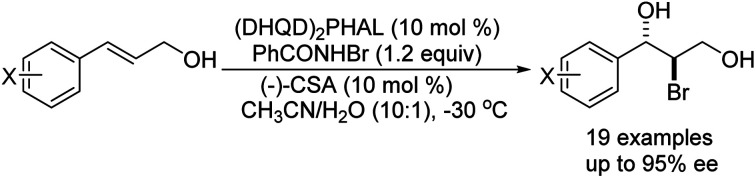

Electrophilic halogenation of olefins allows installation of two stereogenic centers onto the C–C double bond and is one of the most important transformations in organic chemistry.^1^ Optically active halogen containing products resulting from asymmetric halogenation would serve as versatile chiral building blocks for organic synthesis. As a result, extensive efforts have been devoted to the development of asymmetric halogenation process. In recent years, great progress has been made in both intramolecular^2,3^ and intermolecular^4,5^ reaction processes with various types of olefins and nucleophiles. However, there are still challenges remaining to be addressed. In many cases, the developed catalytic systems often only apply to certain ranges of substrates and the reaction reactivity as well as selectivity can't be rationally adjusted. The substrate scope is also often difficult to be logically extended and requires much experimentation, largely due to the complexity of the reaction systems and the lack of clear understanding of the reaction mechanisms.

Halohydroxylation of olefins simply with H_2_O as nucleophile is a classic electrophilic addition reaction in organic chemistry and produces synthetically useful halohydrins (Scheme 1). Asymmetric version of such process has been challenging with only a few reports.^6,7^ As part of our general intertest in asymmetric halogenation,^8^ recently we have been investigating the intermolecular asymmetric reaction processes, particularly with unfunctionalized olefins, which has been a long standing challenging problem. During such studies, we have found that up to 92% ee could be achieved for the bromoesterification of unfunctionalized olefins with (DHQD)_2_PHAL (Scheme 2, eqn (a)).^9^ This work represents an early example of asymmetric halogenation for unfunctionalized olefins with high enantioselectivity. To our delight, high enantioselectivity can also be achieved for bromohydroxylation with H_2_O upon further investigation, giving optically active bromohydrins with up to 98% ee (Scheme 2, eqn (b)).^10^ In our efforts to expand the reaction scope of the asymmetric bromohydroxylation, we have found that cinnamyl alcohols are effective substrates, giving the corresponding bromohydrins with up to 95% ee. Herein, we report our preliminary studies on this subject.

**Scheme 1:**
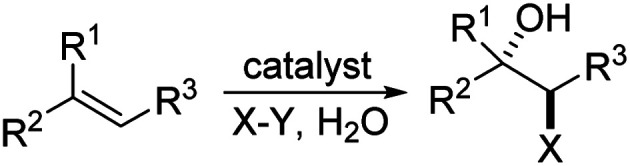
Asymmetric halohydroxylation of olefins.

**Scheme 2:**
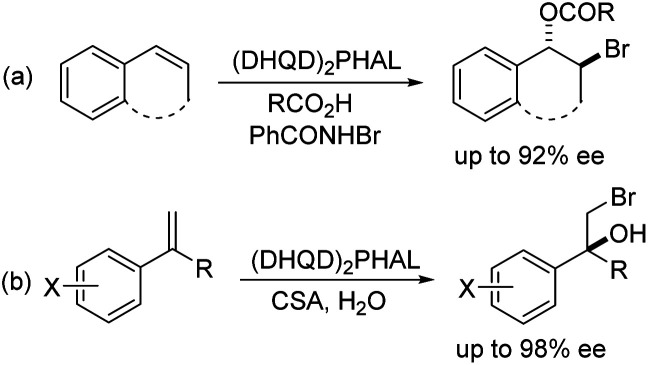
Asymmetric oxybromination of olefins.

Initial studies were carried out with (*E*)-3-(4-bromophenyl)prop-2-en-1-ol (1a) as substrate. Several bromine reagents were examined with 10 mol% (DHQD)_2_PHAL (3a) (Fig. 1) as the catalyst and 10 mol% (−)-camphorsulfonic acid (CSA) as additive in acetone/H_2_O (10 : 1) at −30 °C (Table 1, entries 1–5). *N*-Bromobenzamide gave the highest ee (76%) (Table 1, entry 5). Among the catalysts investigated (Table 1, entries 5–9), (DHQD)_2_PHAL (3a) was the choice of the catalyst with *N*-bromobenzamide. Solvent studies (Table 1, entries 5 and 10–15) showed that the highest ee (83%) was obtained with CH_3_CN/H_2_O (10 : 1) (Table 1, entry 10). Addition of 10 mol% (−)-CSA increased both yield and ee (Table 1, entry 10 *vs.* 16). The best result was obtained with (−)-CSA among the additives examined (Table 1, entry 10 *vs.* entries 17–21). Slightly higher ee (85%) but lower yield was obtained when the reaction temperature was lowered to −40 °C (Table 1, entry 22 *vs.* 10).

**Fig. 1:**
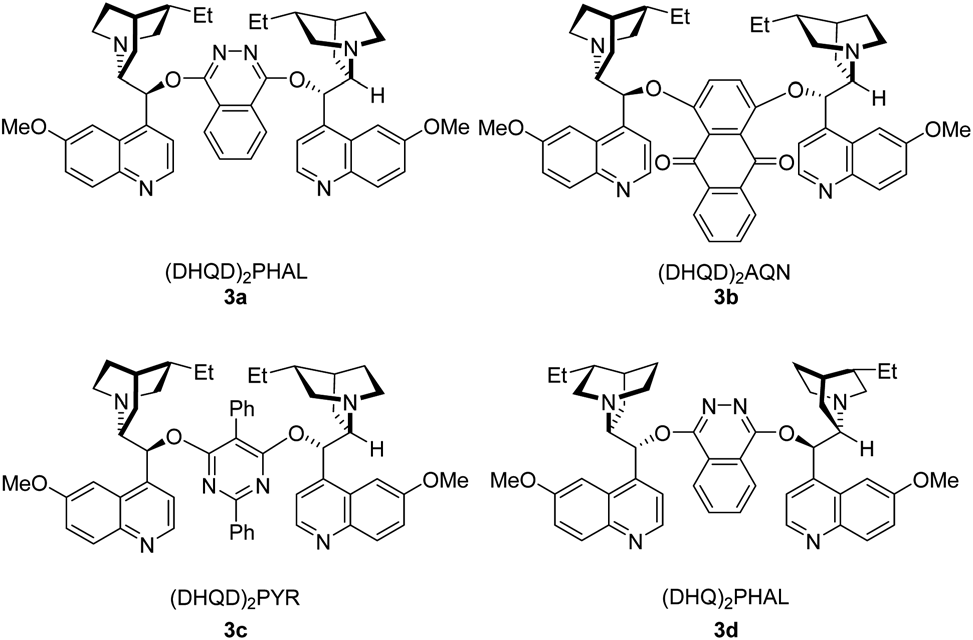
Selected examples of catalyst examined.

**Table 1:** Studies on reaction conditions^a^

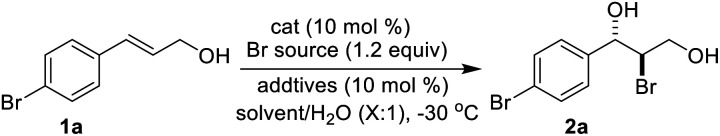					
Entry	Cat.	Br source	Additive	Solvent	Yield^b^ (ee)^c^ %
1	3a	NBS	(−)-CSA	Acetone/H_2_O (10 : 1)	79 (65)
2	3a	DBDMH	(−)-CSA	Acetone/H_2_O (10 : 1)	76 (62)
3	3a	TBCO	(−)-CSA	Acetone/H_2_O (10 : 1)	55 (7)
4	3a	MeCONHBr	(−)-CSA	Acetone/H_2_O (10 : 1)	48 (67)
5	3a	PhCONHBr	(−)-CSA	Acetone/H_2_O (10 : 1)	59 (76)
6	3b	PhCONHBr	(−)-CSA	Acetone/H_2_O (10 : 1)	18 (6)
7	3c	PhCONHBr	(−)-CSA	Acetone/H_2_O (10 : 1)	9 (0)
8	3d	PhCONHBr	(−)-CSA	Acetone/H_2_O (10 : 1)	35 (−57)
9	3e (quinidine)	PhCONHBr	(−)-CSA	Acetone/H_2_O (10 : 1)	31 (0)
10	3a	PhCONHBr	(−)-CSA	CH_3_CN/H_2_O (10 : 1)	70 (83)
11	3a	PhCONHBr	(−)-CSA	EtOAc/H_2_O (10 : 1)	16 (67)
12	3a	PhCONHBr	(−)-CSA	TFE/H_2_O (10 : 1)	43 (51)
13	3a	PhCONHBr	(−)-CSA	DCM/H_2_O (10 : 1)	13 (70)
14^d^	3a	PhCONHBr	(−)-CSA	CH_3_CN/H_2_O (5 : 1)	66 (82)
15^e^	3a	PhCONHBr	(−)-CSA	CH_3_CN/H_2_O (20 : 1)	68 (81)
16	3a	PhCONHBr	—	CH_3_CN/H_2_O (10 : 1)	36 (77)
17	3a	PhCONHBr	(+)-CSA	CH_3_CN/H_2_O (10 : 1)	63 (82)
18	3a	PhCONHBr	PhCO_2_H	CH_3_CN/H_2_O (10 : 1)	34 (77)
19	3a	PhCONHBr	1-NapCO_2_H	CH_3_CN/H_2_O (10 : 1)	32 (77)
20	3a	PhCONHBr	*p*-TsOH	CH_3_CN/H_2_O (10 : 1)	68 (80)
21	3a	PhCONHBr	AlCl_3_	CH_3_CN/H_2_O (10 : 1)	39 (57)
22^f^	3a	PhCONHBr	(−)-CSA	CH_3_CN/H_2_O (10 : 1)	49 (85)

a Reactions were carried out with substrate 1a (0.30 mmol), catalyst (0.030 mmol), additive (0.030 mmol), and Br source (0.36 mmol) in solvent/H_2_O (10 : 1) (3.0 mL + 0.3 mL) at −30 °C for 72 h unless otherwise noted.

b Isolated yield.

c Determined by chiral HPLC analysis.

d CH_3_CN/H_2_O (5 : 1) (3.0 mL + 0.6 mL).

e CH_3_CN/H_2_O (20 : 1) (3.0 mL + 0.15 mL).

f At −40 °C for 168 h.

With the optimized reaction conditions in hand, the substrate scope was subsequently investigated with 10 mol% (DHQD)_2_PHAL (3a), *N*-bromobenzamide (1.2 eq.), and 10 mol% (−)-CSA in CH_3_CN/H_2_O (10 : 1) at −30 °C. As shown in Table 2, the bromohydroxylation can be extended to various cinnamyl alcohols, giving the corresponding bromohydrins in 46–87% yields and 55–95% ee's (Table 2, entries 1–17). The reaction outcome was significantly influenced by the substituent on the phenyl group. In general, the enantioselectivity increased as a substituent was introduced onto the phenyl group. For mono-substituted substrates, it appeared that higher ee was obtained with the *para*-substituent (Table 2, entry 5 *vs.* 6 *vs.* 7). Up to 90% ee was achieved with *p*-Ph substituted cinnamyl alcohol (Table 2, entry 4). For 4-substituted substrates, the enantioselectivity remained similar when a second Me group was introduced to the 3 position (Table 2, entries 9–12). However, significantly higher ee's were obtained when the Me group was introduced to the 2-position, giving the corresponding bromohydrins in 90–95% ee (Table 2, entries 13–17). With 2-Me, 4-Br-substituted cinnamyl alcohol (1m), MeOH was also found to be effective nucleophile, giving the corresponding bromoether (2r) in 75% yield and 90% ee (Table 2, entry 18). A similar ee but lower yield was obtained when the hydroxyl group was replaced with the MeO group, giving the bromohydrin (2s) in 31% yield and 80% ee (Table 2, entry 19). The exact reason for this difference is not clear at this moment.

**Table 2:** Asymmetric bromohydroxylation of cinnamyl alcohols^a^

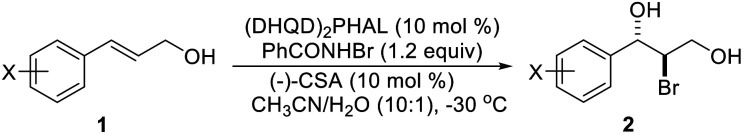				
Entry	Substrate	Product	Yield^b^ (%)	ee^c^ (%)
	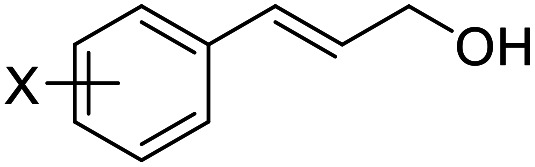	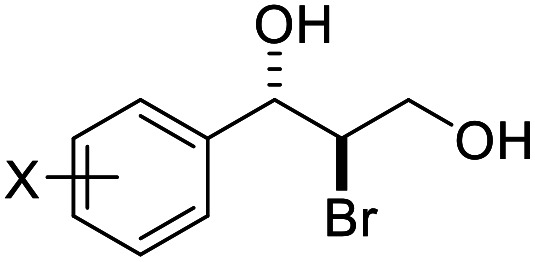		
1	X = *p*-Br, 1a	2a (X-ray)	70	83
2	X = *p*-Cl, 1b	2b	64	80
3	X = *p*-F, 1c	2c	75	76
4^d^	X = *p*-Ph, 1d	2d	87	90
5	X = *p*-Me,1e	2e (X-ray)	76	82
6	X = *m*-Me,1f	2f	71	62
7	X = *o*-Me,1g	2g	77	70
8	X = H,1h	2h	46	55
	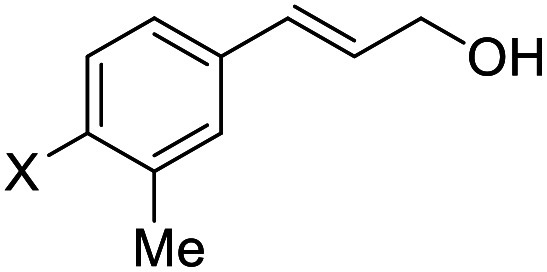	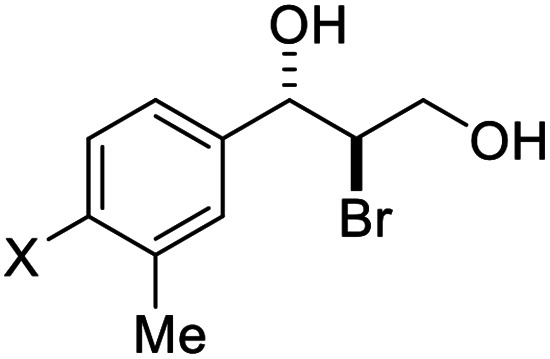		
9	X = Br, 1i	2i	70	80
10	X = Cl, 1j	2j	71	80
11	X = F, 1k	2k	84	80
12	X = Me, 1l	2l	73	82
	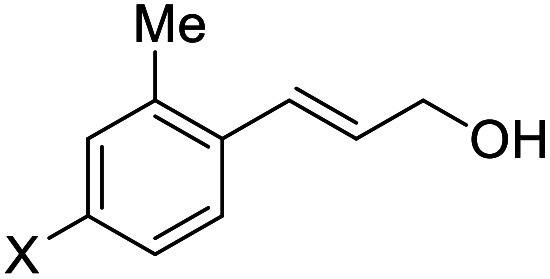	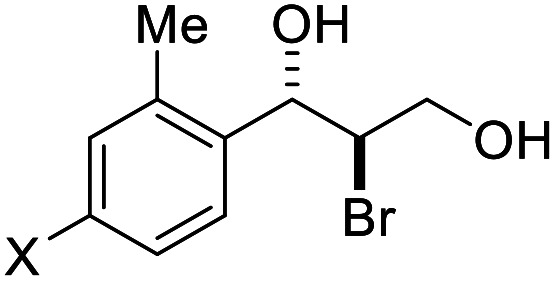		
13	X = Br, 1m	2m	72	95
14	X = Cl, 1n	2n	78	94
15	X = F, 1o	2o	83	91
16	X = Ph, 1p	2p	84	94
17	X = Me, 1q	2q	87	90
18^e^	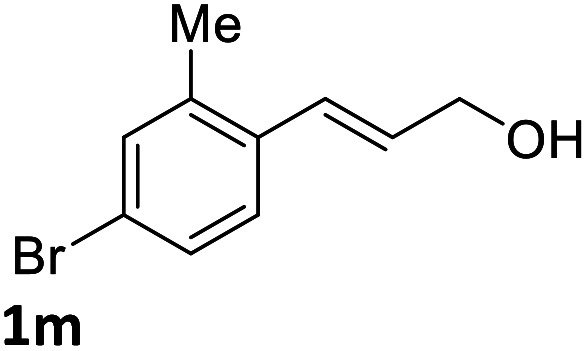	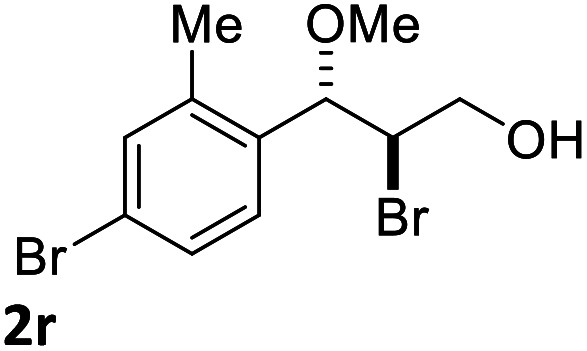	75	90
19	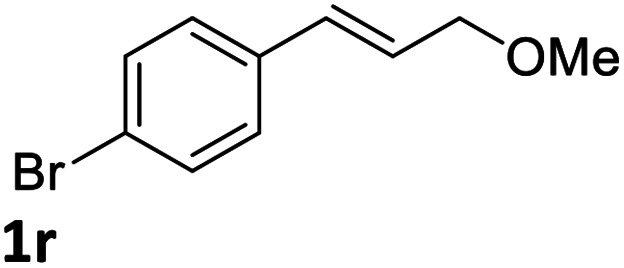	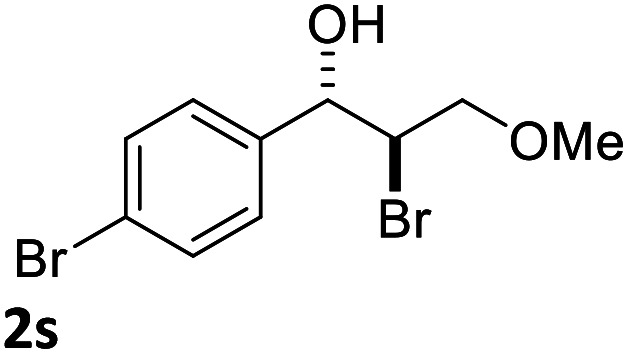	31	80

a Reactions were carried out with substrate 1 (0.50 mmol), (DHQD)_2_PHAL (0.050 mmol), (−)-CSA (0.050 mmol), and PhCONHBr (0.60 mmol) in CH_3_CN (5.0 mL) and water (0.50 mL) at −30 °C for 72 h unless otherwise noted.

b Isolated yield.

c Determined by chiral HPLC analysis. For entry 1, the absolute configuration was determined by comparing the optical rotation of the corresponding epoxide with the reported one^11^ upon treatment with K_2_CO_3_ in acetone (Scheme 3). For others, the absolute configurations were tentatively assigned by analogy.

d The reaction was carried out at −40 °C for 168 h.

e MeOH was used as nucleophile.

The absolute configuration of bromohydrin 2a was determined by converting it to the corresponding epoxide 4 with K_2_CO_3_ (Scheme 3) and comparing the optical rotation of the epoxide with the reported one.^11^ The bromohydroxylation reaction can also be carried out on a relatively large scale. For example, 1.1341 g of bromohydrin 2m was obtained in 70% yield with 95% ee (Scheme 4). As shown in Scheme 5, bromohydrin 2m can be converted to bromoacetal 5 in 86% yield without loss of the ee. Sulfide 6 was obtained in 65% yield and 95% ee when 2m was reacted with sodium thiophenolate.

**Scheme 3:**
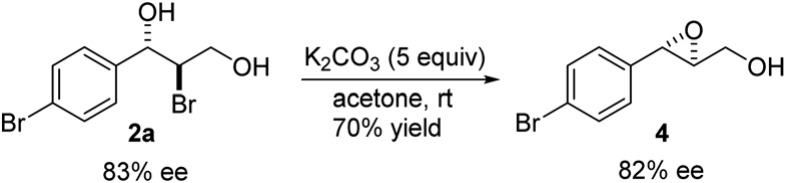
Determination of absolute configuration of bromohydrin 2a.

**Scheme 4:**
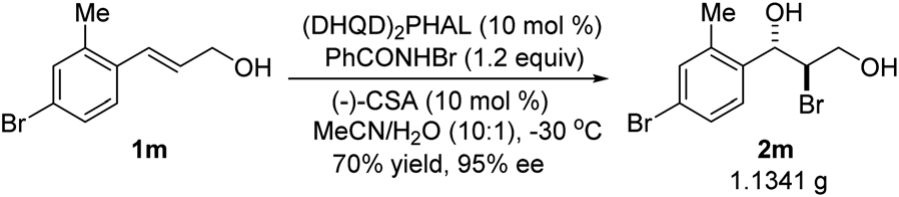
Bromohydroxylation on gram scale.

**Scheme 5:**
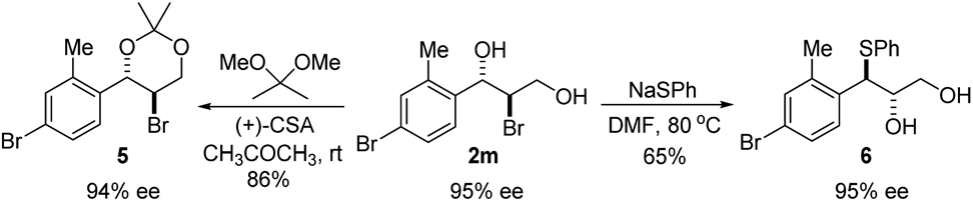
Synthetic transformations of bromohydrin 2m.

Optically active bromoether like 2r could also serve as useful intermediates for further transformations (Scheme 6). Treating 2r with NaN_3_ in DMF at 80 °C gave azide 7 in 50% yield and 90% ee with inversion of configuration. The bromide of 2r could also be converted to chloride 8 in 90% ee while the yield was somewhat low. Epoxide 9 was obtained in 87% yield and 90% ee by treatment of 2r with NaOH in dioxane and water. When 2r was reacted with PhSNa in DMF at 80 °C, sulfide 10 was isolated in 73% yield and 90% ee. The reaction likely proceeded *via* epoxide 9. The synthetic application is further illustrated in Scheme 7. Azide 11 and chloride 12 were obtained from 9 in 80% and 78% yield, respectively, without erosion of the optical activity.^12^

**Scheme 6:**
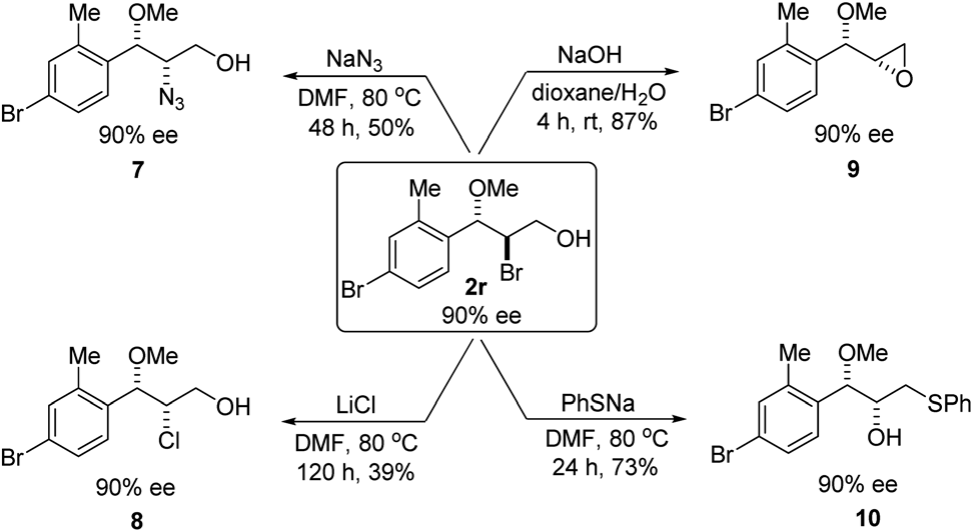
Synthetic transformations of bromoether 2r.

**Scheme 7:**
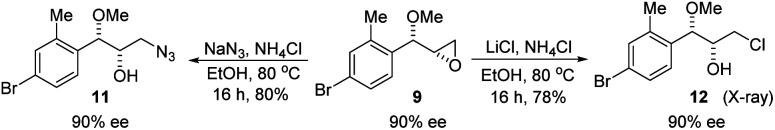
Synthetic transformations of epoxide 9.

A precise understanding of the reaction mechanism awaits further study. As previously described,^10^ two possible transition state models are outlined in Fig. 2. The substrate is likely docked in the chiral pocket through π,π-stacking with quinoline of the catalyst. Such π,π-interaction appeared to be enhanced by the substituents on the phenyl groups, consequently leading to the significant increase of the enantioselectivity. In model A, *N*-bromobenzamide was activated by both the tertiary amine of the catalyst and additive (−)-CSA to increase its electrophility toward the double bond of the reacting substrate. In model B, the tertiary amine of the catalyst could first be protonated by additive (−)-CSA, and *N*-bromobenzamide would subsequently be activated by the resulting quaternary ammonium salt *via* hydrogen bonding.

**Fig. 2:**
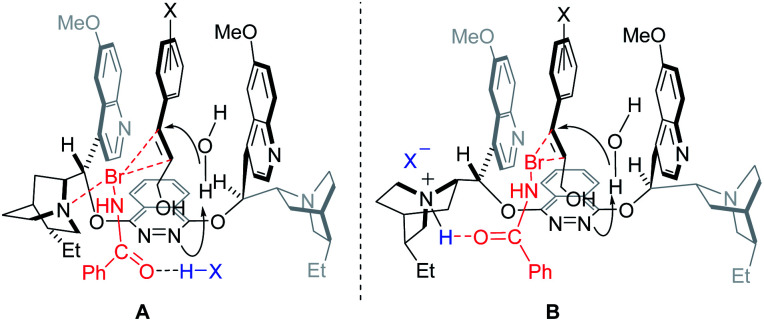
Two possible transition state models.

## Conclusions

In summary, bromohydroxylation of olefins is a classic and important electrophilic addition reaction in organic chemistry. Asymmetric version of this reaction process has been challenging. In this work, we have found that cinnamyl alcohols are effective substrates for asymmetric bromohydroxylation with (DHQD)_2_PHAL as catalyst, (−)-CSA additive, PhCONHBr as bromine source, and H_2_O as nucleophile, providing the corresponding optically active bromohydrins with up to 95% ee. The resulting bromohydrin and related bromoether can be transformed into various highly functionalized molecules with maintained ee's. The current reaction process represents a significant progress in asymmetric bromohydroxylation. Further understanding reaction mechanism, developing more effective catalyst system, and expanding the substrate scope are currently underway.

## Conflicts of interest

There are no conflicts to declare.

## Supplementary Material

RA-011-D1RA02297K-s001

RA-011-D1RA02297K-s002
