# Supplementary material for: Catalytic enantioselective bromohydroxylation of cinnamyl alcohols
Source: RSC Adv. 2021 Apr 7;11(22):13040–6. doi: 10.1039/d1ra02297k (PMC8697332; doi:10.1039/d1ra02297k)

Electronic Supplementary Material (ESI) for RSC Advances.
This journal is © The Royal Society of Chemistry 2021

Catalytic Enantioselective Bromohydroxylation of Cinnamyl Alcohols

Jing Li[a] and Yian Shi*[,a,b]

[a]Institute of Natural and Synthetic Organic Chemistry, Changzhou University, Changzhou 213164, P. R. China.
[b]Department of Chemistry, Colorado State University, Fort Collins, Colorado 80523, USA.

Supporting Information

Table of Contents

Experimental procedures and characterization data S-2

X-Ray structures of compounds **2a**, **2e,** and **12** S-17

Data for determination of enantiomeric excesses S-48

NMR spectra S-62

General Methods. All commercially available reagents were used without further purification unless otherwise noted. All dry solvents were purified with solvent purification system before use. Acetone AR and acetonitrile AR were used directly. Column chromatography was performed on silica gel (300-400 mesh). ^{1}H NMR spectra were recorded on a 400 MHz NMR spectrometer and ^{13}C NMR spectra were recorded on a 100 MHz NMR spectrometer. IR spectra were recorded on a FT-IR spectrometer. Melting points were uncorrected. Cinnamyl alcohol **1h** was purchased from commercial suppliers. Cinnamyl alcohols **1a, 1c** and **1e** were prepared by reacting the corresponding cinnamyl aldehydes with $NaBH_4$.[1] Cinnamyl alcohol **1b** was prepared by the reduction of (*E*)-methyl 3-(4-chlorophenyl)acrylate with DIBAL-H.[1] Cinnamyl alcohols **1d**, **1f-g**, **1i-1r** were prepared by the olefination of corresponding benzaldehydes/ketone with ethyl 2-(diethoxyphosphoryl)acetate and the subsequent reduction with DIBAL-H.[1] Olefin **1r** was prepared from the corresponding alcohol (**1a**) by the methylation with NaH and MeI.[2]

1 X. Huo, R. He, J. Fu, J. Zhang, G. Yang and W. Zhang, Stereoselective and site-specific allylic alkylation of amino acids and small peptides via a Pd/Cu dual catalysis, *J. Am. Chem. Soc.*, 2017, **139**, 9819.

2 C. Kelly, J. Ovian, R. Cywar, T. Gosselin, R. Wiles and N. Leadbeater, Oxidative cleavage of allyl ethers by an oxoammonium salt, *Org. Biomol. Chem.*, 2015, **13**, 4255.

Representative procedure for asymmetric bromohydroxylation of cinnamyl alcohol (Table 2, entry 13). A mixture of $(DHQD)_2PHAL$ (0.0389 g, 0.050 mmol), (-)-CSA (0.0116 g, 0.050 mmol), and *N*-bromobenzamide[1] (0.120 g, 0.60 mmol) in CH_3CN (5.0 mL) and H_2O (0.5 mL) was stirred at -30 °C for 15 min. Cinnamyl alcohol (**1m**) (0.1136 g, 0.50 mmol) was subsequently added. Upon stirring at -30 °C for 72 h, the reaction mixture was quenched with saturated aqueous $Na_2S_2O_3$ (10 mL) at -30 °C, extracted with EtOAc (3x10 mL), dried over Na_2SO_4, filtered, concentrated, and purified by flash chromatography (silica gel, eluent: petroleum ether:ethyl acetate = 2:1) to afford bromohydrin **2m** as colorless oil (0.1168 g, 72% yield, 95% ee).

1 S. Fujisaki, S. Hamura, H. Eguchi and A. Nishida, A facile synthesis of *N*-bromo imides

and amides using sodium bromate and hydrobromic acid (or sodium bromide) in the presence of sulfuric acid, *Bull. Chem. Soc. Jpn.*, 1993, **66**, 2426.

Procedure for gram scale asymmetric bromohydroxylation (Scheme 4). A mixture of $(DHQD)_2PHAL$ (0.3895 g, 0.50 mmol), (-)-CSA (0.1162 g, 0.50 mmol), and *N*-bromobenzamide (1.20 g, 6.0 mmol) in CH_3CN (50.0 mL) and H_2O (5.0 mL) was stirred at -30 ^oC for 15 min. Cinnamyl alcohol (**1m**) (1.1355 g, 5.0 mmol) was subsequently added. Upon stirring at -30 ^oC for 72 h, the reaction mixture was quenched with saturated aqueous $Na_2S_2O_3$ (50 mL) at -30 ^oC, extracted with EtOAc (3x50 mL), washed with saturated NaCl, dried over Na_2SO_4, filtered, concentrated, and purified by flash chromatography (silica gel, eluent: petroleum ether:ethyl acetate = 2:1) to afford bromohydrin **2m** as colorless oil (1.1341 g, 70% yield, 95% ee).

Table 2, entry 1

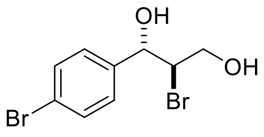

2a

(X-ray structure)

White solid; mp. 68-69 ^oC; $[\alpha]_D^{20}$ = +19.2 (*c* 0.99, $CHCl_3$) (83% ee); IR (film) 3383, 1577, 1010 cm^{-1}; ^1H NMR (400 MHz, $CDCl_3$) δ 7.55-7.48 (m, 2H), 7.32-7.27 (m, 2H), 5.02 (dd, *J* = 6.0, 4.4 Hz, 1H), 4.25 (dt, *J* = 6.0, 4.8 Hz, 1H), 4.02 (ddd, *J* = 12.4, 6.4, 4.8 Hz, 1H), 3.88 (ddd, *J* = 12.4, 6.8, 4.8 Hz, 1H), 3.00 (d, *J* = 4.4 Hz, 1H), 2.37 (dd, *J* = 6.8, 6.4 Hz, 1H); ^{13}C NMR (100 MHz, $CDCl_3$) δ 139.4, 131.9, 128.5, 122.6, 76.3, 64.3, 59.4; HRMS Calcd for $C_9H_{14}Br_2NO_2$ $(M+NH_4)^+$: 325.9386; Found: 325.9386.

Table 2, entry 2

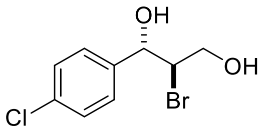

2b

Light yellow oil; $[\alpha]_D^{20}$ = +22.3 (*c* 1.1, $CHCl_3$) (80% ee); IR (film) 3362, 1491, 1014 cm^{-1}; ^1H NMR (400 MHz, $CDCl_3$) δ 7.39-7.32 (m, 4H), 5.04 (dd, *J* = 6.4, 4.0 Hz, 1H), 4.26 (dt, *J* = 6.4, 4.8 Hz, 1H), 4.02 (ddd, *J* = 12.4, 6.4, 4.8 Hz, 1H), 3.88 (ddd, *J* = 12.4, 6.8, 4.8 Hz, 1H), 2.98 (d, *J* = 4.0 Hz, 1H), 2.36 (dd, *J* = 6.8, 6.4 Hz, 1H); ^{13}C NMR (100 MHz, $CDCl_3$) δ 139.0, 134.4, 128.9, 128.2, 76.2, 64.3, 59.2; HRMS Calcd for $C_9H_{14}BrClNO_2$ $(M+NH_4)^+$: 281.9891; Found: 281.9889.

Table 2, entry 3

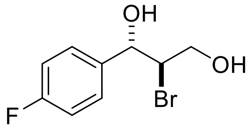

2c

Yellow oil; $[\alpha]_D^{20}$ = +20.6 (*c* 0.89, $CHCl_3$) (76% ee); IR (film) 3383, 1510, 1220 cm^{-1}; ^1H NMR (400 MHz, $CDCl_3$) δ 7.42-7.35 (m, 2H), 7.11-7.04 (m, 2H), 5.04 (dd, *J* = 6.4, 4.0 Hz, 1H), 4.26 (dt, *J* = 6.4, 4.8 Hz, 1H), 4.03 (ddd, *J* = 12.4, 6.4, 4.8 Hz, 1H), 3.89 (ddd, *J* = 12.4, 6.4, 4.8 Hz, 1H), 3.01 (d, *J* = 4.0 Hz, 1H), 2.44 (dd, *J* = 6.8, 6.4 Hz, 1H); ^{13}C NMR (100 MHz, $CDCl_3$) δ 162.8 (d, *J* = 245.6 Hz), 136.3 (d, *J* = 3.2 Hz), 128.5 (d, *J* = 8.3 Hz), 115.7 (d, *J* = 21.5 Hz), 76.3, 64.4, 59.6; HRMS Calcd for $C_9H_{14}BrFNO_2$ $(M+NH_4)^+$: 266.0186; Found: 266.0186.

Table 2, entry 4

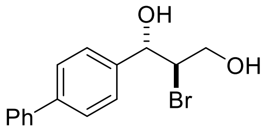

2d

White solid; mp. 83-84 °C; $[\alpha]_D^{20}$ = +19.2 (*c* 1.1, $CHCl_3$) (90% ee); IR (film) 3389, 1540, 1081 cm^{-1}; ^1H NMR (400 MHz, CD_3CN) δ 7.69-7.61 (m, 4H), 7.52-7.43 (m, 4H), 7.41-7.33 (m, 1H), 4.98 (dd, *J* = 6.4, 4.8 Hz, 1H), 4.35 (q, *J* = 5.6 Hz, 1H), 4.00 (d, *J* = 4.8 Hz, 1H), 3.91 (ddd, *J* = 12.4, 6.0, 4.8 Hz, 1H), 3.82 (ddd, *J* = 12.4, 6.4, 4.8 Hz, 1H), 3.30 (t, *J* = 6.0 Hz, 1H); ^{13}C NMR (100 MHz, CD_3CN) δ 141.8, 141.4, 141.3, 129.9, 128.7, 128.5, 127.9, 127.6, 75.4, 64.4, 61.4; HRMS Calcd for $C_{15}H_{19}BrNO_2$ $(M+NH_4)^+$: 324.0594; Found: 324.0593.

Table 2, entry 5

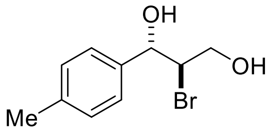

2e

(X-ray structure)

White solid; mp. 57-58 °C; $[\alpha]_D^{20}$ = +26.2 (*c* 0.93, $CHCl_3$) (82% ee); IR (film) 3374, 1578, 1063 cm^{-1}; ^1H NMR (400 MHz, $CDCl_3$) δ 7.29 (d, *J* = 8.0 Hz, 2H), 7.20 (d, *J* = 8.0 Hz, 2H), 5.03 (dd, *J* = 6.0, 4.0 Hz, 1H), 4.31 (dt, *J* = 6.4, 4.8 Hz, 1H), 4.02 (ddd, *J* = 12.4, 6.4, 4.8 Hz, 1H), 3.90 (ddd, *J* = 12.4, 6.4, 4.8 Hz, 1H), 2.77 (d, *J* = 4.0 Hz, 1H), 2.39 (t, *J* = 6.4 Hz, 1H), 2.36 (s, 3H); ^{13}C NMR (100 MHz, $CDCl_3$) δ 138.6, 137.5, 129.5, 126.6, 77.0, 64.5, 60.1, 21.4; HRMS Calcd for $C_{10}H_{17}BrNO_2$ $(M+NH_4)^+$: 262.0437; Found: 262.0437.

Table 2, entry 6

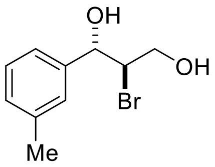

2f

Colorless oil; $[\alpha]_D^{20}$ = +13.7 (*c* 1.0, $CHCl_3$) (62% ee); IR (film) 3447, 1637, 1219 cm^{-1}; ^1H NMR (400 MHz, $CDCl_3$) δ 7.27 (t, *J* = 7.6 Hz, 1H), 7.21 (s, 1H), 7.19 (d, *J* = 7.6 Hz, 1H), 7.15 (d, *J* = 7.6 Hz, 1H), 5.03 (d, *J* = 5.6 Hz, 1H), 4.32 (dt, *J* = 5.6, 5.2 Hz, 1H), 4.02 (dd, *J* = 12.4, 4.8 Hz, 1H), 3.89 (dd, *J* = 12.4, 4.8 Hz, 1H), 2.92 (br s, 1H), 2.46 (br s, 1H), 2.37 (s, 3H); ^{13}C NMR (100 MHz, $CDCl_3$) δ 140.4, 138.5, 129.4, 128.7, 127.3, 123.8, 77.0, 64.4, 59.7, 21.7; HRMS Calcd for $C_{10}H_{17}BrNO_2$ $(M+NH_4)^+$: 262.0437; Found: 262.0434.

Table 2, entry 7

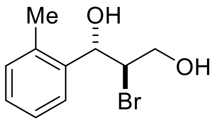

2g

Light yellow oil; $[\alpha]_D^{20}$ = +14.8 (*c* 1.1, $CHCl_3$) (70% ee); IR (film) 3441, 1577, 1383 cm^{-1}; ^1H NMR (400 MHz, $CDCl_3$) δ 7.52 (dd, *J* = 7.2, 1.6 Hz, 1H), 7.30-7.21 (m, 2H), 7.18 (dd, *J* = 7.2, 1.6 Hz, 1H), 5.32 (dd, *J* = 5.6, 4.0 Hz, 1H), 4.32 (dt, *J* = 5.6, 4.8 Hz, 1H), 4.08 (ddd, *J* = 12.4, 6.4, 4.8 Hz, 1H), 3.94 (ddd, *J* = 12.4, 6.8, 4.4 Hz, 1H), 2.81 (d, *J* = 4.0 Hz, 1H), 2.44 (dd, *J* = 6.8, 6.4 Hz, 1H), 2.38 (s, 3H); ^{13}C NMR (100 MHz, $CDCl_3$) δ 138.8, 135.4, 130.9, 128.4, 126.7, 125.8, 73.8, 64.4, 58.6, 19.5; HRMS Calcd for $C_{10}H_{17}BrNO_2$ $(M+NH_4)^+$: 262.0437; Found: 262.0434.

Table 2, entry 8

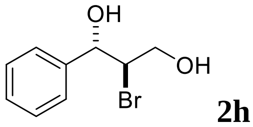

2h

Colorless oil; $[\alpha]_D^{20}$ = +13.0 (*c* 1.0, $CHCl_3$) (55% ee); IR (film) 3439, 1635, 1122 cm^{-1}; ^1H NMR (400 MHz, $CDCl_3$) δ 7.43-7.31 (m, 5H), 5.06 (dd, *J* = 6.0, 4.0 Hz, 1H), 4.32 (dt, *J* = 6.0, 4.8 Hz, 1H), 4.02 (ddd, *J* = 12.4, 6.4, 5.2 Hz, 1H), 3.89 (ddd, *J* = 12.4, 6.8, 4.8 Hz, 1H), 2.95 (d, *J* = 4.0 Hz, 1H), 2.44 (dd, *J* = 6.8, 6.4 Hz, 1H); ^{13}C NMR (100 MHz, $CDCl_3$) δ 140.4, 128.8, 128.7, 126.7, 77.1, 64.4, 60.1; HRMS Calcd for $C_9H_{15}BrNO_2$ $(M+NH_4)^+$: 248.0281; Found: 248.0280.

1 A. K. Macharla, R. C. Nappunni and N. Nama, Regio- and stereoselective hydroxybromination and dibromination of olefins using ammonium bromide and oxone, *Tetrahedron Lett.*, 2012, **53**, 1401.

2 A. J. Burckle, B. Gál, F. J. Seidl, V. H. Vasilev and N. Z. Burns, Enantiospecific solvolytic functionalization of bromochlorides, *J. Am. Chem. Soc.*, 2017, **139**, 13562.

Table 2, entry 9

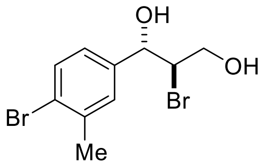

2i

Colorless oil; $[\alpha]_D^{20}$ = +11.9 (*c* 0.99, $CHCl_3$) (80% ee); IR (film) 3417, 1480, 1049 cm^{-1}; ^1H NMR (400 MHz, $CDCl_3$) δ 7.54 (d, *J* = 8.0 Hz, 1H), 7.27 (d, *J* = 1.6 Hz, 1H),

7.09 (dd, *J* = 8.0, 1.6 Hz, 1H), 5.00 (dd, *J* = 5.6, 4.0 Hz, 1H), 4.27 (dt, *J* = 5.6, 4.8 Hz, 1H), 4.02 (dt, *J* = 12.4, 6.4 Hz, 1H), 3.88 (ddd, *J* = 12.0, 6.4, 4.8 Hz, 1H), 2.91 (d, *J* = 4.0 Hz, 1H), 2.42 (s, 3H), 2.33 (t, *J* = 6.4 Hz, 1H); ^{13}C NMR (100 MHz, $CDCl_3$) δ 139.7, 138.4, 132.7, 129.1, 125.7, 125.0, 76.4, 64.3, 59.4, 23.2; HRMS Calcd for $C_{10}H_{16}Br_2NO_2$ $(M+NH_4)^+$: 339.9542; Found: 339.9542.

Table 2, entry 10

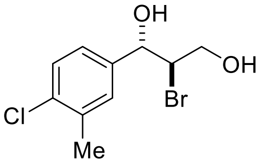

2j

Colorless oil; $[\alpha]_D^{20}$ = +19.4 (*c* 0.96, $CHCl_3$) (80% ee); IR (film) 3454, 1580, 1124 cm^{-1}; ^{1}H NMR (400 MHz, $CDCl_3$) δ 7.35 (d, *J* = 8.0 Hz, 1H), 7.27 (d, *J* = 2.0 Hz, 1H), 7.16 (dd, *J* = 8.0, 2.0 Hz, 1H), 5.00 (dd, *J* = 6.0, 3.6 Hz, 1H), 4.27 (dt *J* = 6.0, 4.8 Hz, 1H), 4.02 (dt, *J* = 12.4, 4.8 Hz, 1H), 3.88 (dt, *J* = 12.4, 4.8 Hz, 1H), 2.94 (d, *J* = 3.6 Hz, 1H), 2.39 (s, 3H), 2.37 (br s, 1H); ^{13}C NMR (100 MHz, $CDCl_3$) δ 139.3, 136.9, 134.8, 129.7, 129.5, 125.8, 76.7, 64.7, 59.6, 20.7; HRMS Calcd for $C_{10}H_{16}BrClNO_2$ $(M+NH_4)^+$: 296.0047; Found: 296.0046.

Table 2, entry 11

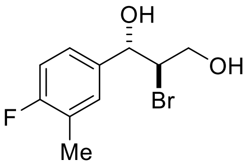

2k

Colorless oil; $[\alpha]_D^{20}$ = +21.9 (*c* 1.0, $CHCl_3$) (80% ee); IR (film) 3444, 1636, 1120 cm^{-1}; ^{1}H NMR (400 MHz, $CDCl_3$) δ 7.22 (dd, *J* = 7.2, 2.0 Hz, 1H), 7.17 (ddd, *J* = 8.4, 4.8, 2.0 Hz, 1H), 7.00 (dd, *J* = 9.2, 8.8 Hz, 1H), 4.99 (dd, *J* = 6.0, 2.4 Hz, 1H), 4.25 (dt, *J* = 6.4, 4.8 Hz, 1H), 4.02 (dt, *J* = 12.4, 4.8 Hz, 1H), 3.89 (dt, *J* = 12.4, 4.8 Hz, 1H), 3.02 (d, *J* = 3.6 Hz, 1H), 2.50 (t, *J* = 6.4 Hz, 1H), 2.29 (d, *J* = 1.6 Hz, 3H); ^{13}C NMR (100 MHz, $CDCl_3$) δ 161.3 (d, *J* = 244.2 Hz), 136.0 (d, *J* = 3.5 Hz), 129.8 (d, *J* = 5.4 Hz), 125.7 (d, *J* = 8.2 Hz), 125.2 (d, *J* = 17.5 Hz), 115.2 (d, *J* = 22.4 Hz), 76.3, 64.4, 59.3, 14.8 (d, *J* = 3.5

Hz); HRMS Calcd for $C_{10}H_{16}BrFNO_2$ $(M+NH_4)^+$: 280.0343; Found: 280.0342.

Table 2, entry 12

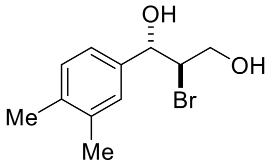

2l

Colorless oil; $[\alpha]_D^{20}$ = +18.4 (*c* 0.96, $CHCl_3$) (82% ee); IR (film) 3450, 1580, 1216 cm^{-1}; ^1H NMR (400 MHz, $CDCl_3$) δ 7.16 (s, 1H), 7.15 (d, *J* = 8.0 Hz, 1H), 7.12 (dd, *J* = 8.0, 1.6 Hz, 1H), 4.99 (d, *J* = 6.4 Hz, 1H), 4.31 (dt, *J* = 6.0, 4.8 Hz, 1H), 4.02 (dd, *J* = 12.4, 4.8 Hz, 1H), 3.91 (dd, *J* = 12.4, 4.8 Hz, 1H), 2.49 (br s, 2H), 2.28 (s, 3H), 2.26 (s, 3H); ^{13}C NMR (100 MHz, $CDCl_3$) δ 138.0, 137.08, 137.06, 130.0, 127.8, 124.1, 77.0, 64.5, 59.7, 20.1, 19.7; HRMS Calcd for $C_{11}H_{19}BrNO_2$ $(M+NH_4)^+$: 276.0594; Found: 276.0590.

Table 2, entry 13

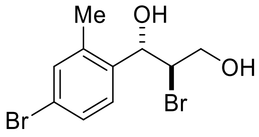

2m

Colorless oil; $[\alpha]_D^{20}$ = +21.7 (*c* 1.2, $CHCl_3$) (95% ee); IR (film) 3440, 1579, 1219 cm^{-1}; ^1H NMR (400 MHz, $CDCl_3$) δ 7.43-7.36 (m, 2H), 7.34 (s, 1H), 5.25 (dd, *J* = 6.0, 4.0 Hz, 1H), 4.25 (dt, *J* = 5.6, 4.8 Hz, 1H), 4.08 (ddd, *J* = 12.4, 6.8, 5.2 Hz, 1H), 3.93 (ddd, *J* = 12.4, 6.4, 4.8 Hz, 1H), 2.88 (d, *J* = 4.0 Hz, 1H), 2.38 (t, *J* = 6.4 Hz, 1H), 2.36 (s, 3H); ^{13}C NMR (100 MHz, $CDCl_3$) δ 137.9, 137.7, 133.6, 129.7, 127.8, 122.3, 73.3, 64.4, 58.1, 19.3; HRMS Calcd for $C_{10}H_{16}Br_2NO_2$ $(M+NH_4)^+$: 339.9542; Found: 339.9542.

Table 2, entry 14

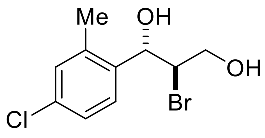

2n

Colorless oil; $[\alpha]_D^{20}$ = +25.8 (*c* 1.1, $CHCl_3$) (94% ee); IR (film) 3387, 1597, 1060 cm^{-1};

^1H NMR (400 MHz, $CDCl_3$) δ 7.43 (d, *J* = 8.4 Hz, 1H), 7.23 (dd, *J* = 8.4, 2.4 Hz, 1H), 7.16 (d, *J* = 2.4 Hz, 1H), 5.24 (d, *J* = 6.0 Hz, 1H), 4.23 (dt, *J* = 6.0, 4.8 Hz, 1H), 4.07 (dd, *J* = 12.4, 3.6 Hz, 1H), 3.91 (dd, *J* = 12.4, 3.6 Hz, 1H), 3.19 (br s, 1H), 2.63 (br s, 1H), 2.34 (s, 3H); ^{13}C NMR (100 MHz, $CDCl_3$) δ 137.41, 137.37, 133.9, 130.6, 127.4, 126.7, 73.2, 64.4, 58.0, 19.4; HRMS Calcd for $C_{10}H_{12}BrClNaO_2$ $(M+Na)^+$: 300.9601; Found: 300.9600.

Table 2, entry 15

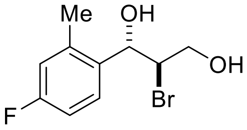

2o

Colorless oil; $[\alpha]_D^{20}$ = +28.7 (*c* 1.1, $CHCl_3$) (91% ee); IR (film) 3387, 1590, 1059 cm^{-1}; ^1H NMR (400 MHz, $CDCl_3$) δ 7.48 (dd, *J* = 8.4, 6.0 Hz, 1H), 6.95 (td, *J* = 8.4, 2.4 Hz, 1H), 6.88 (dd, *J* = 9.6, 2.4 Hz, 1H), 5.27 (dd, *J* = 6.0, 4.0 Hz, 1H), 4.27 (dt, *J* = 6.0, 4.8 Hz, 1H), 4.09 (ddd, *J* = 12.0, 6.4, 4.8 Hz, 1H), 3.94 (ddd, *J* = 12.0, 6.8, 4.8 Hz, 1H), 2.84 (d, *J* = 4.0 Hz, 1H), 2.42 (t, *J* = 6.8 Hz, 1H), 2.38 (s, 3H); ^{13}C NMR (100 MHz, $CDCl_3$) δ 162.4 (d, *J* = 245.3 Hz), 138.0 (d, *J* = 7.8 Hz), 134.7 (d, *J* = 3.4 Hz), 127.8 (d, *J* = 8.5 Hz), 117.4 (d, *J* = 21.0 Hz), 113.5 (d, *J* = 21.1 Hz), 73.3, 64.4, 58.5, 19.6 (d, *J* = 1.0 Hz); HRMS Calcd for $C_{10}H_{12}BrFNaO_2$ $(M+Na)^+$: 284.9897; Found: 284.9890.

Table 2, entry 16

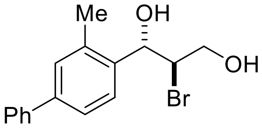

2p

Colorless oil; $[\alpha]_D^{20}$ = +15.2 (*c* 0.82, $CHCl_3$) (94% ee); IR (film) 3395, 1485, 1059 cm^{-1}; ^1H NMR (400 MHz, $CDCl_3$) δ 7.62-7.55 (m, 3H), 7.51-7.39 (m, 4H), 7.39-7.33 (m, 1H), 5.33 (dd, *J* = 6.0, 4.0 Hz, 1H), 4.33 (dt, *J* = 6.0, 4.8 Hz, 1H), 4.11 (dt, *J* = 12.4, 5.2 Hz, 1H), 3.96 (dt, *J* = 12.4, 6.0 Hz, 1H), 3.22 (d, *J* = 2.8 Hz, 1H), 2.76 (t, *J* = 6.4 Hz, 1H), 2.43 (s, 3H); ^{13}C NMR (100 MHz, $CDCl_3$) δ 141.3, 140.7, 137.7, 135.8, 129.7, 129.0, 127.7, 127.3, 126.4, 125.4, 73.8, 64.4, 58.7, 19.7; HRMS Calcd for $C_{16}H_{17}BrNaO_2$

(M+Na)$^+$: 343.0304; Found: 343.0294.

Table 2, entry 17

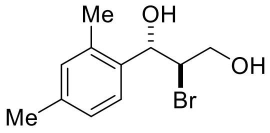

2q

Colorless oil; $[\alpha]_D^{20}$ = +24.3 (*c* 1.0, $CHCl_3$) (90% ee); IR (film) 3394, 1614, 1061 cm^{-1}; ^{1}H NMR (400 MHz, $CDCl_3$) δ 7.39 (d, *J* = 8.0 Hz, 1H), 7.08 (d, *J* = 8.0 Hz, 1H), 7.00 (s, 1H), 5.27 (dd, *J* = 6.0, 3.2 Hz, 1H), 4.30 (dt, *J* = 6.0, 4.8 Hz, 1H), 4.07 (dt, *J* = 12.4, 5.2 Hz, 1H), 3.95 (ddd, *J* = 12.4, 6.0, 4.8 Hz, 1H), 2.70 (d, *J* = 4.0 Hz, 1H), 2.46 (t, *J* = 6.4 Hz, 1H), 2.34 (s, 3H), 2.32 (s, 3H); ^{13}C NMR (100 MHz, $CDCl_3$) δ 138.0, 135.6, 135.1, 131.5, 127.2, 125.5, 73.6, 64.3, 58.6, 21.1, 19.2; HRMS Calcd for $C_{11}H_{15}BrNaO_2$ (M+Na)$^+$: 281.0148; Found: 281.0144.

Table 2, entry 18

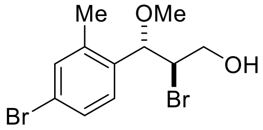

2r

Colorless oil; $[\alpha]_D^{20}$ = +48.0 (*c* 0.99, $CHCl_3$) (90% ee); IR (film) 3438, 1590, 1088 cm^{-1}; ^{1}H NMR (400 MHz, $CDCl_3$) δ 7.38 (dd, *J* = 8.4, 2.0 Hz, 1H), 7.34 (d, *J* = 1.6 Hz, 1H), 7.24 (d, *J* = 8.4 Hz, 1H), 4.73 (d, *J* = 7.2 Hz, 1H), 4.15 (dt, *J* = 7.6, 4.8 Hz, 1H), 4.04 (ddd, *J* = 12.4, 7.2, 4.8 Hz, 1H), 3.94 (ddd, *J* = 12.4, 6.4, 4.8 Hz, 1H), 3.22 (s, 3H), 2.64 (dd, *J* = 7.2, 6.4 Hz, 1H), 2.37 (s, 3H); ^{13}C NMR (100 MHz, $CDCl_3$) δ 139.1, 136.0, 133.6, 129.8, 128.4, 122.3, 82.2, 64.8, 57.6, 57.3, 19.5; HRMS Calcd for $C_{11}H_{18}Br_2NO_2$ (M+NH$_4$)$^+$: 353.9699; Found: 353.9696.

Table 2, entry 19

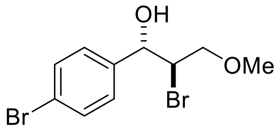

2s

Yellow oil; $[\alpha]_D^{20}$ = +14.1 (*c* 0.98, $CHCl_3$) (80% ee); IR (film) 3447, 1577, 1219 cm^{-1};

^{1}H NMR (400 MHz, $CDCl_3$) δ 7.50 (d, *J* = 8.4 Hz, 2H), 7.27 (d, *J* = 9.2 Hz, 2H), 4.97 (d, *J* = 6.0 Hz, 1H), 4.26 (dt, *J* = 6.0, 5.2 Hz, 1H), 3.79 (dd, *J* = 10.4, 5.2 Hz, 1H), 3.63 (dd, *J* = 10.4, 6.0 Hz, 1H), 3.46 (br s, 1H), 3.41 (s, 3H); ^{13}C NMR (100 MHz, $CDCl_3$) δ 139.5, 131.7, 128.7, 122.4, 76.2, 74.4, 59.4, 54.9; HRMS Calcd for $C_{10}H_{16}Br_2NO_2$ $(M+NH_4)^+$: 339.9542; Found: 339.9543.

Determination of the absolute configuration of bromohydrin 2a (Scheme 3)

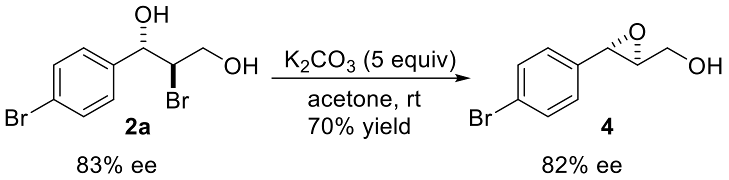

To a solution of bromohydrin **2a** (0.1081 g, 0.35 mmol) in acetone (4 mL) was added K_2CO_3 (0.2420 g, 1.75 mmol). Upon stirring at rt overnight, the reaction mixture was filtered through a silica gel plug (washed with CH_2Cl_2) and concentrated to give epoxide **4** as white solid (0.0563 g, 70%). mp. 57-58 °C. $[\alpha]_D^{20}$ = -24.6 (*c* 0.80, $CHCl_3$) (82% ee) {lit.[1] $[\alpha]_D^{20}$ = -35.2 (*c* 2.0, $CHCl_3$)}; IR (film) 3447, 1636, 1093 cm^{-1}; ^{1}H NMR (400 MHz, $CDCl_3$) δ 7.48 (d, *J* = 8.4 Hz, 2H), 7.16 (d, *J* = 8.4 Hz, 2H), 4.04 (ddd, *J* = 12.8, 5.2, 2.4 Hz, 1H), 3.90 (d, *J* = 2.0 Hz, 1H), 3.81 (ddd, *J* = 12.8, 8.0, 3.6 Hz, 1H), 3.17 (dt, *J* = 3.6, 2.0 Hz, 1H), 1.82 (dd, *J* = 8.0, 5.2 Hz, 1H); ^{13}C NMR (100 MHz, $CDCl_3$) δ 136.0, 131.9, 127.6, 122.4, 62.7, 61.2, 55.1. HRMS Calcd for $C_9H_9BrNaO_2$ $(M+Na)^+$: 250.9678; Found: 250.9675.

1 Y. Gao, R. M. Hanson, J. M. Klunder, S. Y. Ko, H. Masamune and K. B. Sharpless, Catalytic asymmetric epoxidation and kinetic resolution modified procedures including in situ derivatization, *J. Am. Chem. Soc.*, 1987, **109**, 5765.

Synthetic transformations of bromohydrin 2m (Scheme 5)

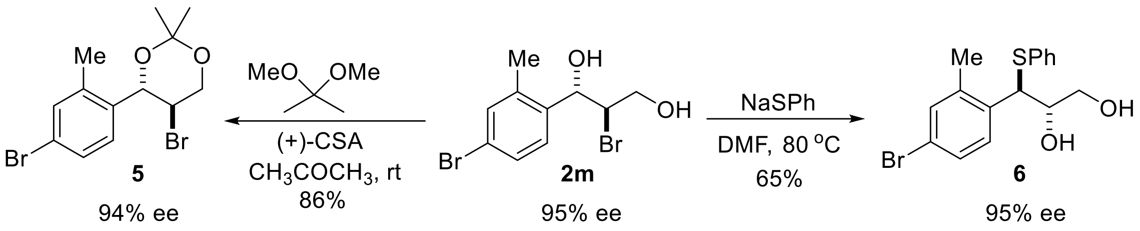

Procedure for acetal 5. A mixture of bromohydrin **2m** (0.1168 g, 0.36 mmol), 2,2-dimethoxypropane (0.9 mL), and (+)-CSA (0.0167 g, 0.072 mmol) in acetone (0.9 mL) was stirred at rt for 2 h, concentrated, and purified by flash chromatography (silica gel, eluent: petroleum ether:ethyl acetate = 18:1) to give acetal **5** as colorless oil (0.1132 g, 86%). $[\alpha]_D^{20}$ = -9.5 (*c* 1.2, $CHCl_3$) (94% ee); IR (film) 3439, 1593, 1089 cm^{-1}; ^1H NMR (400 MHz, $CDCl_3$) δ 7.40 (dd, *J* = 8.0, 2.0 Hz, 1H), 7.35 (d, *J* = 1.6 Hz, 1H), 7.32 (d, *J* = 8.0 Hz, 1H), 5.16-5.07 (m, 1H), 4.20-4.10 (m, 3H), 2.44 (s, 3H), 1.65 (s, 3H), 1.46 (s, 3H); ^{13}C NMR (100 MHz, $CDCl_3$) δ 139.1, 135.6, 133.5, 129.7, 128.9, 122.6, 100.2, 72.8, 65.9, 47.1, 29.5, 19.6, 18.8; HRMS Calcd for $C_{13}H_{20}Br_2NO_2$ $(M+NH_4)^+$: 379.9855; Found: 379.9854.

S. Kiyooka, K. A. Shahid, Efficient enantio- and diastereoselective synthesis of enantiopure syn-α-bromo-β-hydroxy-α-methylpropionate esters and their cis-α,β-epoxy derivatives based on a chiral oxazaborolidinone-promoted asymmetric aldol reaction, *Tetrahedron: Asymmetry*, 2000, **11**, 1537.

Procedure for sulfide 6. A mixture of bromohydrin **2m** (0.0850 g, 0.26 mmol) and NaSPh (0.0687 g, 0.52 mmol) in DMF (3 mL) was stirred at 80 °C for 1 h, quenched with water (6 mL), extracted with ethyl acetate (2x8 mL), washed with water and brine, dried over Na_2SO_4, filtered, concentrated, and purified by flash chromatography (silica gel, eluent: petroleum ether:ethyl acetate = 2:1) to give sulfide **6** as colorless oil (0.0597 g, 65%). $[\alpha]_D^{20}$ = -93.6 (*c* 0.87, $CHCl_3$) (95% ee); IR (film) 3409, 1479, 1088 cm^{-1}; ^1H NMR (400 MHz, $CDCl_3$) δ 7.34-7.19 (m, 8H), 4.48 (d, *J* = 8.0 Hz, 1H), 4.08-4.01 (m, 1H), 3.86 (ddd, *J* = 11.2, 6.4, 3.6 Hz, 1H), 3.75 (dt, *J* = 11.2, 5.6 Hz, 1H), 2.39 (d, *J* = 4.0 Hz, 1H), 2.24 (s, 3H), 2.03 (t, *J* = 6.0 Hz, 1H); ^{13}C NMR (100 MHz, $CDCl_3$) δ 139.2, 136.1,

133.5, 133.41, 133.36, 129.9, 129.7, 129.3, 128.3, 121.4, 73.7, 64.3, 51.2, 19.7; HRMS Calcd for $C_{16}H_{17}BrNaO_2S$ $(M+Na)^+$: 375.0025; Found: 375.0026.

D. Huang, X. Liu, L. Li, Y. Cai, W. Liu and Y. Shi, Enantioselective bromoaminocyclization of allyl *N*-tosylcarbamates catalyzed by a chiral phosphine-Sc(OTf)$_3$ complex, *J. Am. Chem. Soc.*, 2013, **135**, 8101.

Synthetic transformations of bromoether 2r (Scheme 6)

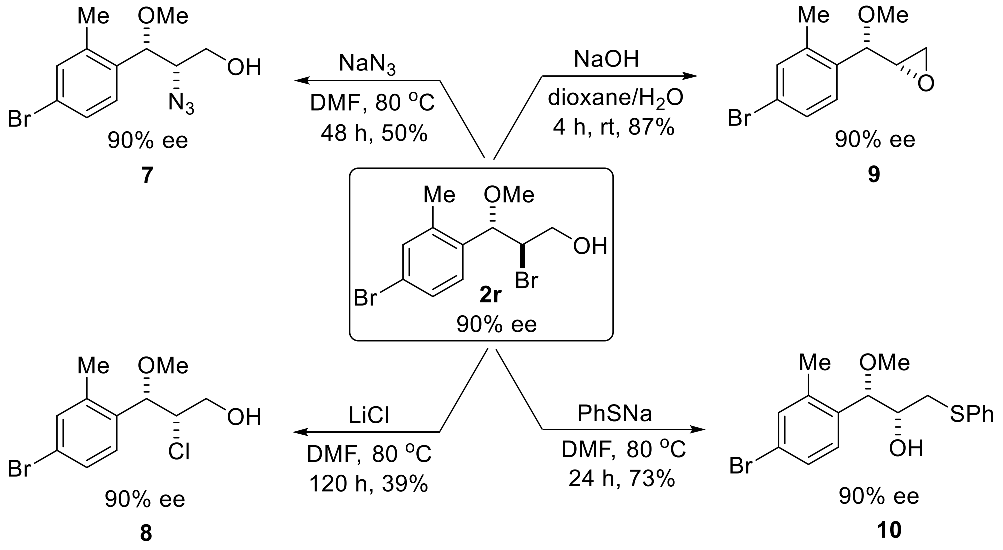

Procedure for azide 7. A mixture of bromoether **2r** (0.0843 g, 0.25 mmol) and NaN_3 (0.0650 g, 1.0 mmol) in DMF (2 mL) was stirred at 80 °C for 48 h, quenched with water (10 mL), extracted with ethyl acetate (3x10 mL), washed with water and brine, dried over Na_2SO_4, filtered, concentrated, and purified by flash chromatography (silica gel, eluent: petroleum ether:ethyl acetate = 8:1) to give azide **7** as colorless oil (0.0378 g, 50%). $[\alpha]_D^{20}$ = +97.2 (*c* 1.3, $CHCl_3$) (90% ee); IR (film) 3438, 2104, 1590, 1098 cm^{-1}; ^1H NMR (400 MHz, $CDCl_3$) δ 7.38 (dd, *J* = 8.4, 1.6 Hz, 1H), 7.34 (d, *J* = 1.6 Hz, 1H), 7.26 (d, *J* = 8.4 Hz, 1H), 4.59 (d, *J* = 7.2 Hz, 1H), 3.64 (ddd, *J* = 7.2, 5.6, 4.0 Hz, 1H), 3.52 (ddd, *J* = 11.6, 6.0, 3.6 Hz, 1H), 3.34 (dt, *J* = 11.6, 6.0 Hz, 1H), 3.23 (s, 3H), 2.35 (s, 3H), 1.90 (t, *J* = 6.0 Hz, 1H), ^{13}C NMR (100 MHz, $CDCl_3$) δ 138.9, 135.3, 133.7, 129.9, 129.0, 122.3, 80.6, 67.8, 62.1, 56.9, 19.3; HRMS Calcd for $C_{11}H_{15}BrN_3O_2$ $(M+H)^+$: 300.0342; Found: 300.0343.

D. Huang, X. Liu, L. Li, Y. Cai, W. Liu and Y. Shi, Enantioselective

bromoaminocyclization of allyl *N*-tosylcarbamates catalyzed by a chiral phosphine-Sc(OTf)$_3$ complex, *J. Am. Chem. Soc.*, 2013, **135**, 8101.

Procedure for chloride 8. A mixture of bromoether **2r** (0.1101 g, 0.325 mmol) and LiCl (0.0551 g, 1.3 mmol) in DMF (3 mL) was stirred at 80 ^{o}C for 120 h, quenched with water (10 mL), extracted with ethyl acetate (3x10 mL), washed with water and brine, dried over Na_2SO_4, filtered, concentrated, and purified by flash chromatography (silica gel, eluent: petroleum ether:ethyl acetate = 8:1) to give chloride **8** as colorless oil (0.0375 g, 39%). $[\alpha]_D^{20}$ = +43.8 (*c* 0.86, $CHCl_3$) (90% ee); IR (film) 3439, 1591, 1099 cm^{-1}; ^{1}H NMR (400 MHz, $CDCl_3$) δ 7.38 (dd, *J* = 8.4, 2.0 Hz, 1H), 7.34 (d, *J* = 2.0 Hz, 1H), 7.26 (d, *J* = 8.4 Hz, 1H), 4.68 (d, *J* = 6.4 Hz, 1H), 4.11 (dt, *J* = 6.0, 5.2 Hz, 1H), 3.77 (ddd, *J* = 11.6, 6.0, 4.8 Hz, 1H), 3.61 (dt, *J* = 11.6, 6.0 Hz, 1H), 3.25 (s, 3H), 2.36 (s, 3H), 2.15 (t, *J* = 6.4 Hz, 1H), ^{13}C NMR (100 MHz, $CDCl_3$) δ 138.8, 135.1, 133.7, 129.7, 129.3, 122.4, 79.8, 66.2, 64.2, 57.3 19.2; HRMS Calcd for $C_{11}H_{15}BrClO_2$ $(M+H)^+$: 292.9938; Found: 292.9937.

D. Huang, X. Liu, L. Li, Y. Cai, W. Liu and Y. Shi, Enantioselective bromoaminocyclization of allyl *N*-tosylcarbamates catalyzed by a chiral phosphine-Sc(OTf)$_3$ complex, *J. Am. Chem. Soc.*, 2013, **135**, 8101.

Procedure for epoxide 9. A mixture of bromoether **2r** (0.3101 g, 0.92 mmol) and NaOH (0.1840 g, 4.6 mmol) in dioxane (4 mL) and water (2 mL) was stirred at rt for 4 h. The reaction mixture was diluted with water (10 mL), extracted with ether (3x10 mL), washed with water and brine, dried over Na_2SO_4, filtered, concentrated, and purified by flash chromatography (silica gel, eluent: petroleum ether:ethyl acetate = 14:1) to give epoxide **9** as colorless oil (0.2047 g, 87%). $[\alpha]_D^{20}$ = +30.3 (*c* 1.3, $CHCl_3$) (90% ee); IR (film) 3443, 1590, 1082 cm^{-1}; ^{1}H NMR (400 MHz, $CDCl_3$) δ 7.35 (dd, *J* = 8.4, 2.0 Hz, 1H), 7.32 (d, *J* = 1.6 Hz, 1H), 7.25 (d, *J* = 8.0 Hz, 1H), 4.19 (d, *J* = 6.0 Hz, 1H), 3.30 (s, 3H), 3.21 (ddd, *J* = 6.0, 4.4, 2.8 Hz, 1H), 2.71 (dd, *J* = 4.8, 4.4 Hz, 1H), 2.54 (dd, *J* = 4.8, 2.8 Hz, 1H), 2.31 (s, 3H); ^{13}C NMR (100 MHz, $CDCl_3$) δ 138.5, 135.1, 133.6, 129.6, 129.1, 122.1, 81.0, 57.2, 54.4, 44.2, 19.4; HRMS Calcd for $C_{11}H_{14}BrO_2$ $(M+H)^+$:

257.0172; Found: 257.0171.

D. X. Hu, G. M. Shibuya and N. Z. Burns, Catalytic enantioselective dibromination of allylic alcohols, *J. Am. Chem. Soc.*, 2013, **135**, 12960.

Procedure for sulfide 10. A mixture of bromoether **2r** (0.1041 g, 0.31 mmol) and NaSPh (0.0819 g, 0.62 mmol) in DMF (3 mL) was stirred at 80 °C for 24 h, quenched with water (10 mL), extracted with ethyl acetate (3x10 mL), washed with water and brine, dried over Na_2SO_4, filtered, concentrated, and purified by flash chromatography (silica gel, eluent: petroleum ether:ethyl acetate = 8:1) to give sulfide **10** as colorless oil (0.0828 g, 73%). $[\alpha]_D^{20}$ = +45.9 (*c* 0.99, $CHCl_3$) (90% ee); IR (film) 3439, 1589, 1100 cm^{-1}; ^1H NMR (400 MHz, $CDCl_3$) δ 7.34 (dd, *J* = 8.0, 2.0 Hz, 1H), 7.32 (d, *J* = 1.6 Hz, 1H), 7.26-7.21 (m, 4H), 7.20-7.13 (m, 2H), 4.51 (d, *J* = 5.6 Hz, 1H), 3.84-3.76 (m, 1H), 3.19 (s, 3H), 3.05 (dd, *J* = 13.6, 5.2 Hz, 1H), 2.94 (dd, *J* = 13.6, 6.8 Hz, 1H), 2.81 (d, *J* = 4.4 Hz, 1H), 2.28 (s, 3H); ^{13}C NMR (100 MHz, $CDCl_3$) δ 138.7, 135.8, 135.4, 133.6, 129.5, 129.3, 129.2, 129.0, 126.4, 121.9, 80.7, 73.3, 57.0, 36.6, 19.3; HRMS Calcd for $C_{17}H_{23}BrNO_2S$ $(M+NH_4)^+$: 384.0627; Found: 384.0626.

D. Huang, X. Liu, L. Li, Y. Cai, W. Liu and Y. Shi, Enantioselective bromoaminocyclization of allyl *N*-tosylcarbamates catalyzed by a chiral phosphine-Sc(OTf)$_3$ complex, *J. Am. Chem. Soc.*, 2013, **135**, 8101.

Synthetic transformations of epoxide 9 (Scheme 7)

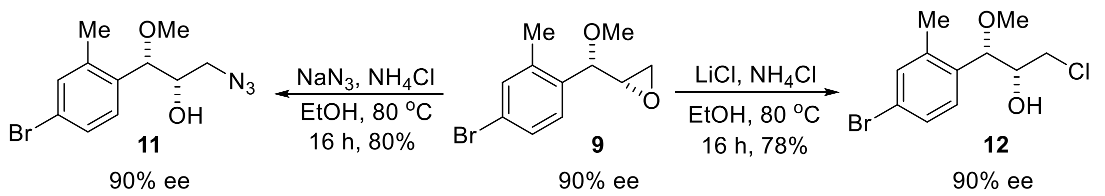

Procedure for azide 11. A mixture of epoxide **9** (0.0514 g, 0.20 mmol), NaN_3 (0.0520 g, 0.80 mmol), and NH_4Cl (0.0134 g, 0.25 mmol) in EtOH (2 mL) was stirred at 80 °C for 16 h. The reaction mixture was concentrated, diluted with water (5 mL), extracted with ether (4x5 mL), washed with brine, dried over Na_2SO_4, filtered, concentrated, and purified by flash chromatography (silica gel, eluent: petroleum ether:ethyl acetate = 8:1) to give

azide **11** as colorless oil (0.0478 g, 80%). $[\alpha]_D^{20}$ = +8.3 (*c* 1.1, $CHCl_3$) (90% ee); IR (film) 3438, 2101, 1590, 1088 cm^{-1}; ^{1}H NMR (400 MHz, $CDCl_3$) δ 7.38-7.32 (m, 2H), 7.17 (d, *J* = 8.0 Hz, 1H), 4.44 (d, *J* = 7.6 Hz, 1H), 3.90-3.82 (m, 1H), 3.32 (dd, *J* = 13.2, 4.0 Hz, 1H), 3.22 (s, 3H), 3.04 (d, *J* = 2.4 Hz, 1H), 2.98 (dd, *J* = 13.2, 5.2 Hz, 1H), 2.36 (s, 3H); ^{13}C NMR (100 MHz, $CDCl_3$) δ 139.2, 134.8, 133.8, 129.8, 129.1, 122.3, 80.4, 74.6, 56.9, 52.1, 19.3; HRMS Calcd for $C_{11}H_{15}BrN_3O_2$ $(M+H)^+$: 300.0342; Found: 300.0341.

J. H. Lee, S. Gupta, W. Jeong, Y. H. Rhee and J. Park, Characterization and utility of *N*-substituted imines synthesized from alkyl azides by Ruthenium catalysis, *Angew. Chem., Int. Ed.*, 2012, **51**, 10851.

Procedure for chloride 12. A mixture of epoxide **9** (0.0514 g, 0.20 mmol), LiCl (0.0339 g, 0.80 mmol), and NH_4Cl (0.0134 g, 0.25 mmol) in EtOH (2 mL) was stirred at 80 ^oC for 16 h. The reaction mixture was concentrated, diluted with water (5 mL), extracted with ether (4x5 mL), washed with brine, dried over Na_2SO_4, filtered, concentrated, and purified by flash chromatography (silica gel, eluent: petroleum ether:ethyl acetate = 8:1) to give chloride **12** as white solid (0.0456 g, 78%). mp. 61-62 ^oC. $[\alpha]_D^{20}$ = +34.7 (*c* 0.87, $CHCl_3$) (90% ee); IR (film) 3443, 1590, 1088 cm^{-1}; ^{1}H NMR (400 MHz, $CDCl_3$) δ 7.39-7.33 (m, 2H), 7.21 (d, *J* = 8.0 Hz, 1H), 4.56 (d, *J* = 5.6 Hz, 1H), 3.94-3.87 (m, 1H), 3.60 (dd, *J* = 11.2, 5.2 Hz, 1H), 3.36 (dd, *J* = 11.2, 5.2 Hz, 1H), 3.25 (s, 3H), 2.95 (d, *J* = 4.4 Hz, 1H), 2.37 (s, 3H); ^{13}C NMR (100 MHz, $CDCl_3$) δ 139.0, 134.8, 133.8, 129.7, 129.1, 122.2, 79.6, 74.3, 57.1, 45.1, 19.3; HRMS Calcd for $C_{11}H_{18}BrClNO_2$ $(M+NH_4)^+$: 310.0204; Found: 310.0199.

The X-ray structure of compound **2a**

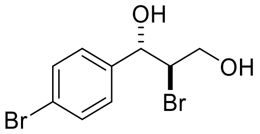

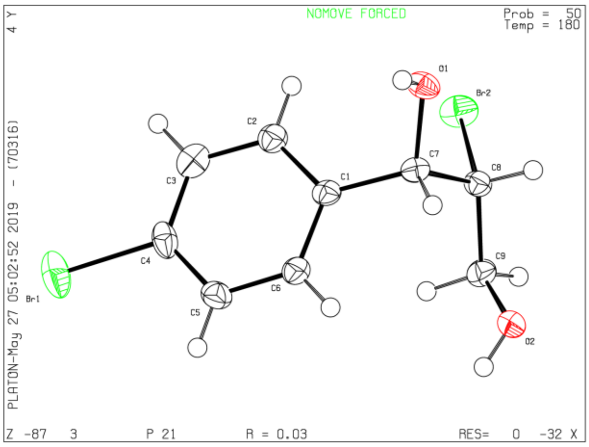

Table 1. Crystal data and structure refinement for **2a**.

Identification code	**2a**	
Empirical formula	$C_9H_{10}Br_2O_2$	
Formula weight	309.99	
Temperature	179.99(10) K	
Wavelength	0.71073 Å	
Crystal system	Monoclinic	
Space group	$P2_1$	
Unit cell dimensions	a = 5.9149(2) Å	α= 90°.
	b = 8.0004(2) Å	β= 101.679(3)°.
	c = 11.2008(3) Å	γ = 90°.
Volume	519.07(3) Å^3	
Z	2	
Density (calculated)	1.983 Mg/m^3	
Absorption coefficient	7.773 mm^{-1}	
F(000)	300	
Crystal size	0.43 x 0.08 x 0.05 mm^3	
Theta range for data collection	3.152 to 27.481°.	
Index ranges	-7<=h<=7, -10<=k<=10, -14<=l<=14	
Reflections collected	11171	
Independent reflections	2382 [R(int) = 0.0436]	
Completeness to theta = 25.242°	99.9 %	
Absorption correction	Semi-empirical from equivalents	
Max. and min. transmission	1.00000 and 0.47158	
Refinement method	Full-matrix least-squares on F^2	
Data / restraints / parameters	2382 / 1 / 120	
Goodness-of-fit on F2	1.058	
Final R indices [I>2sigma(I)]	R1 = 0.0300, wR2 = 0.0693	
R indices (all data)	R1 = 0.0329, wR2 = 0.0706	
Absolute structure parameter	0.001(11)	
Extinction coefficient	n/a	
Largest diff. peak and hole	0.735 and -0.492 e.Å^{-3}	

Table 2. Atomic coordinates ($x10^4$) and equivalent isotropic displacement parameters ($Å^2x10^3$) for **2a**. U(eq) is defined as one third of the trace of the orthogonalized U^{ij} tensor.

	x	y	z	U(eq)
Br(1)	522(1)	8839(1)	5193(1)	40(1)
Br(2)	6064(1)	1164(1)	7144(1)	30(1)
O(1)	7843(5)	3799(5)	9235(3)	24(1)
O(2)	1448(6)	1610(5)	9407(3)	27(1)
C(1)	4271(9)	4942(7)	8026(5)	20(1)
C(2)	5227(10)	5434(7)	7034(5)	23(1)
C(3)	4175(10)	6594(7)	6218(5)	28(1)
C(4)	2083(9)	7289(7)	6350(5)	26(1)
C(5)	1104(9)	6843(7)	7332(5)	26(1)
C(6)	2210(9)	5691(7)	8158(5)	24(1)
C(7)	5387(8)	3646(7)	8936(4)	20(1)
C(8)	4831(8)	1839(7)	8562(5)	20(1)
C(9)	2263(9)	1432(8)	8295(5)	24(1)

Table 3. Bond lengths [Å] and angles [°] for **2a**.

Br(1)-C(4)	1.894(5)
Br(2)-C(8)	1.954(5)
O(1)-C(7)	1.429(5)
O(1)-H(1)	0.8200
O(2)-C(9)	1.431(6)
O(2)-H(2)	0.8200
C(1)-C(6)	1.393(7)
C(1)-C(2)	1.402(7)
C(1)-C(7)	1.509(7)
C(2)-C(3)	1.362(8)
C(2)-H(2A)	0.9300
C(3)-C(4)	1.392(8)
C(3)-H(3)	0.9300
C(4)-C(5)	1.389(8)
C(5)-C(6)	1.374(8)
C(5)-H(5)	0.9300
C(6)-H(6)	0.9300
C(7)-C(8)	1.522(7)
C(7)-H(7)	0.9800
C(8)-C(9)	1.523(7)
C(8)-H(8)	0.9800
C(9)-H(9A)	0.9700
C(9)-H(9B)	0.9700
C(7)-O(1)-H(1)	109.5
C(9)-O(2)-H(2)	109.5
C(6)-C(1)-C(2)	117.7(5)
C(6)-C(1)-C(7)	119.8(5)
C(2)-C(1)-C(7)	122.5(5)
C(3)-C(2)-C(1)	121.4(5)
C(3)-C(2)-H(2A)	119.3
C(1)-C(2)-H(2A)	119.3
C(2)-C(3)-C(4)	119.7(5)
C(2)-C(3)-H(3)	120.1
C(4)-C(3)-H(3)	120.1
C(5)-C(4)-C(3)	120.2(5)
C(5)-C(4)-Br(1)	119.1(4)

C(3)-C(4)-Br(1)	120.6(4)
C(6)-C(5)-C(4)	119.2(5)
C(6)-C(5)-H(5)	120.4
C(4)-C(5)-H(5)	120.4
C(5)-C(6)-C(1)	121.6(5)
C(5)-C(6)-H(6)	119.2
C(1)-C(6)-H(6)	119.2
O(1)-C(7)-C(1)	112.6(4)
O(1)-C(7)-C(8)	107.4(4)
C(1)-C(7)-C(8)	115.3(4)
O(1)-C(7)-H(7)	107.1
C(1)-C(7)-H(7)	107.1
C(8)-C(7)-H(7)	107.1
C(7)-C(8)-C(9)	114.1(4)
C(7)-C(8)-Br(2)	113.0(3)
C(9)-C(8)-Br(2)	107.6(4)
C(7)-C(8)-H(8)	107.3
C(9)-C(8)-H(8)	107.3
Br(2)-C(8)-H(8)	107.3
O(2)-C(9)-C(8)	107.8(4)
O(2)-C(9)-H(9A)	110.1
C(8)-C(9)-H(9A)	110.1
O(2)-C(9)-H(9B)	110.1
C(8)-C(9)-H(9B)	110.1
H(9A)-C(9)-H(9B)	108.5

Symmetry transformations used to generate equivalent atoms:

Table 4. Anisotropic displacement parameters ($Å^2 x10^3$) for **2a**. The anisotropic displacement factor exponent takes the form: -2 ^{2}[h^2 $a^{*2}U^{11}$ + ... + 2 h k a* b* U^{12}].

	U^{11}	U^{22}	U^{33}	U^{23}	U^{13}	U^{12}
Br(1)	50(1)	29(1)	34(1)	7(1)	-9(1)	6(1)
Br(2)	30(1)	30(1)	32(1)	-11(1)	11(1)	-3(1)
O(1)	17(2)	24(2)	27(2)	-6(2)	0(1)	0(2)
O(2)	22(2)	28(2)	33(2)	9(2)	10(2)	6(2)
C(1)	16(2)	19(3)	22(3)	-3(2)	2(2)	-4(2)
C(2)	22(3)	21(3)	27(3)	-1(2)	5(2)	-2(2)
C(3)	32(3)	29(3)	24(3)	-2(2)	6(2)	-6(2)
C(4)	29(3)	18(3)	25(3)	2(2)	-9(2)	-2(2)
C(5)	18(2)	24(3)	33(3)	0(2)	1(2)	0(2)
C(6)	21(3)	19(3)	31(3)	1(2)	7(2)	-2(2)
C(7)	18(2)	23(3)	20(2)	-1(2)	6(2)	-1(2)
C(8)	18(2)	19(2)	23(3)	-1(2)	5(2)	1(2)
C(9)	20(2)	24(3)	27(3)	2(2)	4(2)	-4(2)

Table 5. Hydrogen coordinates (x10[4]) and isotropic displacement parameters (Å^{2}x10^3) for **2a**.

	x	y	z	U(eq)
H(1)	8195	4647	9641	35
H(2)	210	2106	9273	40
H(2A)	6610	4959	6931	28
H(3)	4852	6920	5574	34
H(5)	-281	7319	7430	31
H(6)	1565	5403	8822	28
H(7)	4820	3839	9688	24
H(8)	5565	1132	9246	24
H(9A)	1438	2190	7683	29
H(9B)	2012	298	7991	29

Table 6. Torsion angles [°] for **2a**.

C(6)-C(1)-C(2)-C(3)	-0.6(8)
C(7)-C(1)-C(2)-C(3)	179.3(5)
C(1)-C(2)-C(3)-C(4)	-1.2(8)
C(2)-C(3)-C(4)-C(5)	2.0(8)
C(2)-C(3)-C(4)-Br(1)	-177.5(4)
C(3)-C(4)-C(5)-C(6)	-1.0(8)
Br(1)-C(4)-C(5)-C(6)	178.5(4)
C(4)-C(5)-C(6)-C(1)	-0.9(8)
C(2)-C(1)-C(6)-C(5)	1.6(8)
C(7)-C(1)-C(6)-C(5)	-178.2(5)
C(6)-C(1)-C(7)-O(1)	-140.4(5)
C(2)-C(1)-C(7)-O(1)	39.7(7)
C(6)-C(1)-C(7)-C(8)	95.9(6)
C(2)-C(1)-C(7)-C(8)	-84.0(6)
O(1)-C(7)-C(8)-C(9)	176.0(4)
C(1)-C(7)-C(8)-C(9)	-57.6(6)
O(1)-C(7)-C(8)-Br(2)	-60.6(4)
C(1)-C(7)-C(8)-Br(2)	65.8(5)
C(7)-C(8)-C(9)-O(2)	-65.1(6)
Br(2)-C(8)-C(9)-O(2)	168.7(4)

Symmetry transformations used to generate equivalent atoms:

Table 7. Hydrogen bonds for **2a** [Å and °].

D-H...A	d(D-H)	d(H...A)	d(D...A)	<(DHA)
O(1)-H(1)...O(2)#1	0.82	1.89	2.699(6)	171.7
O(2)-H(2)...O(1)#2	0.82	1.94	2.736(5)	162.7

Symmetry transformations used to generate equivalent atoms:

#1 -x+1,y+1/2,-z+2 #2 x-1,y,z

The X-ray structure of compound **2e**

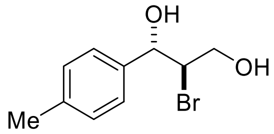

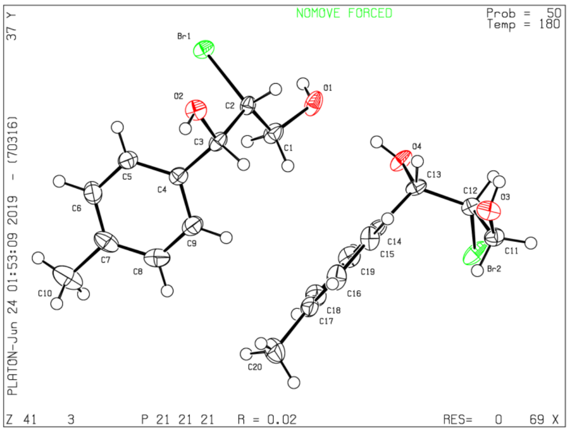

Table 1. Crystal data and structure refinement for **2e**.

Identification code	**2e**	
Empirical formula	$C_{10}H_{13}BrO_2$	
Formula weight	245.11	
Temperature	180.00(10) K	
Wavelength	0.71073 Å	
Crystal system	Orthorhombic	
Space group	$P2_1$	
Unit cell dimensions	a = 8.30480(10) Å	α= 90°.
	b = 9.2603(2) Å	β= 90°.
	c = 27.0823(7) Å	γ = 90°.
Volume	2082.76(7) $Å^3$	
Z	8	
Density (calculated)	1.563 Mg/m^3	
Absorption coefficient	3.913 mm^{-1}	
F(000)	992	
Crystal size	0.42 x 0.08 x 0.04 mm^3	
Theta range for data collection	2.324 to 27.484°.	
Index ranges	-10<=h<=10, -12<=k<=12, -35<=l<=35	
Reflections collected	48490	
Independent reflections	4767 [R(int) = 0.0444]	
Completeness to theta = 25.242°	100.0 %	
Absorption correction	Semi-empirical from equivalents	
Max. and min. transmission	1.00000 and 0.75718	
Refinement method	Full-matrix least-squares on F^2	
Data / restraints / parameters	4767 / 4 / 249	
Goodness-of-fit on F^2	1.013	
Final R indices [I>2sigma(I)]	R1 = 0.0229, wR2 = 0.0499	
R indices (all data)	R1 = 0.0257, wR2 = 0.0507	
Absolute structure parameter	-0.006(3)	
Extinction coefficient	n/a	
Largest diff. peak and hole	0.277 and -0.335 e.$Å^{-3}$	

Table 2. Atomic coordinates (x10[4]) and equivalent isotropic displacement parameters ($Å^2$x10[3]) for **2e**. U(eq) is defined as one third of the trace of the orthogonalized U^{ij} tensor.

	x	y	z	U(eq)
Br(1)	-600(1)	1450(1)	4592(1)	24(1)
O(1)	1555(2)	4480(2)	4524(1)	31(1)
O(2)	2170(3)	-656(2)	4349(1)	25(1)
C(1)	1735(4)	3347(3)	4173(1)	28(1)
C(2)	1638(3)	1867(3)	4407(1)	19(1)
C(3)	2352(3)	671(3)	4085(1)	22(1)
C(4)	1700(3)	586(3)	3566(1)	22(1)
C(5)	325(4)	-198(3)	3449(1)	27(1)
C(6)	-250(4)	-230(4)	2967(1)	33(1)
C(7)	506(4)	496(4)	2590(1)	34(1)
C(8)	1884(4)	1274(4)	2707(1)	35(1)
C(9)	2474(4)	1326(4)	3184(1)	30(1)
C(10)	-108(5)	426(5)	2064(1)	49(1)
Br(2)	6180(1)	8809(1)	3907(1)	33(1)
O(3)	8608(3)	5726(2)	4723(1)	28(1)
O(4)	3708(2)	6716(2)	4387(1)	27(1)
C(11)	8247(3)	6911(3)	4405(1)	24(1)
C(12)	6479(3)	7335(3)	4427(1)	22(1)
C(13)	5285(3)	6104(3)	4364(1)	21(1)
C(14)	5546(3)	5176(3)	3911(1)	20(1)
C(15)	6551(4)	3975(3)	3939(1)	27(1)
C(16)	6871(4)	3142(3)	3525(1)	31(1)
C(17)	6183(4)	3478(3)	3074(1)	29(1)
C(18)	5144(4)	4644(3)	3052(1)	31(1)
C(19)	4826(4)	5491(3)	3461(1)	28(1)
C(20)	6562(5)	2614(4)	2615(1)	43(1)

Table 3. Bond lengths [Å] and angles [°] for **2e**.

Br(1)-C(2)	1.963(3)
O(1)-C(1)	1.424(4)
O(1)-H(1)	0.80(2)
O(2)-C(3)	1.431(3)
O(2)-H(2)	0.79(2)
C(1)-C(2)	1.512(4)
C(1)-H(1A)	0.9700
C(1)-H(1B)	0.9700
C(2)-C(3)	1.530(4)
C(2)-H(2A)	0.9800
C(3)-C(4)	1.507(4)
C(3)-H(3A)	0.9800
C(4)-C(5)	1.390(4)
C(4)-C(9)	1.397(4)
C(5)-C(6)	1.391(4)
C(5)-H(5)	0.9300
C(6)-C(7)	1.375(5)
C(6)-H(6)	0.9300
C(7)-C(8)	1.388(5)
C(7)-C(10)	1.514(5)
C(8)-C(9)	1.383(4)
C(8)-H(8)	0.9300
C(9)-H(9)	0.9300
C(10)-H(10A)	0.9600
C(10)-H(10B)	0.9600
C(10)-H(10C)	0.9600
Br(2)-C(12)	1.976(3)
O(3)-C(11)	1.427(4)
O(3)-H(3)	0.79(2)
O(4)-C(13)	1.429(3)
O(4)-H(4)	0.82(2)
C(11)-C(12)	1.521(4)
C(11)-H(11A)	0.9700
C(11)-H(11B)	0.9700
C(12)-C(13)	1.520(4)
C(12)-H(12)	0.9800

C(13)-C(14)	1.514(4)
C(13)-H(13)	0.9800
C(14)-C(19)	1.389(4)
C(14)-C(15)	1.392(4)
C(15)-C(16)	1.386(4)
C(15)-H(15)	0.9300
C(16)-C(17)	1.386(5)
C(16)-H(16)	0.9300
C(17)-C(18)	1.384(4)
C(17)-C(20)	1.511(4)
C(18)-C(19)	1.384(4)
C(18)-H(18)	0.9300
C(19)-H(19)	0.9300
C(20)-H(20A)	0.9600
C(20)-H(20B)	0.9600
C(20)-H(20C)	0.9600
C(1)-O(1)-H(1)	113(3)
C(3)-O(2)-H(2)	109(3)
O(1)-C(1)-C(2)	112.5(3)
O(1)-C(1)-H(1A)	109.1
C(2)-C(1)-H(1A)	109.1
O(1)-C(1)-H(1B)	109.1
C(2)-C(1)-H(1B)	109.1
H(1A)-C(1)-H(1B)	107.8
C(1)-C(2)-C(3)	113.3(2)
C(1)-C(2)-Br(1)	109.58(19)
C(3)-C(2)-Br(1)	111.75(19)
C(1)-C(2)-H(2A)	107.3
C(3)-C(2)-H(2A)	107.3
Br(1)-C(2)-H(2A)	107.3
O(2)-C(3)-C(4)	112.6(2)
O(2)-C(3)-C(2)	107.1(2)
C(4)-C(3)-C(2)	115.5(2)
O(2)-C(3)-H(3A)	107.1
C(4)-C(3)-H(3A)	107.1
C(2)-C(3)-H(3A)	107.1
C(5)-C(4)-C(9)	117.8(3)
C(5)-C(4)-C(3)	122.3(3)

C(9)-C(4)-C(3)	119.9(3)
C(4)-C(5)-C(6)	120.5(3)
C(4)-C(5)-H(5)	119.8
C(6)-C(5)-H(5)	119.8
C(7)-C(6)-C(5)	122.0(3)
C(7)-C(6)-H(6)	119.0
C(5)-C(6)-H(6)	119.0
C(6)-C(7)-C(8)	117.4(3)
C(6)-C(7)-C(10)	121.6(3)
C(8)-C(7)-C(10)	121.0(3)
C(9)-C(8)-C(7)	121.6(3)
C(9)-C(8)-H(8)	119.2
C(7)-C(8)-H(8)	119.2
C(8)-C(9)-C(4)	120.8(3)
C(8)-C(9)-H(9)	119.6
C(4)-C(9)-H(9)	119.6
C(7)-C(10)-H(10A)	109.5
C(7)-C(10)-H(10B)	109.5
H(10A)-C(10)-H(10B)	109.5
C(7)-C(10)-H(10C)	109.5
H(10A)-C(10)-H(10C)	109.5
H(10B)-C(10)-H(10C)	109.5
C(11)-O(3)-H(3)	112(3)
C(13)-O(4)-H(4)	107(3)
O(3)-C(11)-C(12)	112.2(2)
O(3)-C(11)-H(11A)	109.2
C(12)-C(11)-H(11A)	109.2
O(3)-C(11)-H(11B)	109.2
C(12)-C(11)-H(11B)	109.2
H(11A)-C(11)-H(11B)	107.9
C(13)-C(12)-C(11)	115.6(2)
C(13)-C(12)-Br(2)	110.9(2)
C(11)-C(12)-Br(2)	105.8(2)
C(13)-C(12)-H(12)	108.1
C(11)-C(12)-H(12)	108.1
Br(2)-C(12)-H(12)	108.1
O(4)-C(13)-C(14)	113.0(2)
O(4)-C(13)-C(12)	107.2(2)

C(14)-C(13)-C(12)	115.0(2)
O(4)-C(13)-H(13)	107.1
C(14)-C(13)-H(13)	107.1
C(12)-C(13)-H(13)	107.1
C(19)-C(14)-C(15)	118.2(3)
C(19)-C(14)-C(13)	122.0(2)
C(15)-C(14)-C(13)	119.7(3)
C(16)-C(15)-C(14)	121.1(3)
C(16)-C(15)-H(15)	119.5
C(14)-C(15)-H(15)	119.5
C(17)-C(16)-C(15)	120.6(3)
C(17)-C(16)-H(16)	119.7
C(15)-C(16)-H(16)	119.7
C(18)-C(17)-C(16)	118.0(3)
C(18)-C(17)-C(20)	120.6(3)
C(16)-C(17)-C(20)	121.4(3)
C(17)-C(18)-C(19)	121.8(3)
C(17)-C(18)-H(18)	119.1
C(19)-C(18)-H(18)	119.1
C(18)-C(19)-C(14)	120.1(3)
C(18)-C(19)-H(19)	119.9
C(14)-C(19)-H(19)	119.9
C(17)-C(20)-H(20A)	109.5
C(17)-C(20)-H(20B)	109.5
H(20A)-C(20)-H(20B)	109.5
C(17)-C(20)-H(20C)	109.5
H(20A)-C(20)-H(20C)	109.5
H(20B)-C(20)-H(20C)	109.5

Symmetry transformations used to generate equivalent atoms:

Table 4. Anisotropic displacement parameters ($Å^2x10^3$) for **2e**. The anisotropic displacement factor exponent takes the form: -2 ^{2}[h^2 $a^{*2}U^{11}$ + ...+ 2 h k a* b* U^{12}].

	U^{11}	U^{22}	U^{33}	U^{23}	U^{13}	U^{12}
Br(1)	21(1)	21(1)	30(1)	3(1)	5(1)	3(1)
O(1)	19(1)	18(1)	55(2)	-8(1)	4(1)	-1(1)
O(2)	26(1)	18(1)	32(1)	0(1)	3(1)	8(1)
C(1)	25(2)	19(2)	39(2)	-2(1)	7(1)	-1(1)
C(2)	14(1)	18(1)	26(1)	-1(1)	-2(1)	0(1)
C(3)	16(1)	18(1)	32(2)	3(1)	2(1)	0(1)
C(4)	19(1)	18(1)	27(2)	-2(1)	4(1)	3(1)
C(5)	27(2)	27(2)	27(2)	-1(1)	5(1)	-3(1)
C(6)	29(2)	35(2)	34(2)	-10(1)	-2(1)	-3(1)
C(7)	37(2)	39(2)	26(2)	-7(1)	2(2)	13(2)
C(8)	38(2)	36(2)	31(2)	3(2)	12(1)	5(2)
C(9)	27(2)	27(2)	36(2)	0(1)	7(1)	-3(1)
C(10)	52(2)	67(3)	29(2)	-8(2)	0(2)	17(2)
Br(2)	23(1)	22(1)	54(1)	15(1)	4(1)	-1(1)
O(3)	28(1)	33(1)	25(1)	3(1)	1(1)	10(1)
O(4)	18(1)	16(1)	47(1)	3(1)	10(1)	2(1)
C(11)	18(1)	28(2)	26(2)	1(1)	2(1)	2(1)
C(12)	22(2)	20(1)	26(2)	2(1)	4(1)	1(1)
C(13)	18(1)	18(1)	28(1)	5(1)	7(1)	4(1)
C(14)	16(1)	17(1)	27(1)	2(1)	4(1)	1(1)
C(15)	26(2)	20(1)	35(2)	-1(1)	-7(1)	4(1)
C(16)	23(2)	24(2)	47(2)	-8(1)	-3(1)	5(1)
C(17)	28(1)	25(2)	34(2)	-7(1)	6(1)	-7(1)
C(18)	36(2)	28(2)	27(2)	4(1)	-4(1)	-2(1)
C(19)	28(2)	20(1)	34(2)	4(1)	2(1)	5(1)
C(20)	48(2)	37(2)	45(2)	-19(2)	5(2)	-5(2)

Table 5. Hydrogen coordinates (x10^4) and isotropic displacement parameters (Å^{2}x10^3) for **2e**.

	x	y	z	U(eq)
H(1)	640(30)	4670(40)	4579(14)	46
H(2)	2690(40)	-1260(30)	4215(12)	38
H(1A)	2767	3448	4009	33
H(1B)	899	3433	3925	33
H(2A)	2269	1899	4713	23
H(3A)	3510	859	4058	26
H(5)	-215	-705	3695	32
H(6)	-1175	-759	2898	39
H(8)	2422	1772	2459	42
H(9)	3397	1859	3252	36
H(10A)	569	-203	1874	74
H(10B)	-94	1376	1922	74
H(10C)	-1190	61	2063	74
H(3)	8310(50)	5860(40)	4998(9)	43
H(4)	3080(40)	6060(30)	4451(13)	41
H(11A)	8524	6653	4069	29
H(11B)	8902	7734	4499	29
H(12)	6277	7792	4747	27
H(13)	5402	5474	4653	25
H(15)	7015	3728	4240	32
H(16)	7555	2350	3551	37
H(18)	4646	4865	2754	37
H(19)	4128	6273	3435	33
H(20A)	7460	3043	2446	65
H(20B)	5641	2609	2400	65
H(20C)	6826	1640	2706	65

Table 6. Torsion angles [°] for **2e**.

O(1)-C(1)-C(2)-C(3)	-161.3(2)
O(1)-C(1)-C(2)-Br(1)	73.1(3)
C(1)-C(2)-C(3)-O(2)	-179.7(2)
Br(1)-C(2)-C(3)-O(2)	-55.3(3)
C(1)-C(2)-C(3)-C(4)	-53.4(3)
Br(1)-C(2)-C(3)-C(4)	71.0(3)
O(2)-C(3)-C(4)-C(5)	36.5(4)
C(2)-C(3)-C(4)-C(5)	-87.0(3)
O(2)-C(3)-C(4)-C(9)	-144.2(3)
C(2)-C(3)-C(4)-C(9)	92.4(3)
C(9)-C(4)-C(5)-C(6)	-0.3(4)
C(3)-C(4)-C(5)-C(6)	179.0(3)
C(4)-C(5)-C(6)-C(7)	0.2(5)
C(5)-C(6)-C(7)-C(8)	0.0(5)
C(5)-C(6)-C(7)-C(10)	178.6(3)
C(6)-C(7)-C(8)-C(9)	-0.3(5)
C(10)-C(7)-C(8)-C(9)	-178.8(3)
C(7)-C(8)-C(9)-C(4)	0.2(5)
C(5)-C(4)-C(9)-C(8)	0.0(5)
C(3)-C(4)-C(9)-C(8)	-179.3(3)
O(3)-C(11)-C(12)-C(13)	-50.1(4)
O(3)-C(11)-C(12)-Br(2)	-173.2(2)
C(11)-C(12)-C(13)-O(4)	-179.0(2)
Br(2)-C(12)-C(13)-O(4)	-58.6(2)
C(11)-C(12)-C(13)-C(14)	-52.4(4)
Br(2)-C(12)-C(13)-C(14)	68.0(3)
O(4)-C(13)-C(14)-C(19)	33.7(4)
C(12)-C(13)-C(14)-C(19)	-89.8(3)
O(4)-C(13)-C(14)-C(15)	-147.0(3)
C(12)-C(13)-C(14)-C(15)	89.4(3)
C(19)-C(14)-C(15)-C(16)	2.2(4)
C(13)-C(14)-C(15)-C(16)	-177.1(3)
C(14)-C(15)-C(16)-C(17)	-0.7(5)
C(15)-C(16)-C(17)-C(18)	-1.4(5)
C(15)-C(16)-C(17)-C(20)	177.9(3)
C(16)-C(17)-C(18)-C(19)	2.0(5)

C(20)-C(17)-C(18)-C(19)	-177.3(3)
C(17)-C(18)-C(19)-C(14)	-0.5(5)
C(15)-C(14)-C(19)-C(18)	-1.6(4)
C(13)-C(14)-C(19)-C(18)	177.6(3)

Symmetry transformations used to generate equivalent atoms:

Table 7. Hydrogen bonds for **2e** [Å and °].

D-H...A	d(D-H)	d(H...A)	d(D...A)	<(DHA)
O(1)-H(1)...O(3)#1	0.80(2)	1.99(2)	2.759(3)	163(4)
O(2)-H(2)...O(4)#2	0.79(2)	2.11(3)	2.750(3)	138(3)
O(3)-H(3)...O(2)#3	0.79(2)	2.01(2)	2.781(3)	163(4)
O(4)-H(4)...O(1)	0.82(2)	1.94(2)	2.760(3)	174(4)

Symmetry transformations used to generate equivalent atoms:

#1 x-1,y,z #2 x,y-1,z #3 x+1/2,-y+1/2,-z+1

The X-ray structure of compound **12**

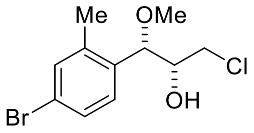

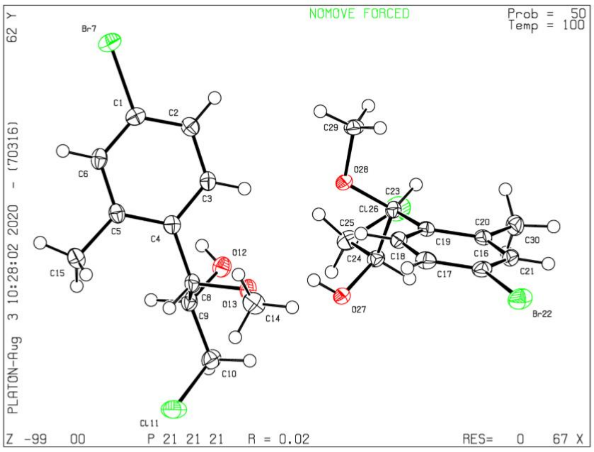

Table 1. Crystal data and structure refinement for **12**.

Identification code	**12**	
Empirical formula	$C_{11}H_{14}BrClO_2$	
Formula weight	293.59	
Temperature	99.99(10) K	
Wavelength	0.71073 Å	
Crystal system	orthorhombic	
Space group	$P2_1$	
Unit cell dimensions	a = 10.45924(4) Å	α= 90°.
	b = 10.51707(4) Å	β= 90°.
	c = 22.10012(8) Å	γ = 90°.
Volume	2431.027(16) $Å^3$	
Z	4	
Density (calculated)	1.604 Mg/m^3	
Absorption coefficient	6.465 mm^{-1}	
F(000)	1184.0	
Crystal size	0.2 x 0.15 x 0.1 mm^3	
Theta range for data collection	8.002 to 151.056°.	
Index ranges	-13<=h<=12, -13<=k<=12, -27<=l<=27	
Reflections collected	58808	
Independent reflections	4930 [R(int) = 0.0345]	
Completeness to theta = 25.242°	100 %	
Absorption correction	Semi-empirical from equivalents	
Max. and min. transmission	1.00000 and 0.75235	
Refinement method	Full-matrix least-squares on F^2	
Data / restraints / parameters	4930 / 0 / 277	
Goodness-of-fit on F2	1.080	
Final R indices [I>2sigma(I)]	R1 = 0.0174, wR2 = 0.0432	
R indices (all data)	R1 = 0.0174, wR2 = 0.0432	
Absolute structure parameter	0.001(3)	
Extinction coefficient	n/a	
Largest diff. peak and hole	0.33 and -0.51 e.$Å^{-3}$	

Table 2. Fractional atomic coordinates (x10^4) and equivalent isotropic displacement parameters (Å^{2}x10^3) for **12**. U(eq) is defined as one third of the trace of the orthogonalized U^{ij} tensor.

	x	y	z	U(eq)
Br(7)	9565.1(3)	8009.0(2)	7853.5(2)	26.28(7)
Br(22)	9170.2(3)	-2819.2(3)	5709.3(2)	30.95(7)
Cl(26)	3507.3(6)	3543.1(7)	5686.5(3)	33.89(15)
Cl(11)	4645.8(7)	1423.1(6)	9157.2(3)	32.70(14)
O(28)	6939.1(15)	3148.7(16)	6345.8(7)	18.3(3)
O(27)	5255.0(16)	1469.4(16)	6938.3(7)	19.0(3)
O(13)	6785.2(15)	2083.2(16)	7908.9(7)	19.9(3)
O(12)	4793.9(17)	3893.9(17)	7805.4(8)	22.4(3)
C(24)	5001(2)	1983(2)	6355.6(10)	18.4(4)
C(19)	6985(2)	1025(2)	5899.0(10)	16.6(5)
C(8)	6605(2)	3029(2)	8363.7(10)	18.4(5)
C(21)	7390(2)	-909(2)	5351.9(11)	21.7(5)
C(17)	8583(2)	-455(2)	6265.4(11)	21.9(5)
C(4)	7416(2)	4200(2)	8250.9(11)	17.5(5)
C(2)	8269(2)	5726(2)	7531.4(11)	20.3(5)
C(20)	6719(2)	226(2)	5405.1(10)	19.6(5)
C(18)	7919(2)	678(2)	6318.1(11)	19.7(5)
C(16)	8299(2)	-1237(2)	5778.9(11)	21.6(5)
C(5)	7898(2)	4929(2)	8731.7(11)	18.7(5)
C(3)	7616(2)	4607(2)	7658.1(11)	19.3(5)
C(23)	6233(2)	2244(2)	6001.1(10)	17.3(4)
C(1)	8709(2)	6446(2)	8011.1(11)	19.1(5)
C(9)	5170(2)	3361(2)	8367.2(11)	19.0(5)
C(30)	5732(2)	566(3)	4935.1(11)	24.2(5)
C(29)	7891(2)	3776(2)	5993.1(11)	21.9(5)
C(6)	8544(2)	6056(2)	8604.5(11)	19.8(5)
C(25)	4167(2)	3162(2)	6413.4(12)	24.2(5)
C(14)	7780(3)	1214(3)	8044.5(12)	26.5(6)
C(10)	4315(2)	2212(2)	8454.9(11)	24.0(5)
C(15)	7776(3)	4519(3)	9384.4(11)	26.4(5)

Table 3. Bond lengths [Å] and angles [°] for **12**.

Br(7)-C(1)	1.904(2)
Br(22)-C(16)	1.904(2)
Cl(26)-C(25)	1.793(3)
Cl(11)-C(10)	1.793(3)
O(28)-C(23)	1.425(3)
O(28)-C(29)	1.426(3)
O(27)-C(24)	1.421(3)
O(13)-C(8)	1.426(3)
O(13)-C(14)	1.417(3)
O(12)-C(19)	1.418(3)
C(24)-C(23)	1.533(3)
C(24)-C(25)	1.522(4)
C(19)-C(20)	1.406(3)
C(19)-C(18)	1.395(3)
C(19)-C(23)	1.520(3)
C(8)-C(4)	1.516(3)
C(8)-C(9)	1.541(3)
C(21)-C(20)	1.390(4)
C(21)-C(16)	1.383(4)
C(17)-C(18)	1.385(4)
C(17)-C(16)	1.386(4)
C(4)-C(5)	1.404(3)
C(4)-C(3)	1.394(3)
C(2)-C(3)	1.390(4)
C(2)-C(1)	1.381(3)
C(20)-C(30)	1.507(3)
C(5)-C(6)	1.393(4)
C(5)-C(15)	1.511(3)
C(1)-C(6)	1.385(3)
C(9)-C(10)	1.516(3)
C(23)-O(28)-C(29)	112.24(18)
C(14)-O(13)-C(8)	113.45(18)
O(27)-C(24)-C(23)	111.96(18)
O(27)-C(24)-C(25)	109.91(19)
C(25)-C(24)-C(23)	112.3(2)
C(20)-C(19)-C(23)	121.1(2)

C(18)-C(19)-C(20)	119.8(2)
C(18)-C(19)-C(23)	119.0(2)
O(13)-C(8)-C(4)	112.10(18)
O(13)-C(8)-C(9)	106.91(18)
C(4)-C(8)-C(9)	111.2(2)
C(16)-C(21)-C(20)	120.2(2)
C(18)-C(17)-C(16)	117.9(2)
C(5)-C(4)-C(8)	121.3(2)
C(3)-C(4)-C(8)	119.2(2)
C(3)-C(4)-C(5)	119.3(2)
C(1)-C(2)-C(3)	118.2(2)
C(19)-C(20)-C(30)	121.9(2)
C(21)-C(20)-C(19)	118.7(2)
C(21)-C(20)-C(30)	119.4(2)
C(17)-C(18)-C(19)	121.4(2)
C(21)-C(16)-Br(22)	119.46(19)
C(21)-C(16)-C(17)	121.9(2)
C(17)-C(16)-Br(22)	118.60(19)
C(4)-C(5)-C(15)	122.4(2)
C(6)-C(5)-C(4)	119.1(2)
C(6)-C(5)-C(15)	118.5(2)
C(2)-C(3)-C(4)	121.6(2)
O(28)-C(23)-C(24)	106.38(18)
O(28)-C(23)-C(19)	111.98(18)
C(19)-C(23)-C(24)	111.1(2)
C(2)-C(1)-Br(7)	119.28(18)
C(2)-C(1)-C(6)	121.5(2)
C(6)-C(1)-Br(7)	119.17(18)
O(12)-C(9)-C(8)	110.83(19)
O(12)-C(9)-C(10)	105.26(19)
C(10)-C(9)-C(8)	113.2(2)
C(1)-C(6)-C(5)	120.2(2)
C(24)-C(25)-Cl(26)	109.11
C(9)-C(10)-Cl(11)	111.43(17)

Symmetry transformations used to generate equivalent atoms:

Table 4. Anisotropic displacement parameters ($Å^2x10^3$) for **12**. The anisotropic displacement factor exponent takes the form: -2 ^{2}[h^2 $a^{*2}U^{11}$ + ...+ 2 h k a* b* U^{12}].

	U^{11}	U^{22}	U^{33}	U^{23}	U^{13}	U^{12}
Br(7)	24.20(12)	21.43(13)	33.21(13)	2.67(11)	-2.97(10)	-5.06(10)
Br(22)	31.24(14)	20.80(13)	40.81(15)	-0.30(12)	9.42(12)	3.41(11)
Cl(26)	28.0(3)	33.2(3)	40.4(3)	6.8(3)	-13.3(3)	2.7(3)
Cl(11)	34.6(3)	26.4(3)	37.1(3)	6.6(3)	9.0(3)	-3.9(3)
O(28)	18.0(8)	18.5(8)	18.4(7)	-0.6(7)	-0.6(6)	-6.3(7)
O(27)	20.2(8)	18.8(8)	18.0(7)	0.1(6)	-0.6(6)	-4.0(7)
O(13)	21.1(8)	17.0(8)	21.5(7)	-4.7(7)	1.0(6)	5.2(7)
O(12)	22.1(9)	21.5(8)	23.5(8)	-2.0(7)	-2.3(7)	1.5(7)
C(24)	17.0(10)	18.6(11)	19.7(10)	0.1(9)	-2.4(8)	-3.2(9)
C(19)	15.9(11)	18.0(11)	15.9(10)	0.7(9)	2.3(8)	-2.8(9)
C(8)	19.1(11)	19.1(12)	17.0(10)	-4.2(9)	1.4(8)	1.4(10)
C(21)	23.5(12)	20.9(12)	20.6(12)	-4.8(9)	5.9(10)	-7.2(10)
C(17)	18.8(12)	23.7(13)	23.1(12)	4.8(10)	-0.9(9)	-1.1(10)
C(4)	14.1(10)	19.1(12)	19.2(11)	-1.6(9)	0.8(9)	3.4(9)
C(2)	18.6(11)	23.4(12)	18.8(11)	0.9(9)	-0.8(9)	1.2(10)
C(20)	17.6(11)	23.8(12)	17.4(11)	0.1(9)	2.8(9)	-7.4(10)
C(18)	20.9(12)	21.0(12)	17.2(11)	0.2(9)	-0.9(9)	-4.8(10)
C(16)	21.0(11)	17.8(11)	26.0(12)	0.1(10)	8.1(10)	-1.5(9)
C(5)	16.2(11)	22.8(12)	16.9(11)	-2.9(9)	1.0(9)	2.7(9)
C(3)	19.4(11)	21.4(12)	17.2(11)	-3.0(9)	-0.8(9)	0.1(10)
C(23)	18.7(10)	17.7(11)	15.4(9)	-0.3(9)	-2.7(8)	-2.1(9)
C(1)	13.2(10)	18.3(12)	25.9(12)	0.4(9)	0.8(9)	1.5(9)
C(9)	18.5(11)	19.2(12)	19.3(10)	-4.1(9)	1.7(9)	1.3(9)
C(30)	21.3(12)	31.1(14)	20.4(11)	-3.6(10)	-1.8(10)	-6.2(10)
C(29)	21.6(12)	21.6(13)	22.5(12)	3.5(10)	0.2(10)	-7.1(10)
C(6)	15.6(11)	22.9(12)	20.9(11)	-6.1(9)	-1.9(9)	1.7(9)
C(25)	19.2(11)	23.1(13)	30.1(12)	1.1(10)	-2.7(10)	-0.8(10)
C(14)	27.5(14)	23.4(13)	28.6(13)	2.7(11)	3.6(11)	10.5(11)
C(10)	22.2(12)	21.6(12)	28.3(12)	-4.8(10)	6.0(9)	-1.1(11)
C(15)	30.8(14)	31.0(14)	17.5(11)	-1.8(10)	1.3(10)	-4.2(11)

Table 5. Hydrogen coordinates (x10^4) and isotropic displacement parameters (Å^{2}x10^3) for **12**.

	x	y	z	U(eq)
H(27)	5680	1978	7135	28
H(12)	4716	4666	7842	34
H(24)	4512	1346	6129	22
H(8)	6835	2667	8758	22
H(21)	7227	-1450	5028	26
H(17)	9202	-684	6548	26
H(2)	8405	5985	7134	24
H(18)	8099	1219	6640	24
H(3)	7306	4117	7340	23
H(23)	6013	2617	5608	21
H(9)	5003	3977	8691	23
H(30A)	4893	489	5107	36
H(30B)	5809	0	4596	36
H(30C)	5865	1425	4803	36
H(29A)	7486	4267	5681	33
H(29B)	8445	3154	5813	33
H(29C)	8383	4329	6248	33
H(6)	8866	6548	8919	24
H(25A)	4674	3869	6561	29
H(25B)	3483	3006	6700	29
H(14A)	7845	596	7726	40
H(14B)	7594	790	8419	40
H(14C)	8574	1665	8080	40
H(10A)	4444	1621	8123	29
H(10B)	3427	2479	8449	29
H(15A)	6898	4325	9472	40
H(15B)	8060	5194	9644	40
H(15C)	8291	3777	9452	40

Table 6. Torsion angles [°] for **12**.

Br(7)-C(1)-C(6)-C(5)	178.70(18)
O(27)-C(24)-C(23)-O(28)	-60.9(2)
O(27)-C(24)-C(23)-C(19)	61.2(2)
O(27)-C(24)-C(25)-Cl(26)	-164.65(15)
O(13)-C(8)-C(4)-C(5)	-147.9(2)
O(13)-C(8)-C(4)-C(3)	36.3(3)
O(13)-C(8)-C(9)-O(12)	-62.8(2)
O(13)-C(8)-C(9)-C(10)	55.2(2)
O(12)-C(9)-C(10)-Cl(11)	-179.18(15)
C(8)-C(4)-C(5)-C(6)	-174.5(2)
C(8)-C(4)-C(5)-C(15)	7.2(4)
C(8)-C(4)-C(3)-C(2)	175.0(2)
C(8)-C(9)-C(10)-Cl(11)	59.6(2)
C(4)-C(8)-C(9)-O(12)	59.8(2)
C(4)-C(8)-C(9)-C(10)	177.82(19)
C(4)-C(5)-C(6)-C(1)	-0.2(4)
C(2)-C(1)-C(6)-C(5)	-1.4(4)
C(20)-C(19)-C(18)-C(17)	-1.1(4)
C(20)-C(19)-C(23)-O(28)	-155.7(2)
C(20)-C(19)-C(23)-C(24)	85.5 (3)
C(20)-C(21)-C(16)-Br(22)	178.19(17)
C(20)-C(21)-C(16)-C(17)	-0.7(4)
C(18)-C(19)-C(20)-C(21)	0.8(3)
C(18)-C(19)-C(20)-C(30)	-179.2(2)
C(18)-C(19)-C(23)-O(28)	26.8(3)
C(18)-C(19)-C(23)-C(24)	-92.0(2)
C(18)-C(17)-C(16)-Br(22)	-178.40(18)
C(18)-C(17)-C(16)-C(21)	0.5(4)
C(16)-C(21)-C(20)-C(19)	0.0(3)
C(16)-C(21)-C(20)-C(30)	180.0(2)
C(16)-C(17)-C(18)-C(19)	0.4(4)
C(5)-C(4)-C(3)-C(2)	-0.8(4)
C(3)-C(4)-C(5)-C(6)	1.3(4)
C(3)-C(4)-C(5)-C(15)	-177.0(2)
C(3)-C(2)-C(1)-Br(7)	-178.28(18)
C(3)-C(2)-C(1)-C(6)	1.8(4)

C(23)-C(24)-C(25)-Cl(26)	70.0(2)
C(23)-C(19)-C(20)-C(21)	-176.6(2)
C(23)-C(19)-C(20)-C(30)	3.4(3)
C(23)-C(19)-C(18)-C(17)	176.5(2)
C(1)-C(2)-C(3)-C(4)	-0.7(4)
C(9)-C(8)-C(4)-C(5)	92.5(3)
C(9)-C(8)-C(4)-C(3)	-83.3(3)
C(29)-O(28)-C(23)-C(24)	-161.54(19)
C(29)-O(28)-C(23)-C(19)	76.9(2)
C(25)-C(24)-C(23)-O(28)	63.3(2)
C(25)-C(24)-C(23)-C(19)	-174.59(19)
C(14)-O(13)-C(8)-C(4)	89.9(2)
C(14)-O(13)-C(8)-C(9)	-148.0(2)
C(15)-C(5)-C(6)-C(1)	178.2(2)

Symmetry transformations used to generate equivalent atoms:

Table 7. Hydrogen bonds for **12** [Å and °].

D-H...A	d(D-H)	d(H...A)	d(D...A)	<(DHA)

The determination of enantiomeric excess

Table 2, entry 1

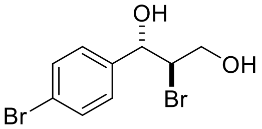

2a

HPLC Condition: Column: Chiralpak AS-H, Daicel Chemical Industries, Ltd.; **Eluent:** Hexanes/IPA (90/10); **Flow rate:** 1.0 mL/min; **Detection:** UV203 nm.

Racemic standard

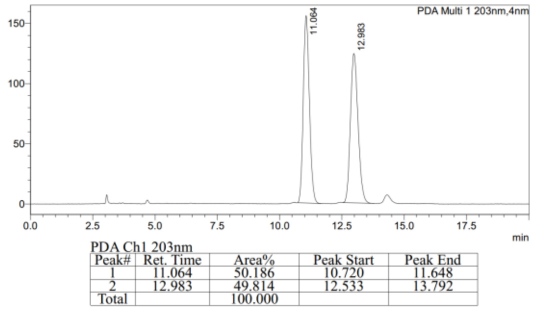

PDA Ch1 203nm

Peak#	Ret. Time	Area%	Peak Start	Peak End
1	11.064	50.186	10.720	11.648
2	12.983	49.814	12.533	13.792
Total		100.000		

Enantio-enriched product

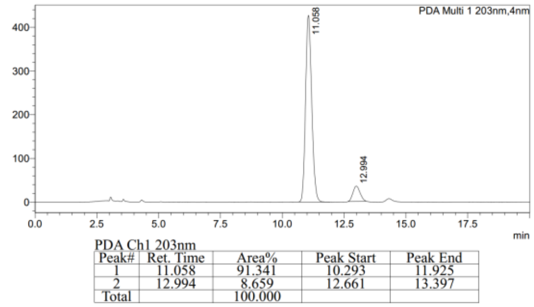

PDA Ch1 203nm

Peak#	Ret. Time	Area%	Peak Start	Peak End
1	11.058	91.341	10.293	11.925
2	12.994	8.659	12.661	13.397
Total		100.000		

Table 2, entry 2

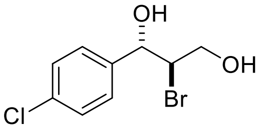

2b

HPLC Condition: Column: Chiralpak AS-H, Daicel Chemical Industries, Ltd.; **Eluent:** Hexanes/IPA (90/10); **Flow rate:** 1.0 mL/min; **Detection:** UV202 nm.

Racemic standard

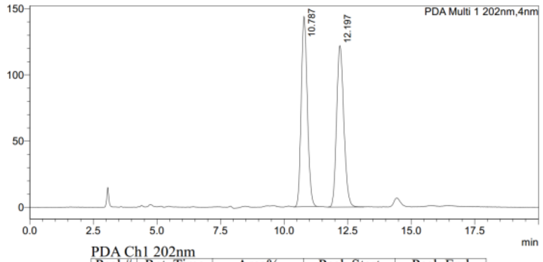

PDA Ch1 202nm

Peak#	Ret. Time	Area%	Peak Start	Peak End
1	10.787	50.029	10.389	11.392
2	12.197	49.971	11.733	13.141
Total		100.000		

Enantio-enriched product

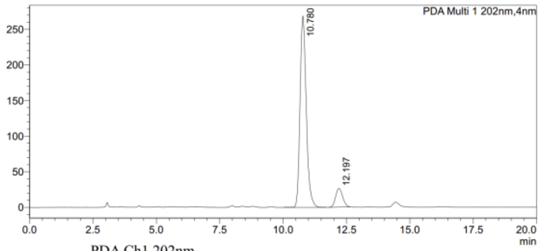

PDA Ch1 202nm

Peak#	Ret. Time	Area%	Peak Start	Peak End
1	10.780	90.089	10.027	11.691
2	12.197	9.911	11.808	12.640
Total		100.000		

Table 2, entry 3

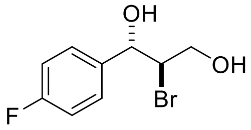

2c

HPLC Condition: Column: Chiralpak AD-H, Daicel Chemical Industries, Ltd.; **Eluent:** Hexanes/IPA (90/10); **Flow rate:** 1.0 mL/min; **Detection:** UV208 nm.

Racemic standard

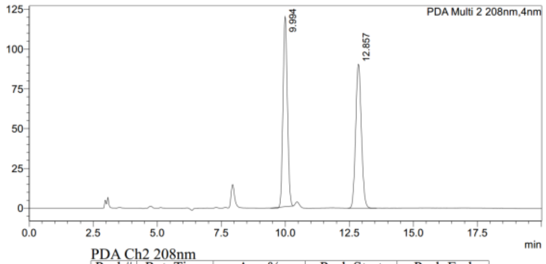

PDA Ch2 208nm

Peak#	Ret. Time	Area%	Peak Start	Peak End
1	9.994	50.047	9.408	10.304
2	12.857	49.953	12.437	13.568
Total		100.000		

Enantio-enriched product

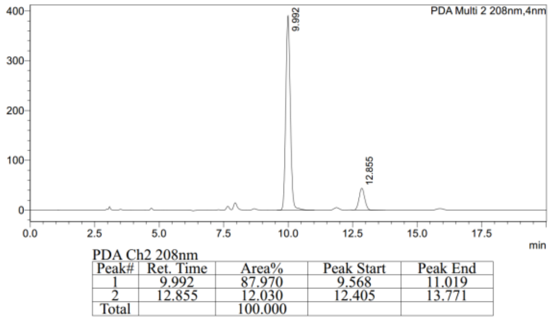

PDA Ch2 208nm

Peak#	Ret. Time	Area%	Peak Start	Peak End
1	9.992	87.970	9.568	11.019
2	12.855	12.030	12.405	13.771
Total		100.000		

Table 2, entry 4

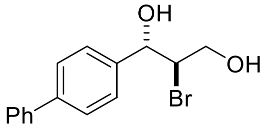

2d

HPLC Condition: Column: Chiralpak AS-H, Daicel Chemical Industries, Ltd.; **Eluent:** Hexanes/IPA (90/10); **Flow rate:** 1.0 mL/min; **Detection:** UV207 nm.

Racemic standard

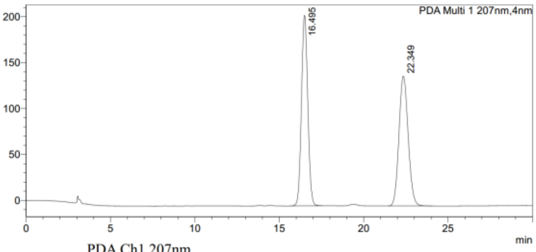

PDA Ch1 207nm

Peak#	Ret. Time	Area%	Peak Start	Peak End
1	16.495	50.045	15.659	17.525
2	22.349	49.955	21.440	24.053
Total		100.000		

Enantio-enriched product

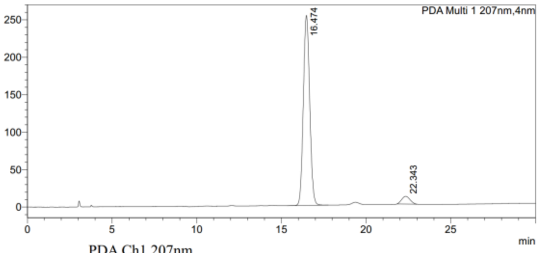

PDA Ch1 207nm

Peak#	Ret. Time	Area%	Peak Start	Peak End
1	16.474	94.947	15.349	17.771
2	22.343	5.053	21.824	22.997
Total		100.000		

Table 2, entry 5

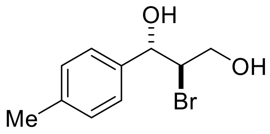

2e

HPLC Condition: Column: Chiralpak AS-H, Daicel Chemical Industries, Ltd.; **Eluent:** Hexanes/IPA (90/10); **Flow rate:** 1.0 mL/min; **Detection:** UV203 nm.

Racemic standard

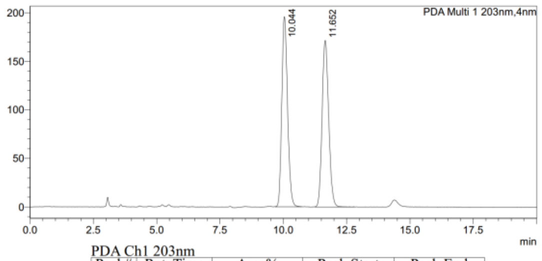

PDA Ch1 203nm

Peak#	Ret. Time	Area%	Peak Start	Peak End
1	10.044	49.926	9.675	10.720
2	11.652	50.074	11.232	12.811
Total		100.000		

Enantio-enriched product

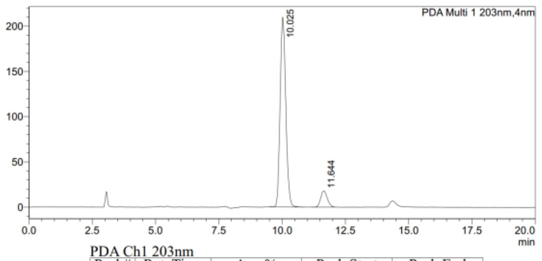

PDA Ch1 203nm

Peak#	Ret. Time	Area%	Peak Start	Peak End
1	10.025	90.893	9.493	10.880
2	11.644	9.107	11.168	12.107
Total		100.000		

Table 2, entry 6

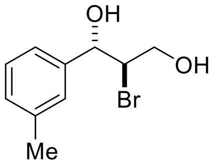

2f

HPLC Condition: Column: Chiralpak AS-H, Daicel Chemical Industries, Ltd.; **Eluent:** Hexanes/IPA (90/10); **Flow rate:** 1.0 mL/min; **Detection:** UV195 nm.

Racemic standard

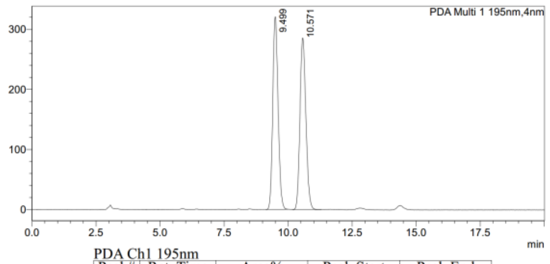

PDA Ch1 195nm

Peak#	Ret. Time	Area%	Peak Start	Peak End
1	9.499	50.166	9.131	9.984
2	10.571	49.834	10.165	11.296
Total		100.000		

Enantio-enriched product

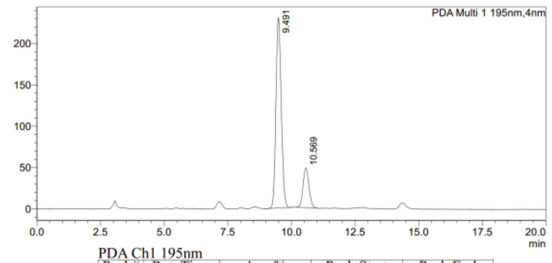

PDA Ch1 195nm

Peak#	Ret. Time	Area%	Peak Start	Peak End
1	9.491	81.178	8.949	10.069
2	10.569	18.822	10.187	11.008
Total		100.000		

Table 2, entry 7

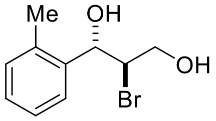

2g

HPLC Condition: Column: Chiralpak AS-H, Daicel Chemical Industries, Ltd.; **Eluent:** Hexanes/IPA (90/10); **Flow rate:** 1.0 mL/min; **Detection:** UV195 nm.

Racemic standard

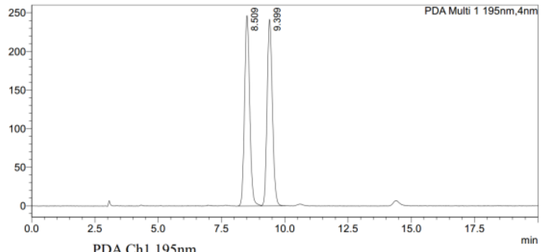

PDA Ch1 195nm

Peak#	Ret. Time	Area%	Peak Start	Peak End
1	8.509	50.163	8.139	9.077
2	9.399	49.837	9.077	10.027
Total		100.000		

Enantio-enriched product

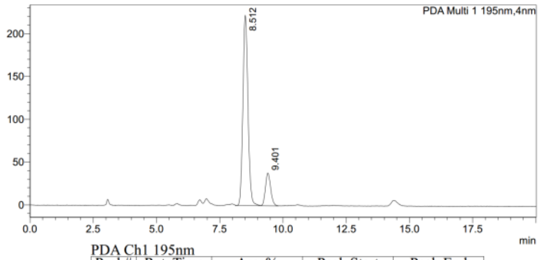

PDA Ch1 195nm

Peak#	Ret. Time	Area%	Peak Start	Peak End
1	8.512	84.916	8.117	9.088
2	9.401	15.084	9.088	9.824
Total		100.000		

Table 2, entry 8

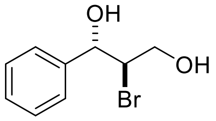

2h

HPLC Condition: Column: Chiralpak AS-H, Daicel Chemical Industries, Ltd.; **Eluent:** Hexanes/IPA (90/10); **Flow rate:** 1.0 mL/min; **Detection:** UV194 nm.

Racemic standard

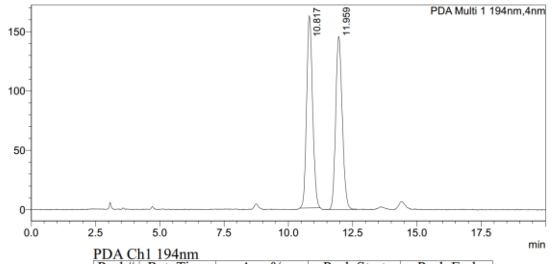

PDA Ch1 194nm

Peak#	Ret. Time	Area%	Peak Start	Peak End
1	10.817	50.188	10.443	11.232
2	11.959	49.812	11.413	12.693
Total		100.000		

Enantio-enriched product

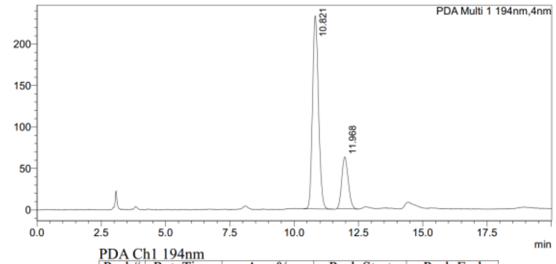

PDA Ch1 194nm

Peak#	Ret. Time	Area%	Peak Start	Peak End
1	10.821	77.602	10.357	11.499
2	11.968	22.398	11.659	12.512
Total		100.000		

Table 2, entry 9

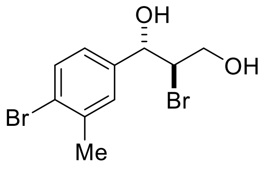

2i

HPLC Condition: Column: Chiralpak AD-H, Daicel Chemical Industries, Ltd.; **Eluent:** Hexanes/IPA (90/10); **Flow rate:** 1.0 mL/min; **Detection:** UV205 nm.

Racemic standard

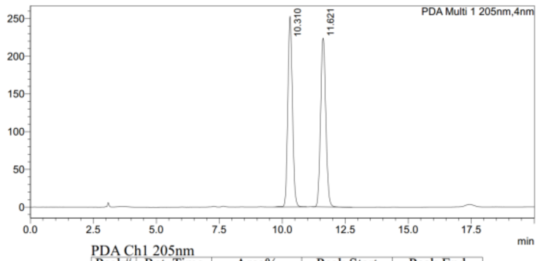

PDA Ch1 205nm

Peak#	Ret. Time	Area%	Peak Start	Peak End
1	10.310	49.930	9.653	10.976
2	11.621	50.070	11.168	12.768
Total		100.000		

Enantio-enriched product

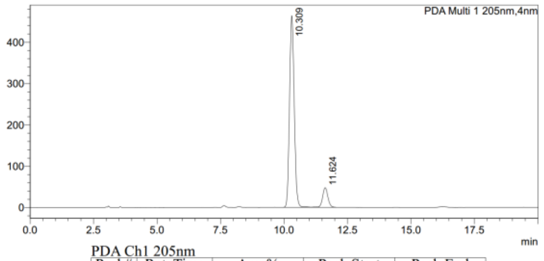

PDA Ch1 205nm

Peak#	Ret. Time	Area%	Peak Start	Peak End
1	10.309	89.822	9.963	11.296
2	11.624	10.178	11.296	12.064
Total		100.000		

Table 2, entry 10

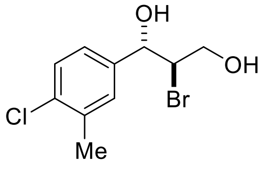

2j

HPLC Condition: Column: Chiralpak AD-H, Daicel Chemical Industries, Ltd.; **Eluent:** Hexanes/IPA (90/10); **Flow rate:** 1.0 mL/min; **Detection:** UV204 nm.

Racemic standard

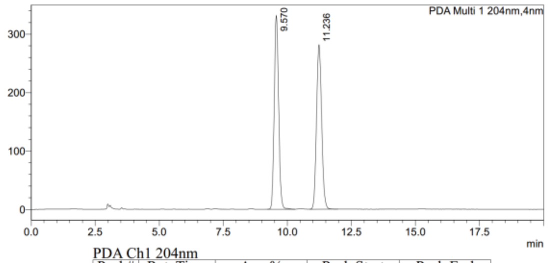

PDA Ch1 204nm

Peak#	Ret. Time	Area%	Peak Start	Peak End
1	9.570	49.961	9.227	10.293
2	11.236	50.039	10.869	11.979
Total		100.000		

Enantio-enriched product

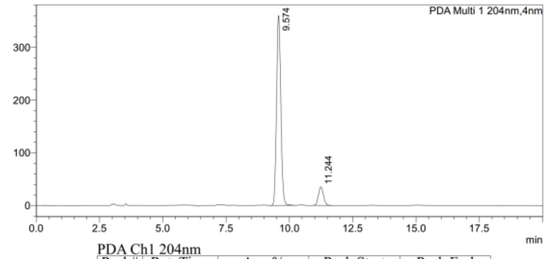

PDA Ch1 204nm

Peak#	Ret. Time	Area%	Peak Start	Peak End
1	9.574	89.838	9.248	10.197
2	11.244	10.162	10.965	11.701
Total		100.000		

Table 2, entry 11

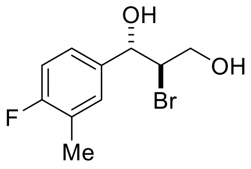

2k

HPLC Condition: Column: Chiralpak AD-H, Daicel Chemical Industries, Ltd.; **Eluent:** Hexanes/IPA (90/10); **Flow rate:** 1.0 mL/min; **Detection:** UV191 nm.

Racemic standard

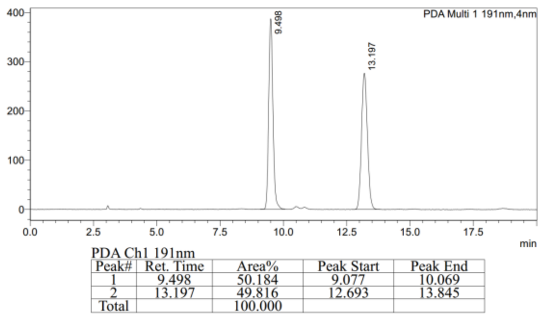

PDA Ch1 191nm

Peak#	Ret. Time	Area%	Peak Start	Peak End
1	9.498	50.184	9.077	10.069
2	13.197	49.816	12.693	13.845
Total		100.000		

Enantio-enriched product

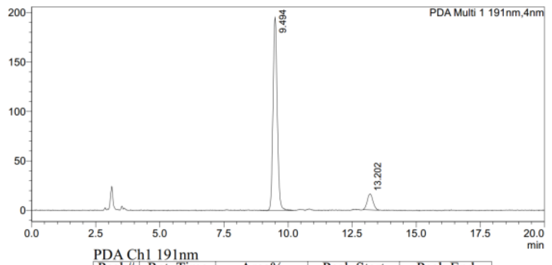

PDA Ch1 191nm

Peak#	Ret. Time	Area%	Peak Start	Peak End
1	9.494	89.855	8.960	10.208
2	13.202	10.145	12.928	13.557
Total		100.000		

Table 2, entry 12

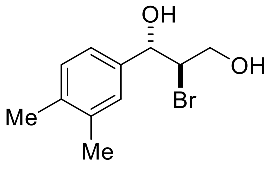

2l

HPLC Condition: Column: Chiralpak AD-H, Daicel Chemical Industries, Ltd.; **Eluent:** Hexanes/IPA (90/10); **Flow rate:** 1.0 mL/min; **Detection:** UV204 nm.

Racemic standard

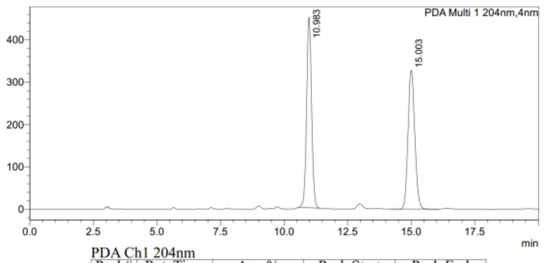

PDA Ch1 204nm

Peak#	Ret. Time	Area%	Peak Start	Peak End
1	10.983	50.057	10.560	11.445
2	15.003	49.943	14.219	16.085
Total		100.000		

Enantio-enriched product

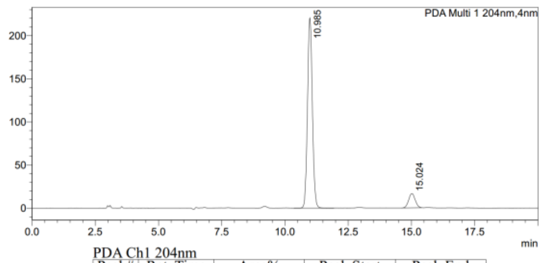

PDA Ch1 204nm

Peak#	Ret. Time	Area%	Peak Start	Peak End
1	10.985	90.856	10.357	11.936
2	15.024	9.144	14.603	15.403
Total		100.000		

Table 2, entry 13

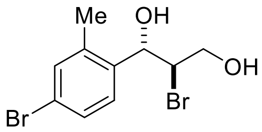

2m

HPLC Condition: Column: Chiralpak AD-H, Daicel Chemical Industries, Ltd.; **Eluent:** Hexanes/IPA (90/10); **Flow rate:** 1.0 mL/min; **Detection:** UV205 nm.

Racemic standard | **Enantio-enriched product**

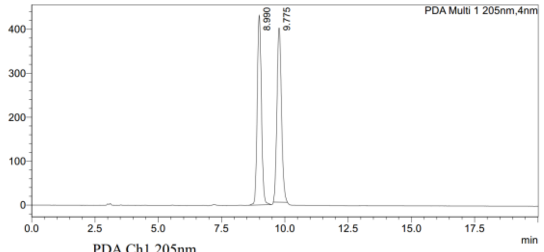

PDA Ch1 205nm

Peak#	Ret. Time	Area%	Peak Start	Peak End
1	8.990	49.906	8.576	9.461
2	9.775	50.094	9.547	10.101
Total		100.000		

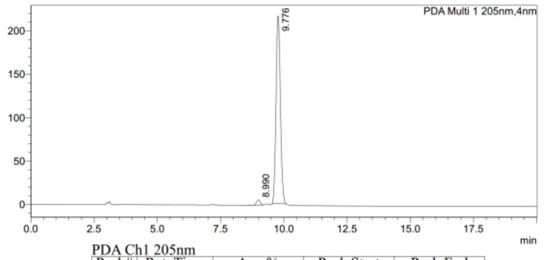

PDA Ch1 205nm

Peak#	Ret. Time	Area%	Peak Start	Peak End
1	8.990	2.293	8.576	9.195
2	9.776	97.707	9.547	10.091
Total		100.000		

Table 2, entry 14

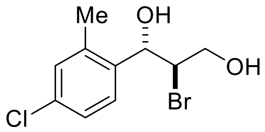

2n

HPLC Condition: Column: Chiralpak AS-H, Daicel Chemical Industries, Ltd.; **Eluent:** Hexanes/IPA (90/10); **Flow rate:** 1.0 mL/min; **Detection:** UV204 nm.

Racemic standard | **Enantio-enriched product**

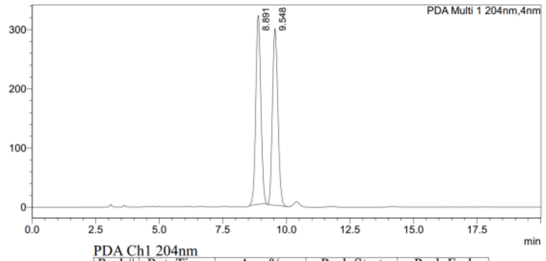

PDA Ch1 204nm

Peak#	Ret. Time	Area%	Peak Start	Peak End
1	8.891	50.216	8.544	9.205
2	9.548	49.784	9.269	10.027
Total		100.000		

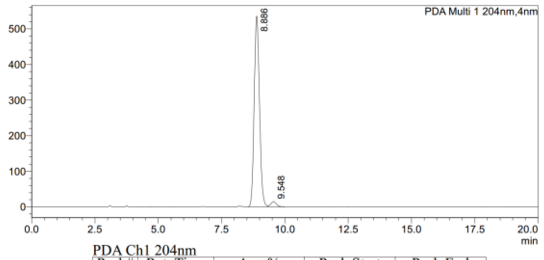

PDA Ch1 204nm

Peak#	Ret. Time	Area%	Peak Start	Peak End
1	8.886	97.215	8.533	9.333
2	9.548	2.785	9.333	9.995
Total		100.000		

Table 2, entry 15

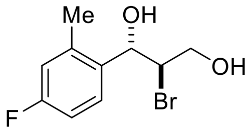

2o

HPLC Condition: **Column:** Chiralpak OD-H, Daicel Chemical Industries, Ltd.; **Eluent:** Hexanes/IPA (90/10); **Flow rate:** 1.0 mL/min; **Detection:** UV193 nm.

Racemic standard | **Enantio-enriched product**

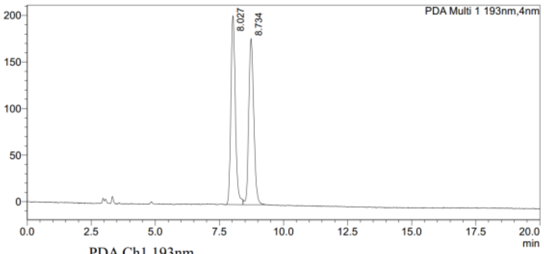

PDA Ch1 193nm

Peak#	Ret. Time	Area%	Peak Start	Peak End
1	8.027	50.236	7.776	8.416
2	8.734	49.764	8.416	9.312
Total		100.000		

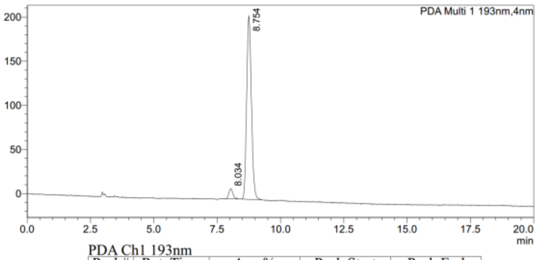

PDA Ch1 193nm

Peak#	Ret. Time	Area%	Peak Start	Peak End
1	8.034	4.526	7.861	8.331
2	8.754	95.474	8.480	9.227
Total		100.000		

Table 2, entry 16

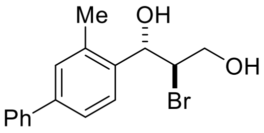

2p

HPLC Condition: **Column:** Chiralpak AS-H, Daicel Chemical Industries, Ltd.; **Eluent:** Hexanes/IPA (90/10); **Flow rate:** 1.0 mL/min; **Detection:** UV207 nm.

Racemic standard | **Enantio-enriched product**

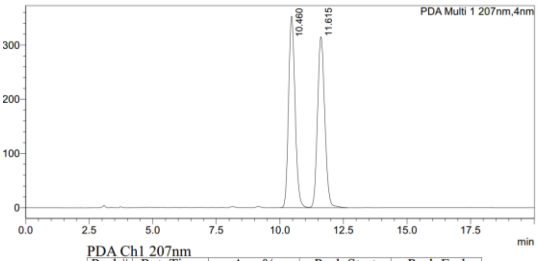

PDA Ch1 207nm

Peak#	Ret. Time	Area%	Peak Start	Peak End
1	10.460	50.146	10.005	11.157
2	11.615	49.854	11.157	12.661
Total		100.000		

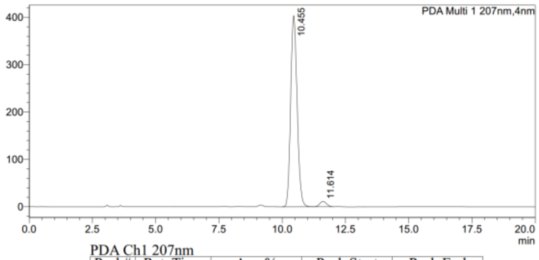

PDA Ch1 207nm

Peak#	Ret. Time	Area%	Peak Start	Peak End
1	10.455	97.158	10.005	11.232
2	11.614	2.842	11.232	12.021
Total		100.000		

Table 2, entry 17

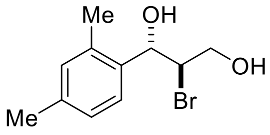

2q

HPLC Condition: Column: Chiralpak AS-H, Daicel Chemical Industries, Ltd.; **Eluent:** Hexanes/IPA (95/5); **Flow rate:** 1.0 mL/min; **Detection:** UV204 nm.

Racemic standard **Enantio-enriched product**

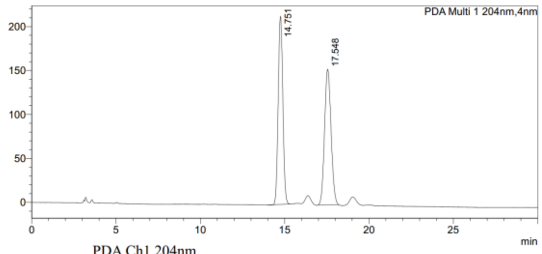

PDA Ch1 204nm

Peak#	Ret. Time	Area%	Peak Start	Peak End
1	14.751	49.844	13.984	15.317
2	17.548	50.156	16.981	18.197
Total		100.000		

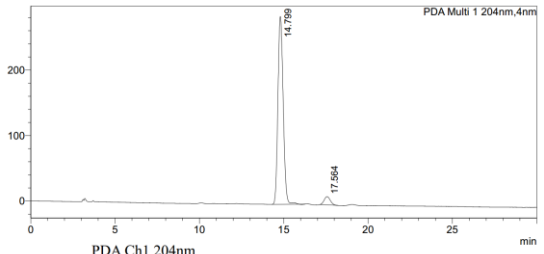

PDA Ch1 204nm

Peak#	Ret. Time	Area%	Peak Start	Peak End
1	14.799	95.064	14.293	16.256
2	17.564	4.936	17.152	18.197
Total		100.000		

Table 2, entry 18

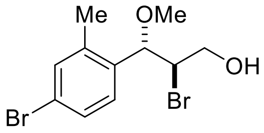

2r

HPLC Condition: Column: Chiralpak AS-H, Daicel Chemical Industries, Ltd.; **Eluent:** Hexanes/IPA (90/10); **Flow rate:** 1.0 mL/min; **Detection:** UV205 nm.

Racemic standard **Enantio-enriched product**

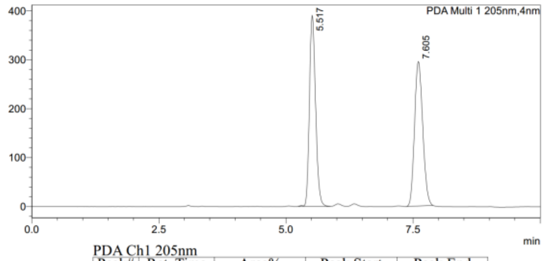

PDA Ch1 205nm

Peak#	Ret. Time	Area%	Peak Start	Peak End
1	5.517	49.840	5.237	5.877
2	7.605	50.160	7.360	7.893
Total		100.000		

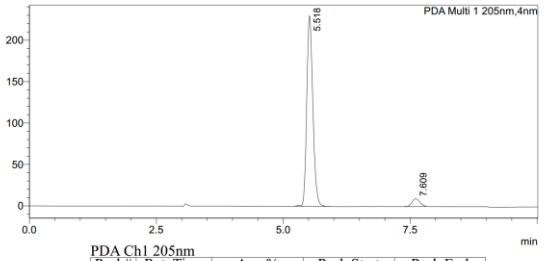

PDA Ch1 205nm

Peak#	Ret. Time	Area%	Peak Start	Peak End
1	5.518	95.098	5.227	5.931
2	7.609	4.902	7.381	7.829
Total		100.000		

Table 2, entry 19

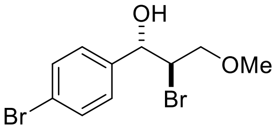

2s

HPLC Condition: Column: Chiralpak AS-H, Daicel Chemical Industries, Ltd.; **Eluent:** Hexanes/IPA (90/10); **Flow rate:** 1.0 mL/min; **Detection:** UV203 nm.

Racemic standard

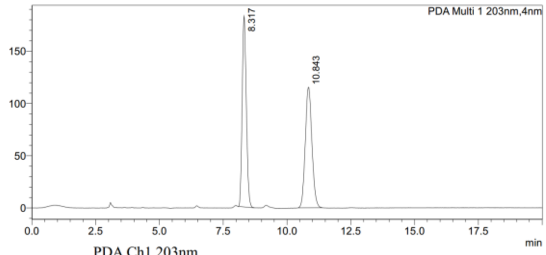

PDA Ch1 203nm

Peak#	Ret. Time	Area%	Peak Start	Peak End
1	8.317	49.807	8.075	8.725
2	10.843	50.193	10.443	11.371
Total		100.000		

Enantio-enriched product

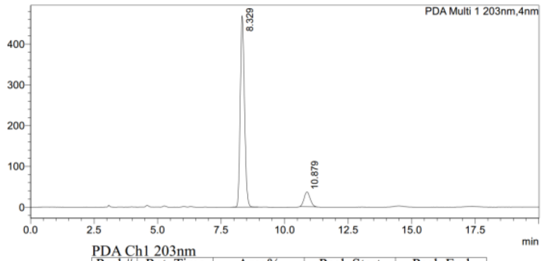

PDA Ch1 203nm

Peak#	Ret. Time	Area%	Peak Start	Peak End
1	8.329	89.965	7.872	8.960
2	10.879	10.035	10.603	11.285
Total		100.000		

Scheme 3, epoxide 4

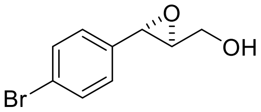

HPLC Condition: Column: Chiralpak AS-H, Daicel Chemical Industries, Ltd.; **Eluent:** Hexanes/IPA (90/10); **Flow rate:** 1.0 mL/min; **Detection:** UV203 nm.

Racemic standard

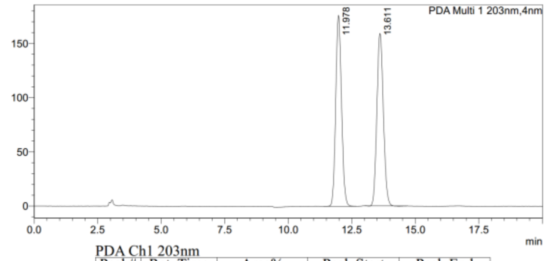

PDA Ch1 203nm

Peak#	Ret. Time	Area%	Peak Start	Peak End
1	11.978	49.867	11.381	12.683
2	13.611	50.133	13.003	14.709
Total		100.000		

Enantio-enriched product

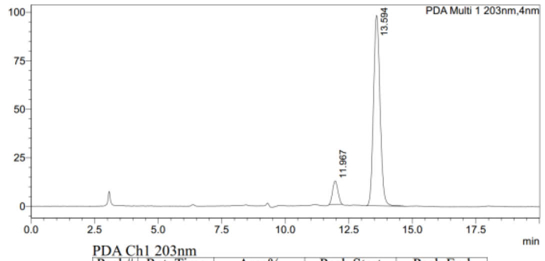

PDA Ch1 203nm

Peak#	Ret. Time	Area%	Peak Start	Peak End
1	11.967	9.141	11.723	12.245
2	13.594	90.859	13.184	14.752
Total		100.000		

Scheme 5, acetal 5

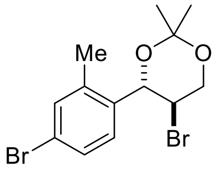

HPLC Condition: Column: Chiralpak AD-H, Daicel Chemical Industries, Ltd.; **Eluent:** Hexanes/IPA (95/5); **Flow rate:** 1.0 mL/min; **Detection:** UV204 nm.

Racemic standard

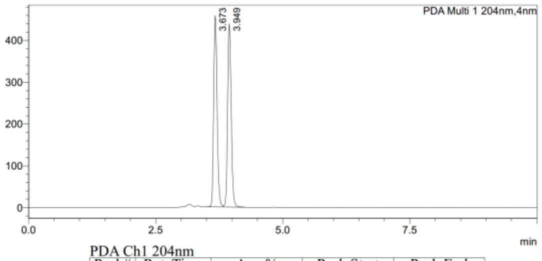

PDA Ch1 204nm

Peak#	Ret. Time	Area%	Peak Start	Peak End
1	3.673	49.926	3.499	3.829
2	3.949	50.074	3.829	4.277
Total		100.000		

Enantio-enriched product

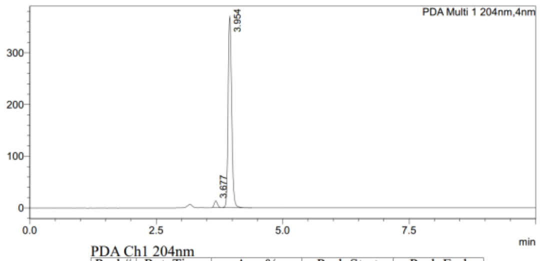

PDA Ch1 204nm

Peak#	Ret. Time	Area%	Peak Start	Peak End
1	3.677	2.996	3.605	3.776
2	3.954	97.004	3.808	4.395
Total		100.000		

Scheme 5, sulfide 6

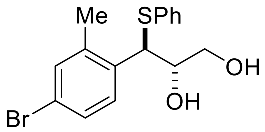

HPLC Condition: Column: Chiralpak AD-H, Daicel Chemical Industries, Ltd.; **Eluent:** Hexanes/IPA (90/10); **Flow rate:** 1.0 mL/min; **Detection:** UV190 nm.

Racemic standard

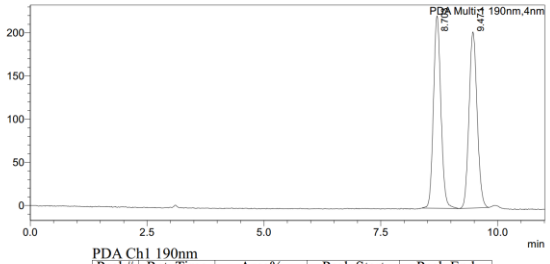

PDA Ch1 190nm

Peak#	Ret. Time	Area%	Peak Start	Peak End
1	8.703	50.086	8.384	9.163
2	9.471	49.914	9.163	9.803
Total		100.000		

Enantio-enriched product

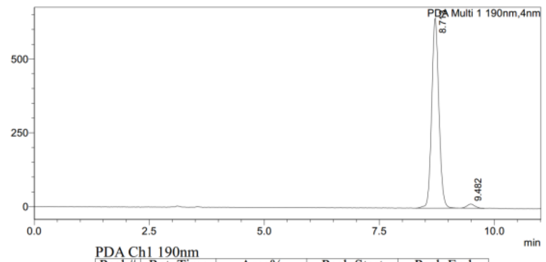

PDA Ch1 190nm

Peak#	Ret. Time	Area%	Peak Start	Peak End
1	8.713	97.680	8.288	9.216
2	9.482	2.320	9.269	9.781
Total		100.000		

Scheme 6, azide 7

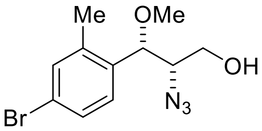

HPLC Condition: Column: Chiralpak AS-H, Daicel Chemical Industries, Ltd.; **Eluent:** Hexanes/IPA (90/10); **Flow rate:** 1.0 mL/min; **Detection:** UV205 nm.

Racemic standard

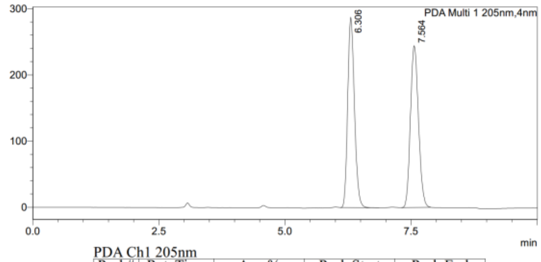

PDA Ch1 205nm

Peak#	Ret. Time	Area%	Peak Start	Peak End
1	6.306	49.828	6.101	6.869
2	7.564	50.172	7.307	7.893
Total		100.000		

Enantio-enriched product

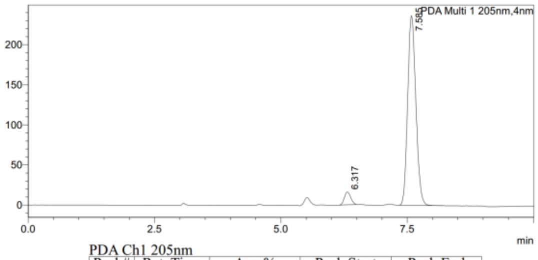

PDA Ch1 205nm

Peak#	Ret. Time	Area%	Peak Start	Peak End
1	6.317	4.985	6.165	6.507
2	7.585	95.015	7.339	8.203
Total		100.000		

Scheme 6, chloride 8

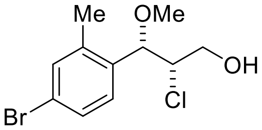

HPLC Condition: Column: Chiralpak AS-H, Daicel Chemical Industries, Ltd.; **Eluent:** Hexanes/IPA (98/2); **Flow rate:** 1.0 mL/min; **Detection:** UV204 nm.

Racemic standard

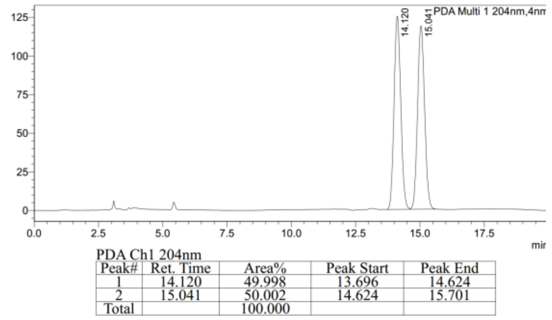

PDA Ch1 204nm

Peak#	Ret. Time	Area%	Peak Start	Peak End
1	14.120	49.998	13.696	14.624
2	15.041	50.002	14.624	15.701
Total		100.000		

Enantio-enriched product

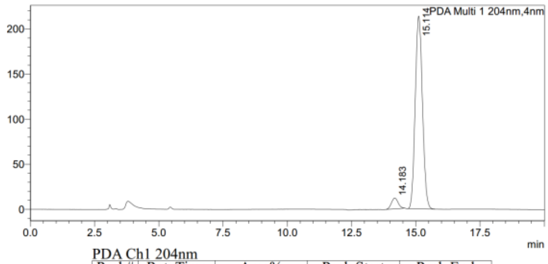

PDA Ch1 204nm

Peak#	Ret. Time	Area%	Peak Start	Peak End
1	14.183	4.958	13.856	14.581
2	15.114	95.042	14.699	15.744
Total		100.000		

Scheme 6, epoxide 9

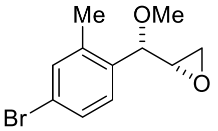

HPLC Condition: Column: Chiralpak AS-H, Daicel Chemical Industries, Ltd.; **Eluent:** Hexanes/IPA (90/10); **Flow rate:** 1.0 mL/min; **Detection:** UV205 nm.

Racemic standard

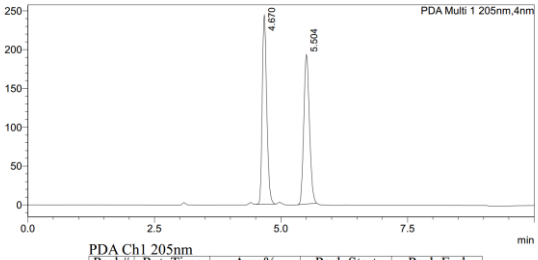

PDA Ch1 205nm

Peak#	Ret. Time	Area%	Peak Start	Peak End
1	4.670	50.054	4.512	4.896
2	5.504	49.946	5.333	5.707
Total		100.000		

Enantio-enriched product

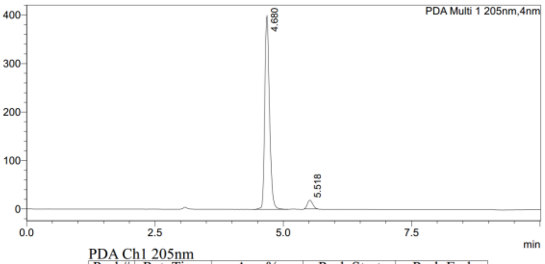

PDA Ch1 205nm

Peak#	Ret. Time	Area%	Peak Start	Peak End
1	4.680	95.129	4.416	5.099
2	5.518	4.871	5.408	5.675
Total		100.000		

Scheme 6, sulfide 10

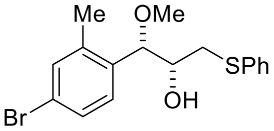

HPLC Condition: Column: Chiralpak AS-H, Daicel Chemical Industries, Ltd.; **Eluent:** Hexanes/IPA (90/10); **Flow rate:** 1.0 mL/min; **Detection:** UV205 nm.

Racemic standard

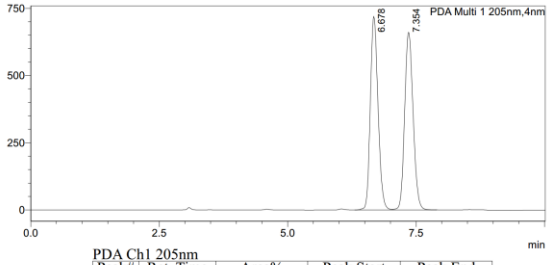

PDA Ch1 205nm

Peak#	Ret. Time	Area%	Peak Start	Peak End
1	6.678	49.823	6.304	7.040
2	7.354	50.177	7.040	7.904
Total		100.000		

Enantio-enriched product

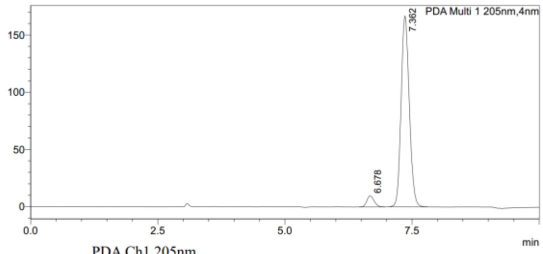

PDA Ch1 205nm

Peak#	Ret. Time	Area%	Peak Start	Peak End
1	6.678	4.883	6.464	6.965
2	7.362	95.117	7.029	7.808
Total		100.000		

Scheme 7, azide 11

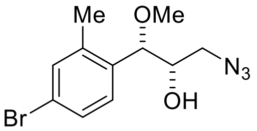

HPLC Condition: Column: Chiralpak OD-H, Daicel Chemical Industries, Ltd.; **Eluent:** Hexanes/IPA (90/10); **Flow rate:** 1.0 mL/min; **Detection:** UV205 nm.

Racemic standard

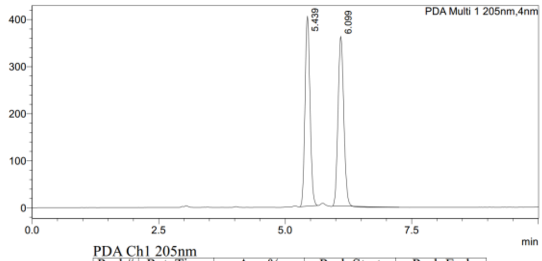

PDA Ch1 205nm

Peak#	Ret. Time	Area%	Peak Start	Peak End
1	5.439	49.841	5.291	5.653
2	6.099	50.159	5.941	7.253
Total		100.000		

Enantio-enriched product

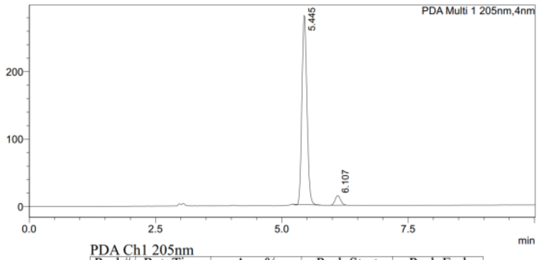

PDA Ch1 205nm

Peak#	Ret. Time	Area%	Peak Start	Peak End
1	5.445	94.823	5.205	5.771
2	6.107	5.177	5.973	6.272
Total		100.000		

Scheme 7, chloride 12

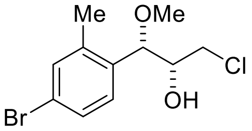

HPLC Condition: Column: Chiralpak OD-H, Daicel Chemical Industries, Ltd.; **Eluent:** Hexanes/IPA (95/5); **Flow rate:** 1.0 mL/min; **Detection:** UV222 nm.

Racemic standard

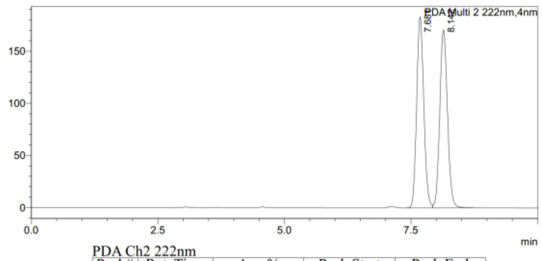

PDA Ch2 222nm

Peak#	Ret. Time	Area%	Peak Start	Peak End
1	7.681	49.960	7.424	7.925
2	8.142	50.040	7.925	8.736
Total		100.000		

Enantio-enriched product

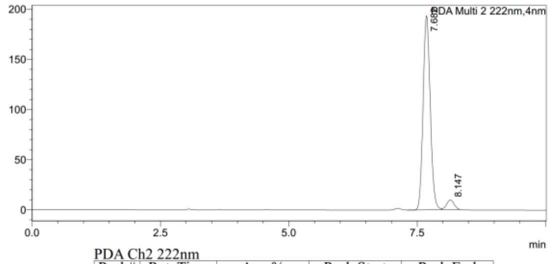

PDA Ch2 222nm

Peak#	Ret. Time	Area%	Peak Start	Peak End
1	7.682	95.051	7.307	7.979
2	8.147	4.949	7.979	8.341
Total		100.000		

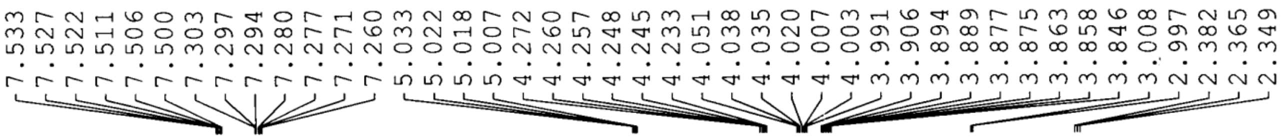

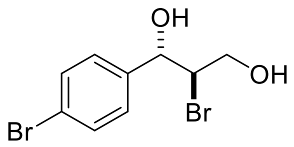

Table 2, Entry 1, **2a**

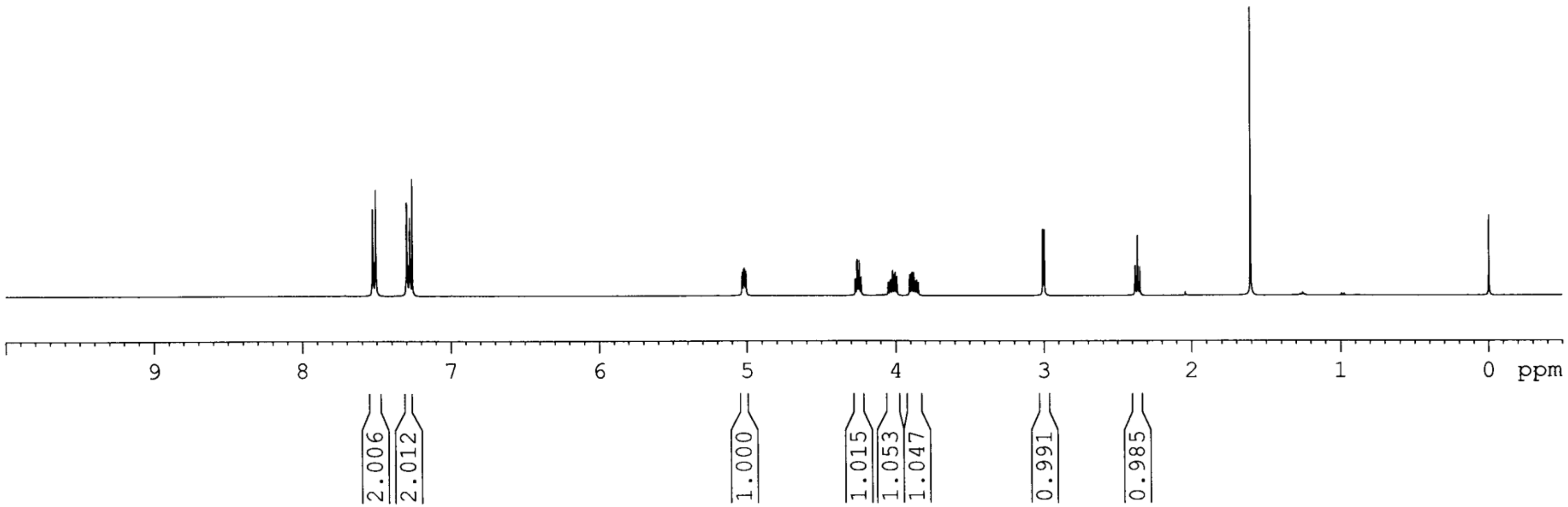

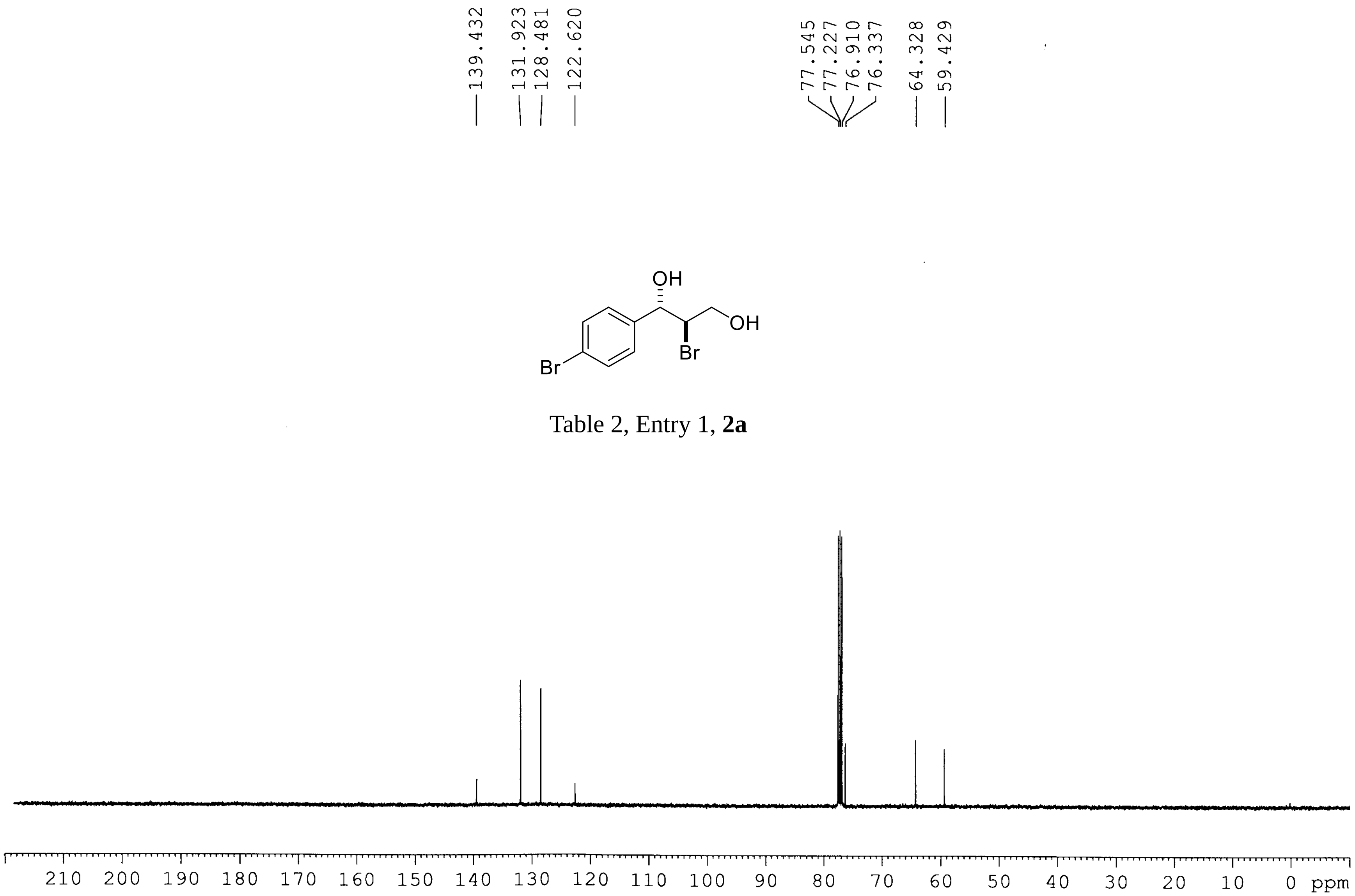

Table 2, Entry 1, **2a**

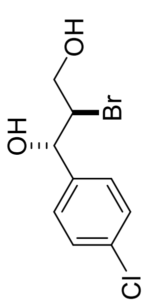

Table 2, Entry 2, **2b**

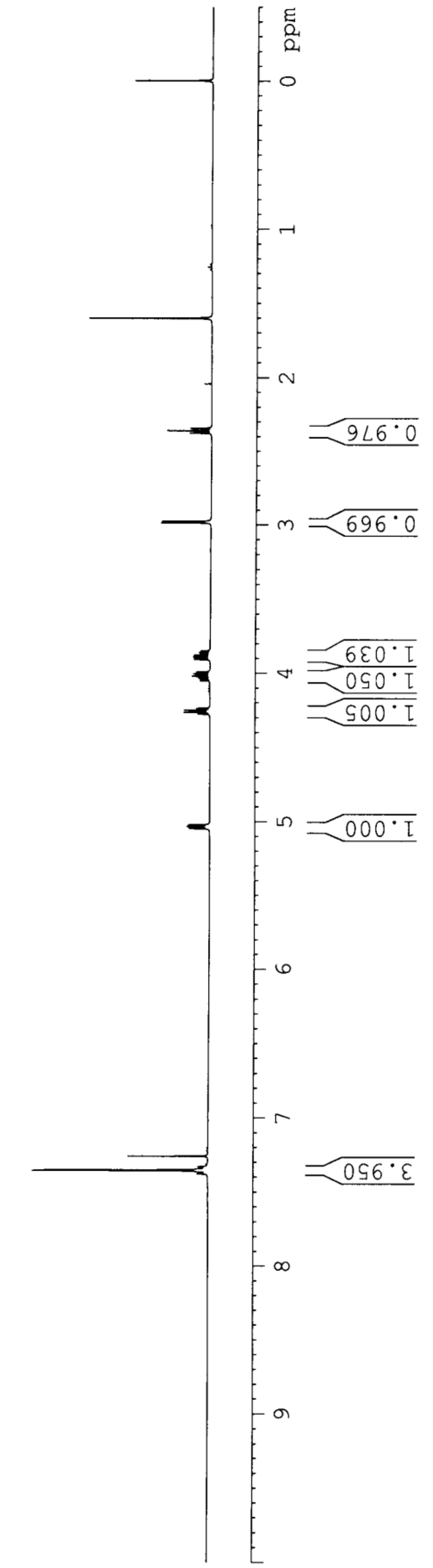

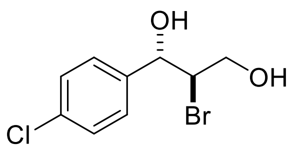

Table 2, Entry 2, **2b**

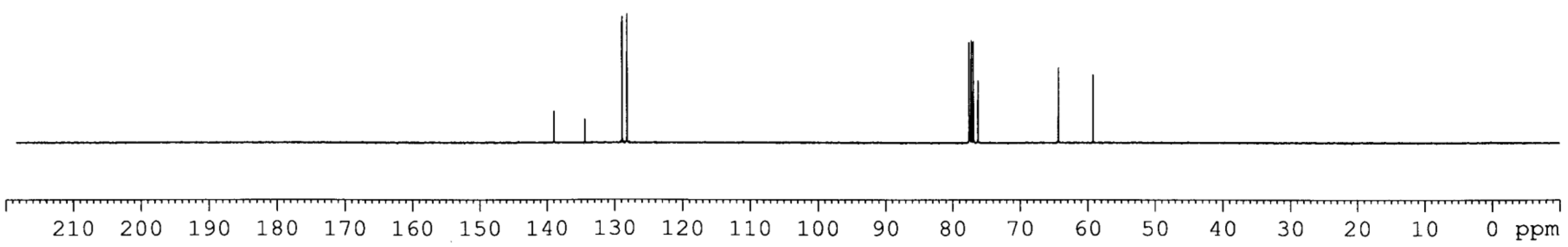

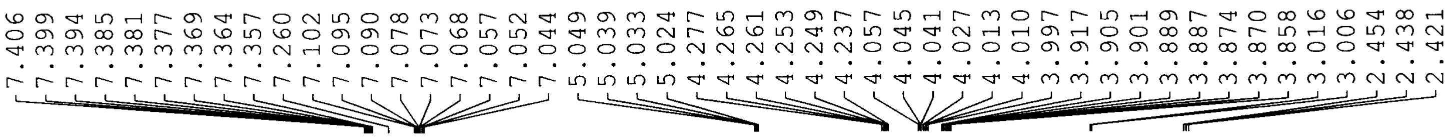

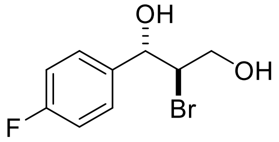

Table 2, Entry 3, **2c**

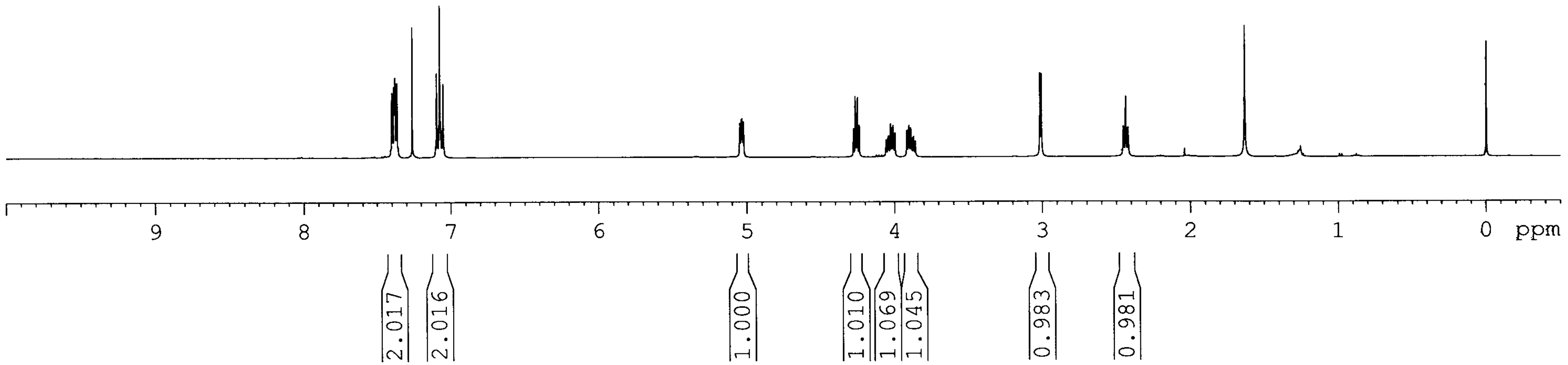

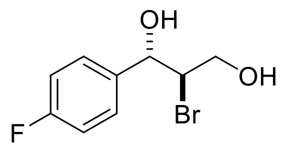

Table 2, Entry 3, **2c**

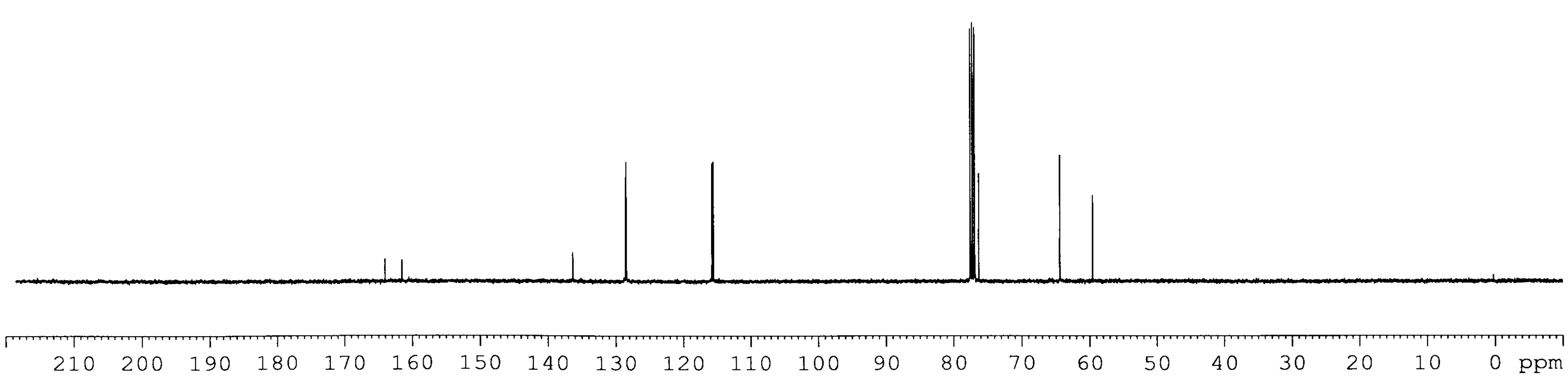

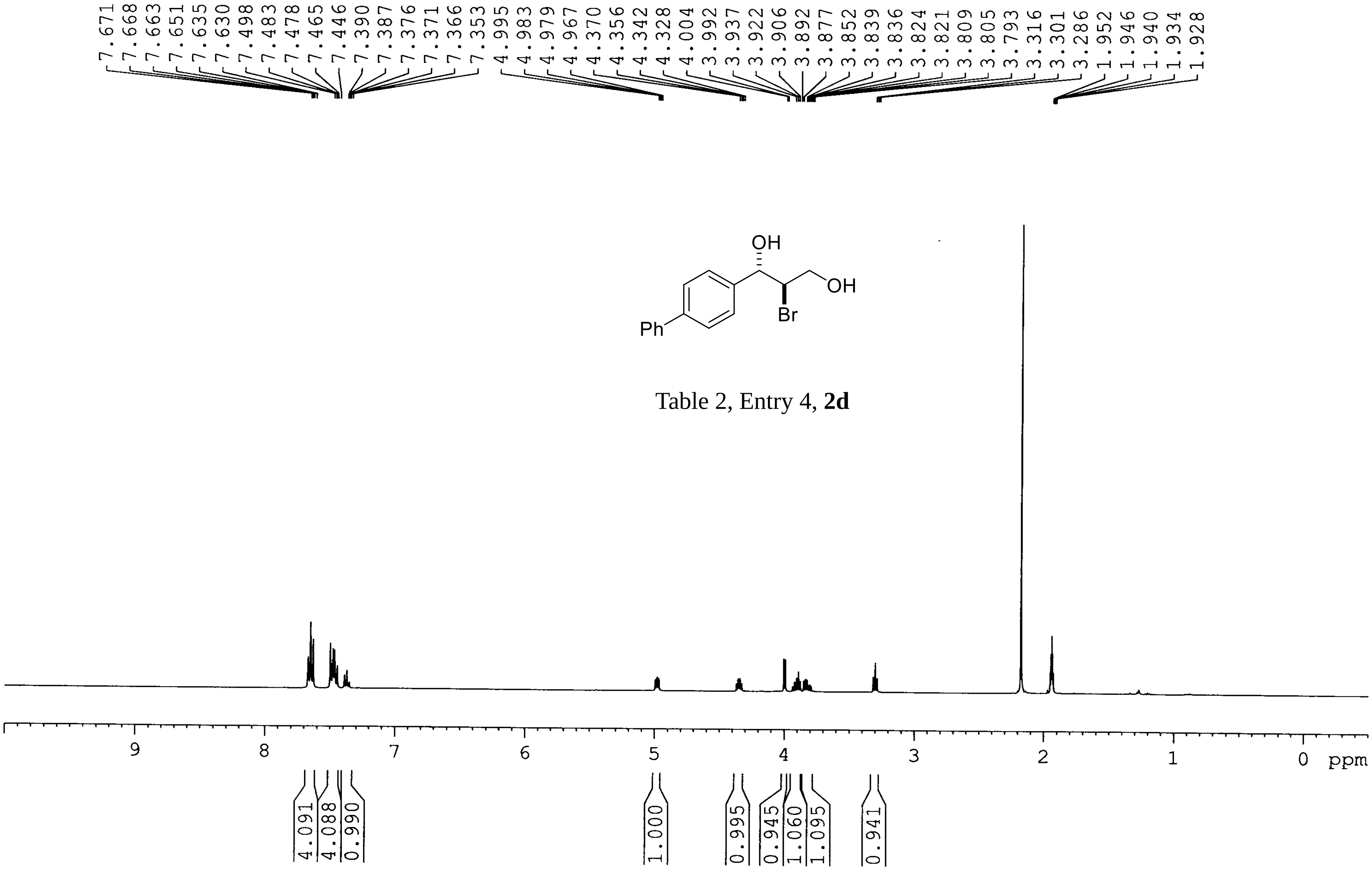

Table 2, Entry 4, **2d**

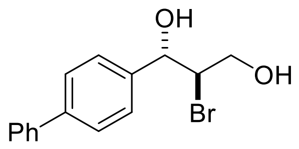

Table 2, Entry 4, **2d**

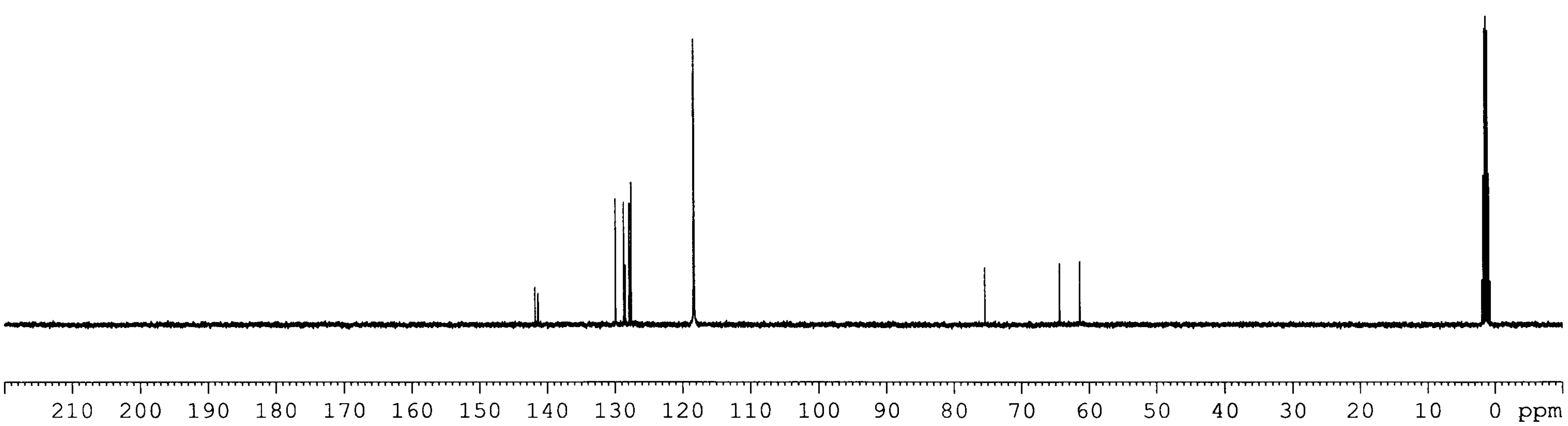

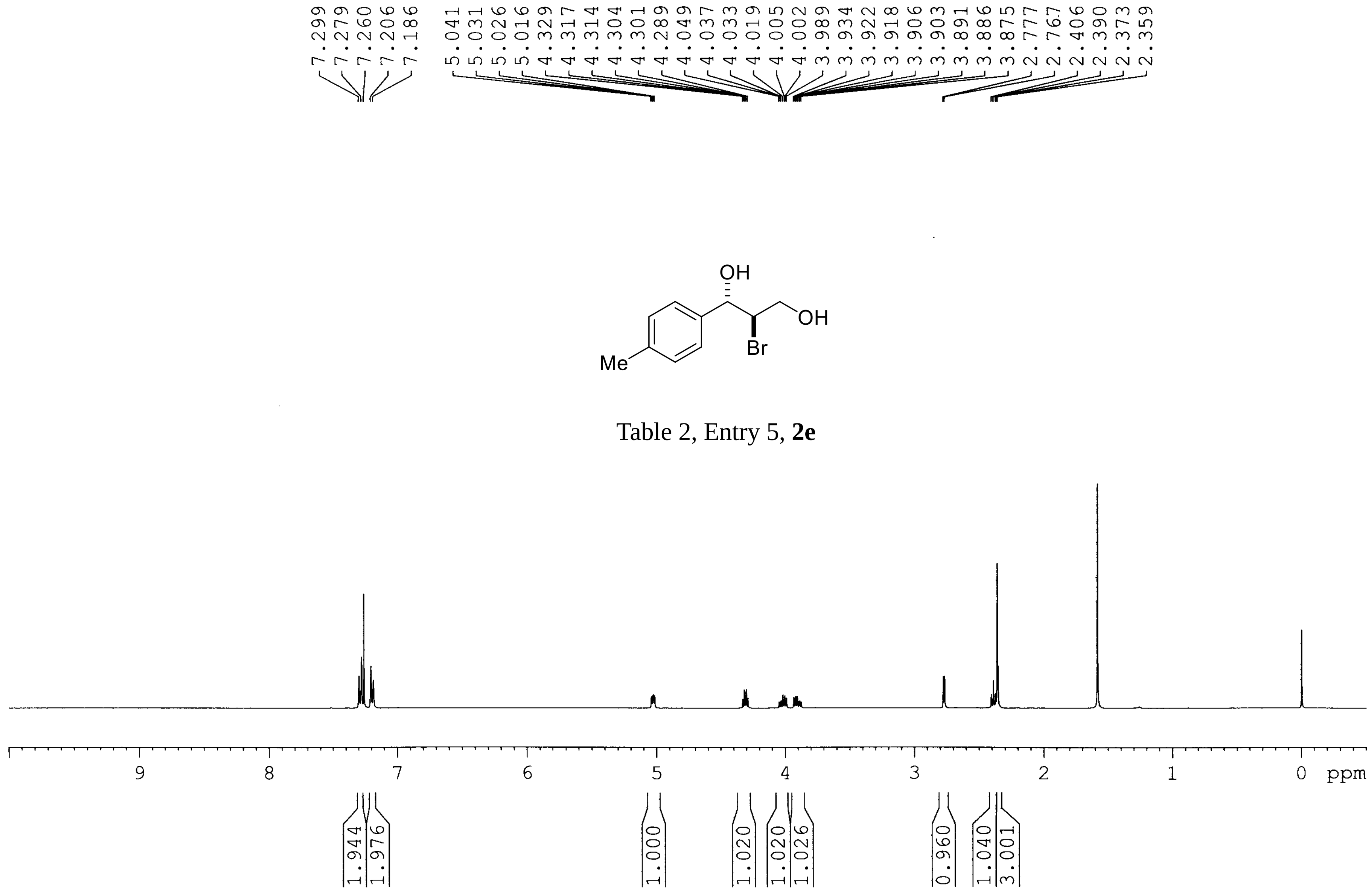

Table 2, Entry 5, **2e**

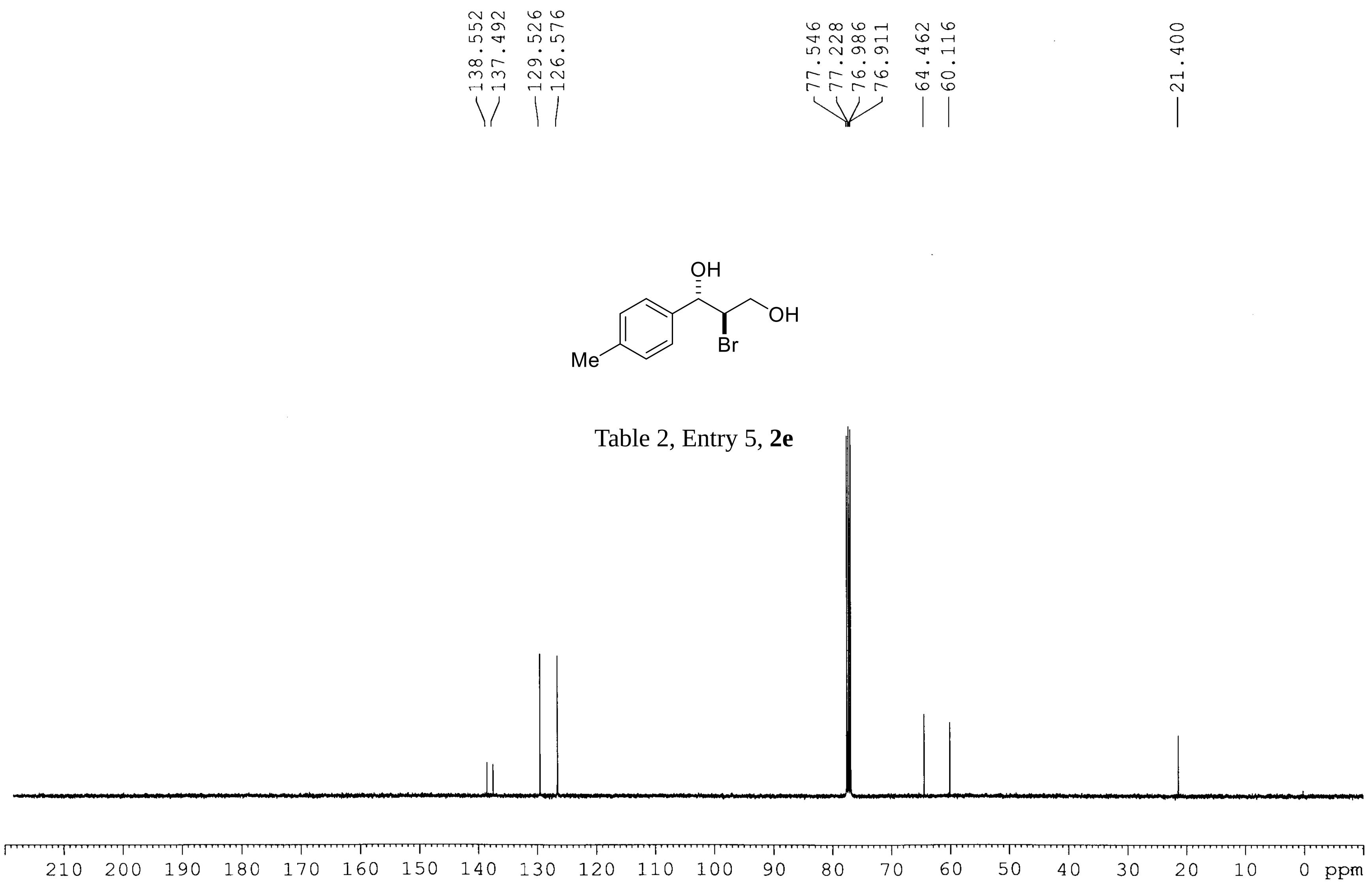

Table 2, Entry 5, **2e**

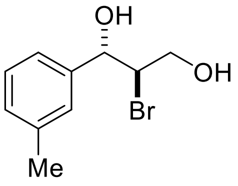

Table 2, Entry 6, **2f**

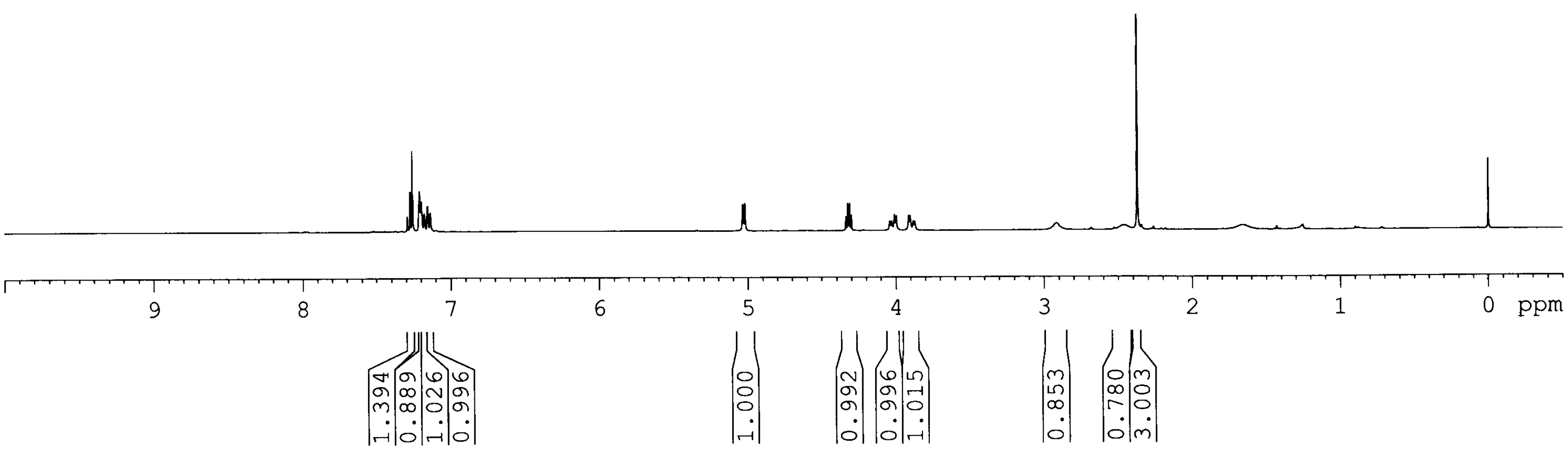

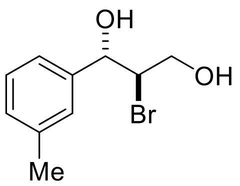

Table 2, Entry 6, **2f**

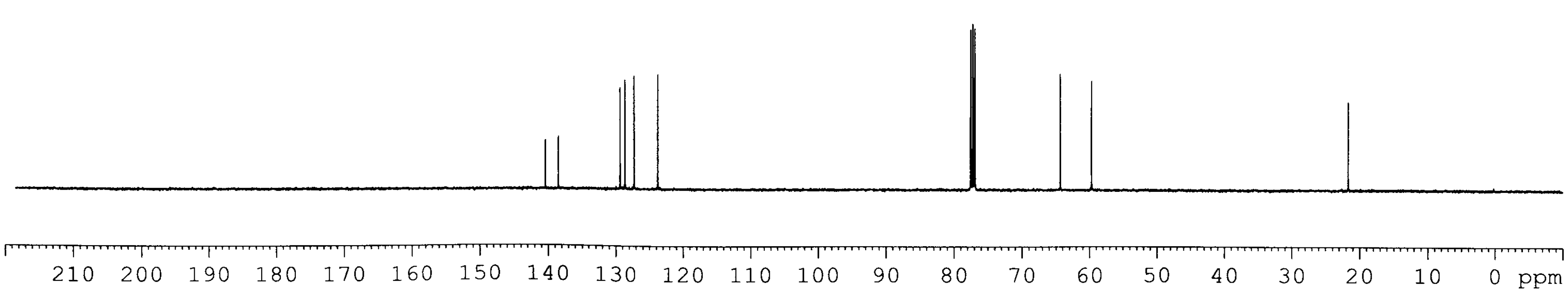

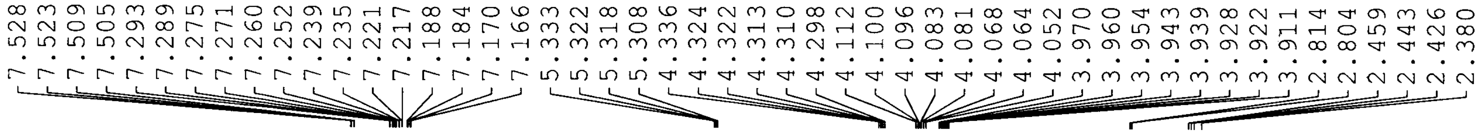

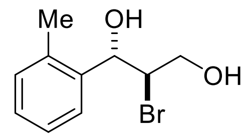

Table 2, Entry 7, **2g**

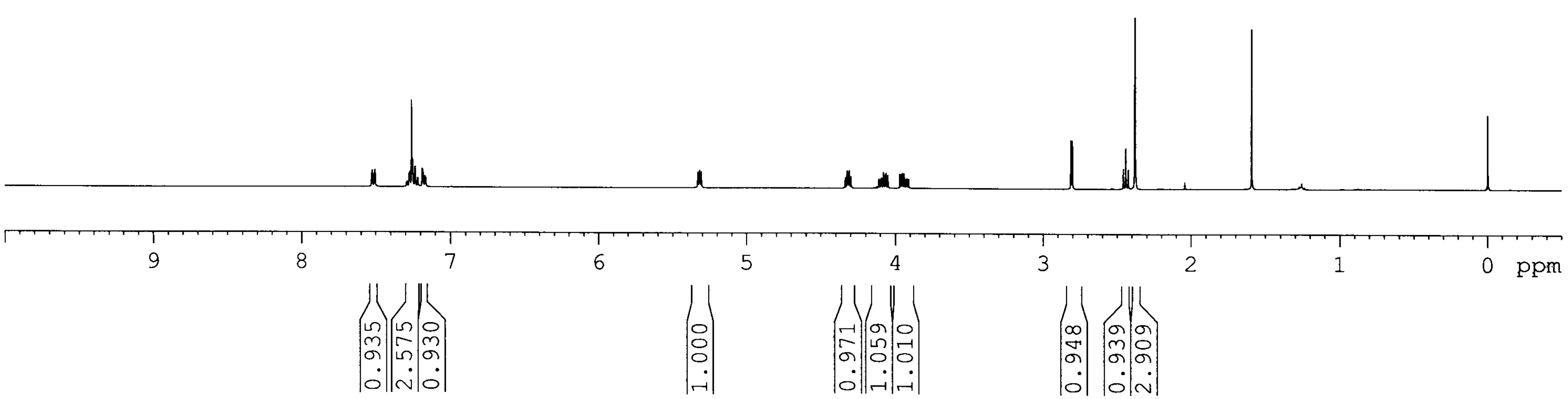

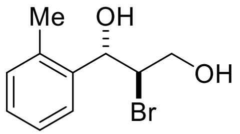

Table 2, Entry 7, **2g**

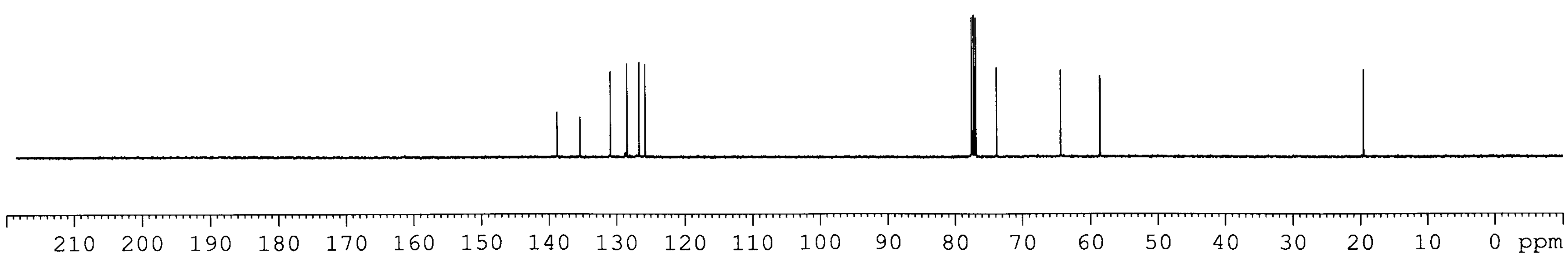

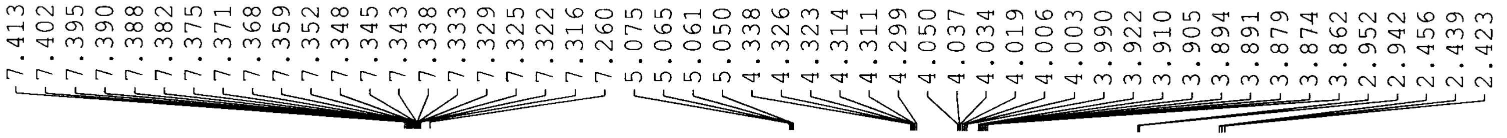

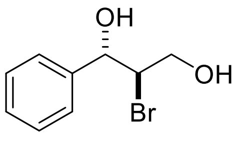

Table 2, Entry 8, **2h**

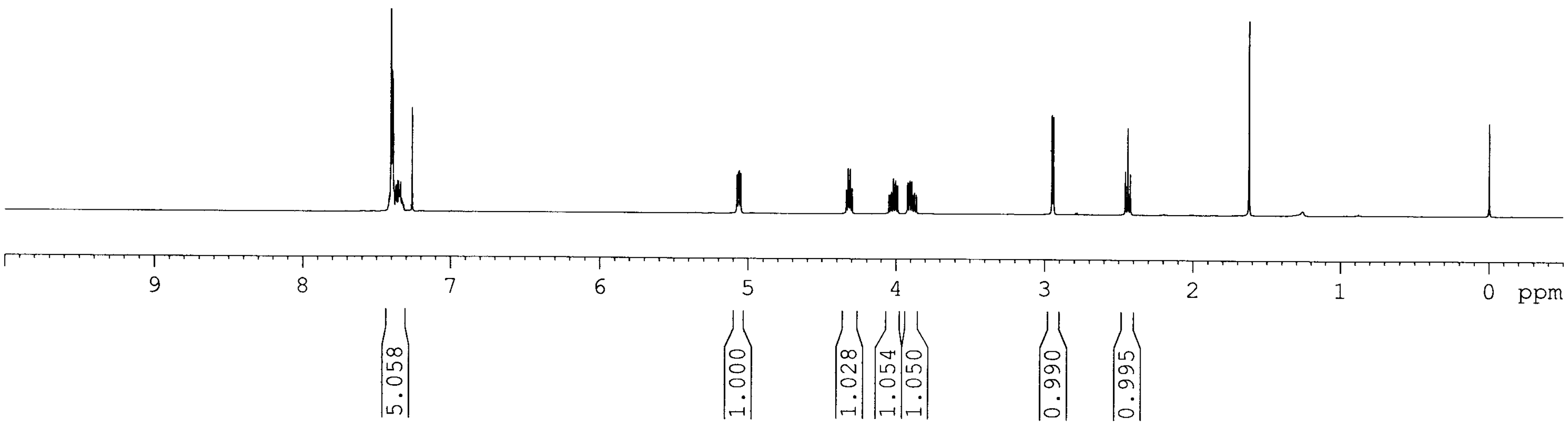

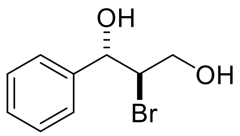

Table 2, Entry 8, **2h**

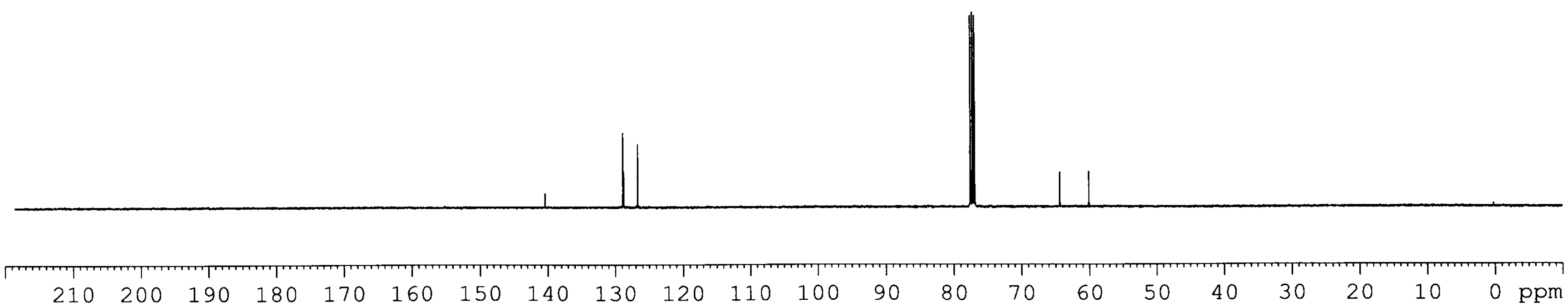

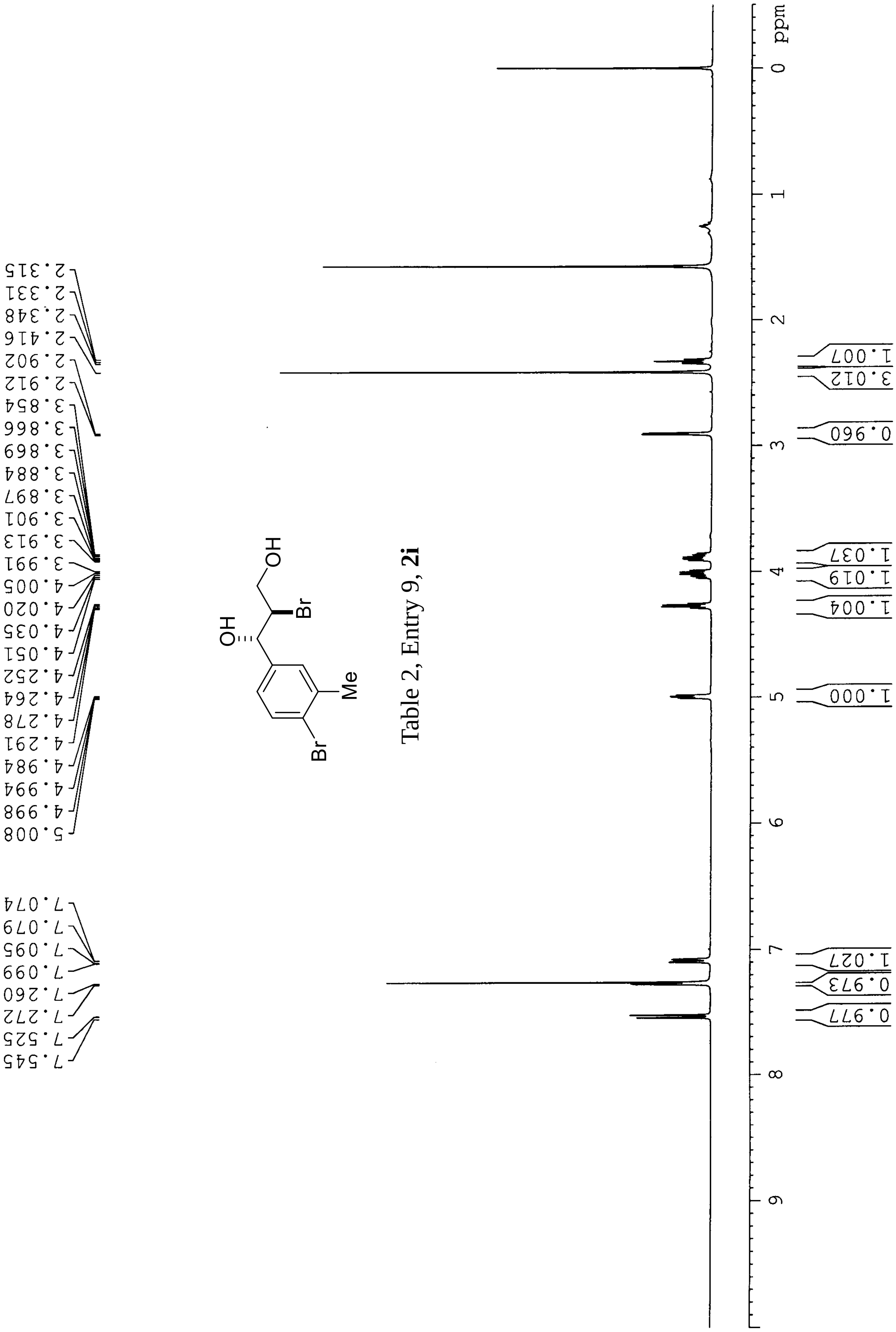

Table 2, Entry 9, **2i**

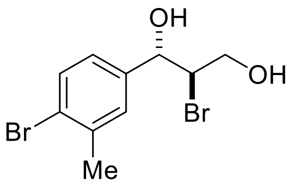

Table 2, Entry 9, **2i**

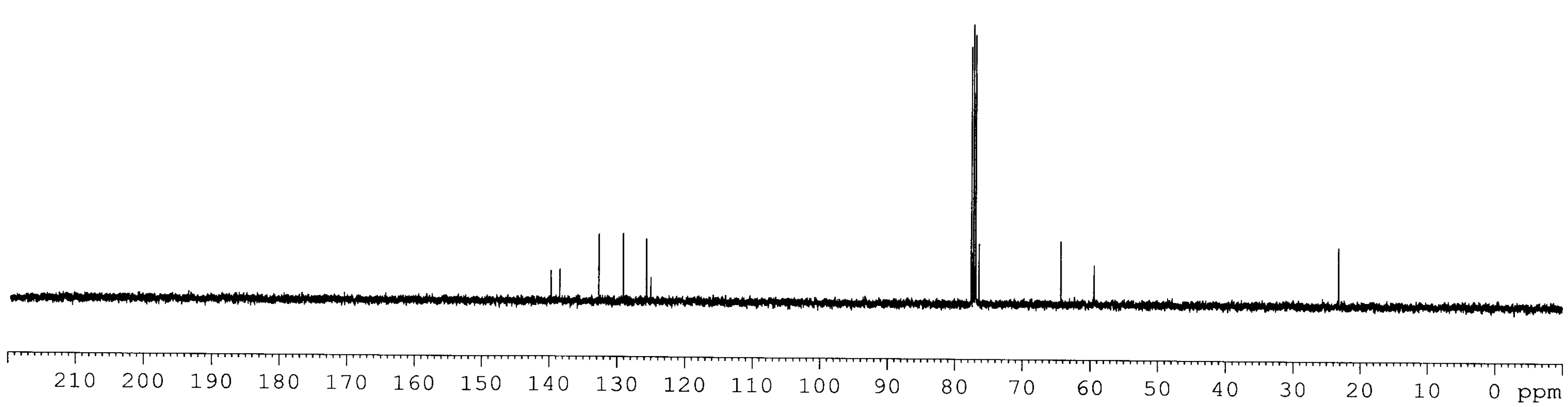

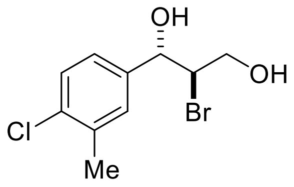

Table 2, Entry 10, **2j**

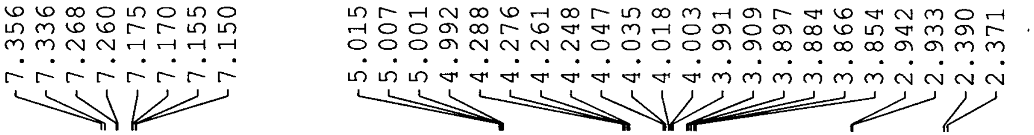

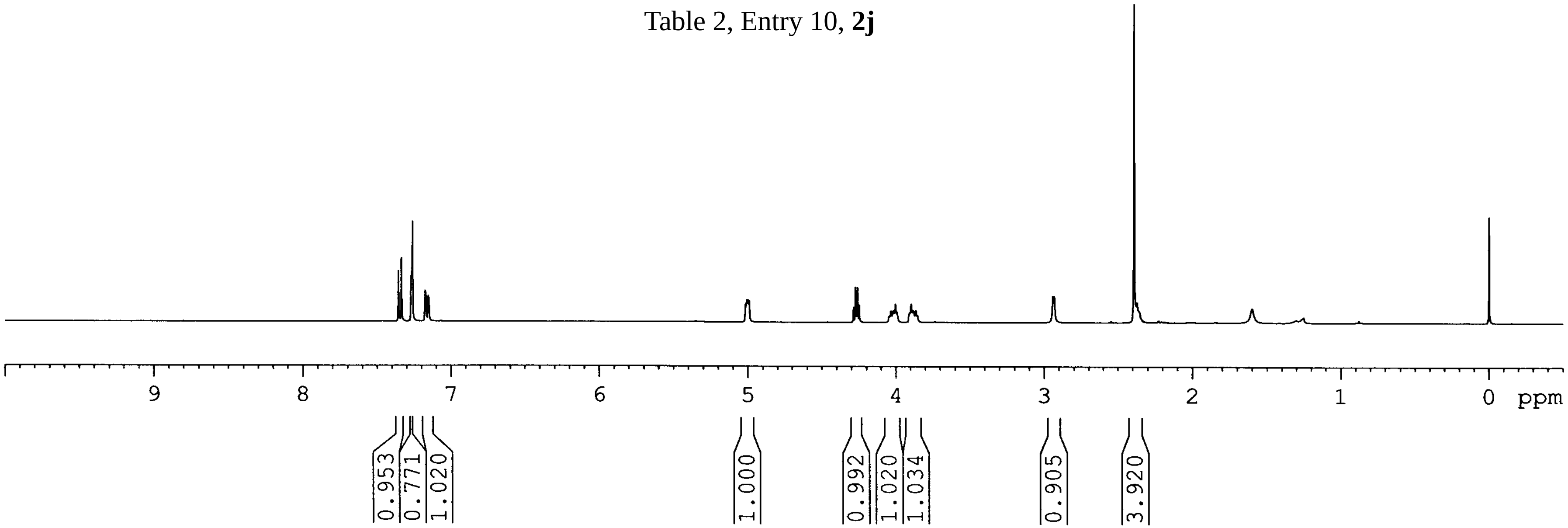

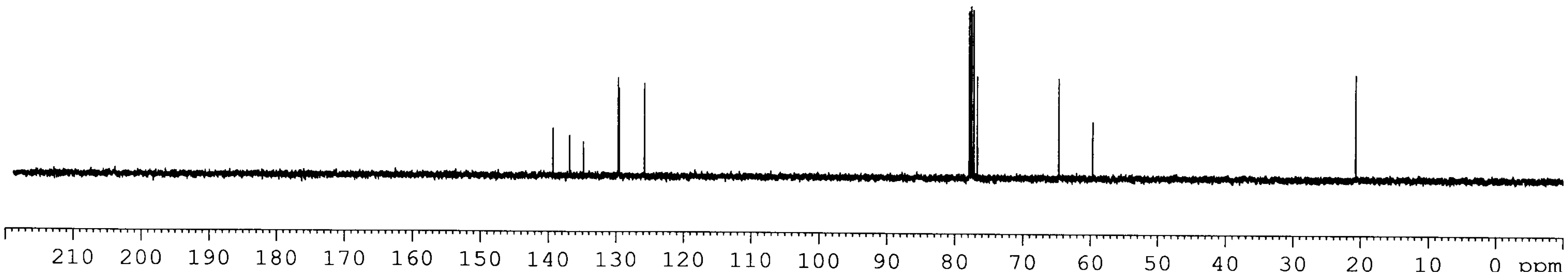

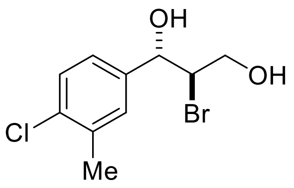

Table 2, Entry 10, **2j**

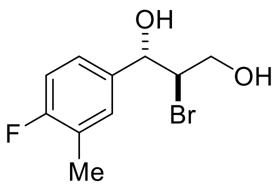

Table 2, Entry 11, **2k**

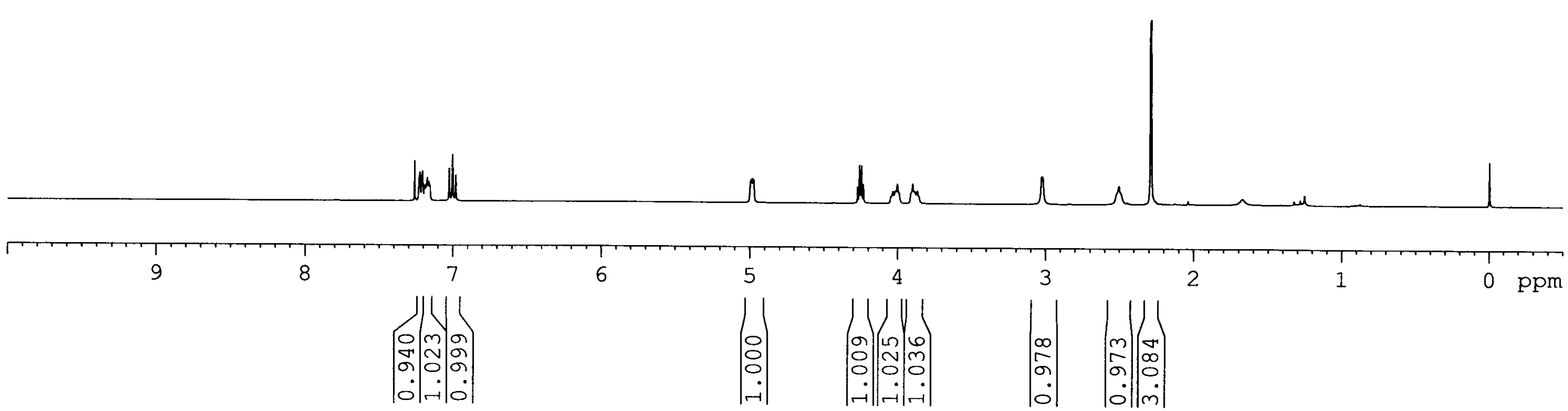

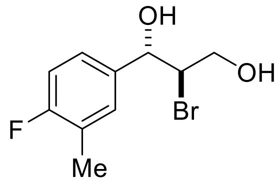

Table 2, Entry 11, **2k**

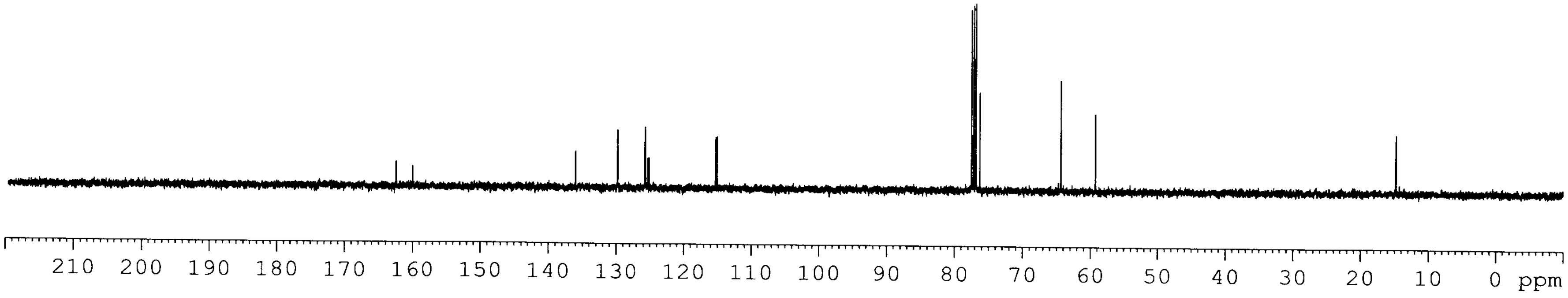

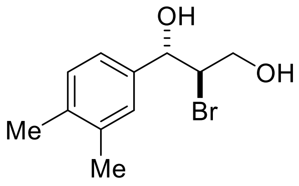

Table 2, Entry 12, **2l**

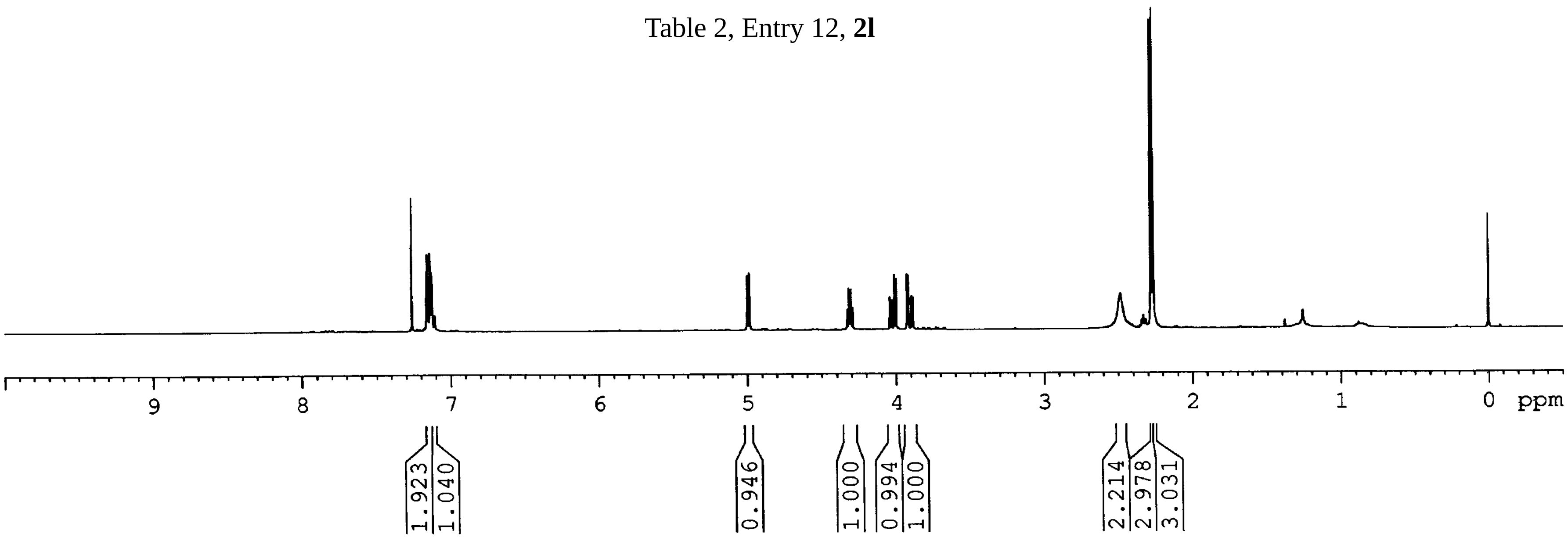

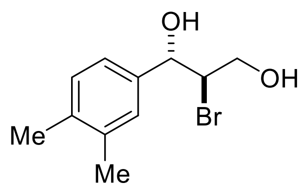

Table 2, Entry 12, **2l**

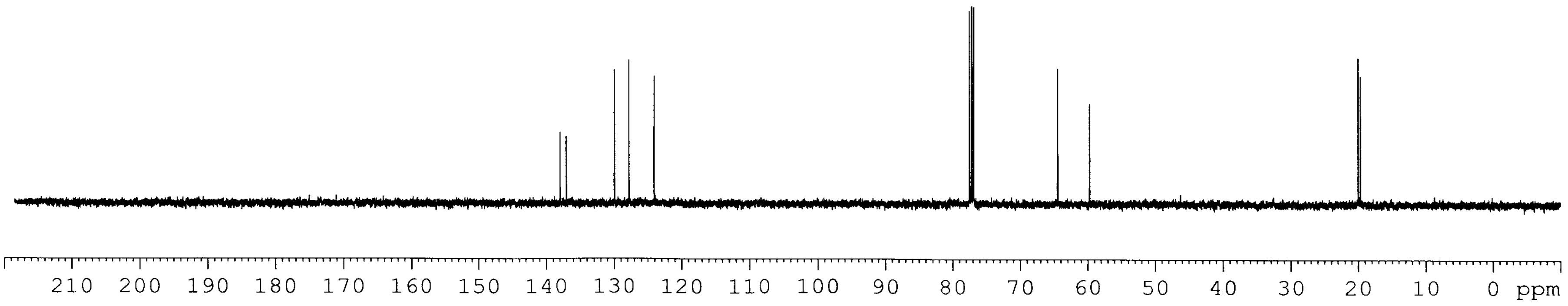

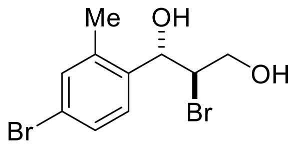

Table 2, Entry 13, **2m**

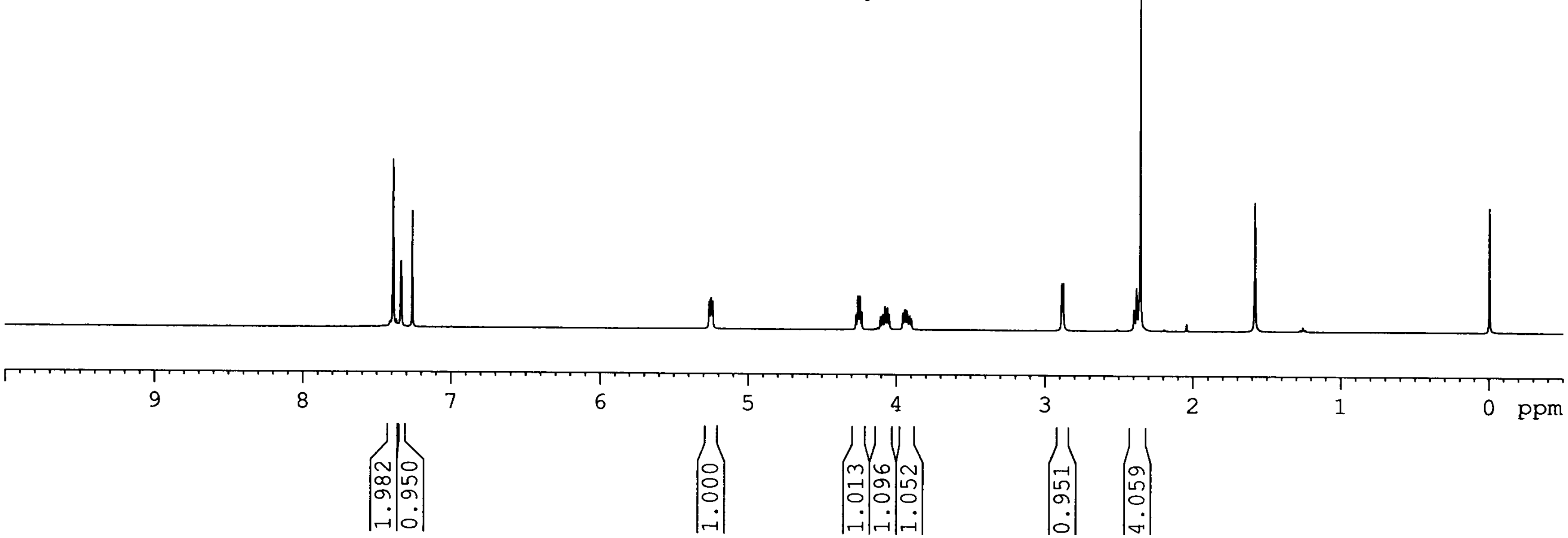

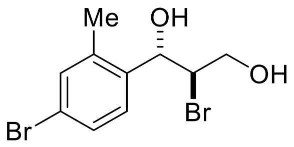

Table 2, Entry 13, **2m**

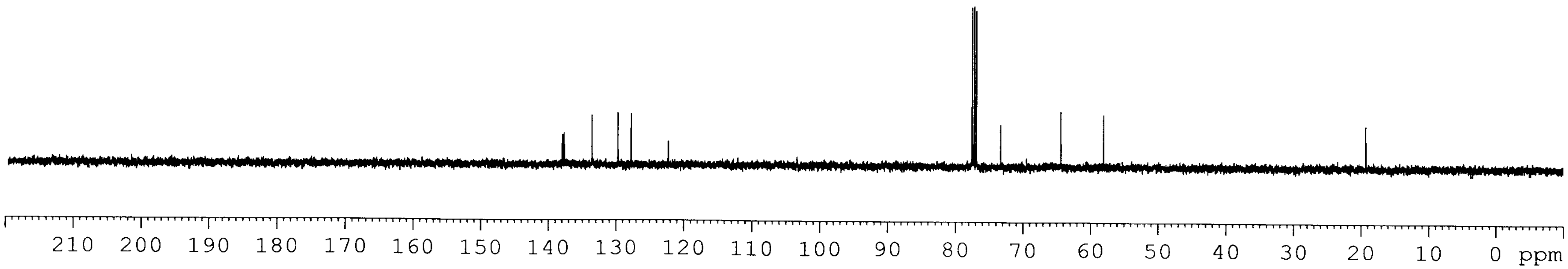

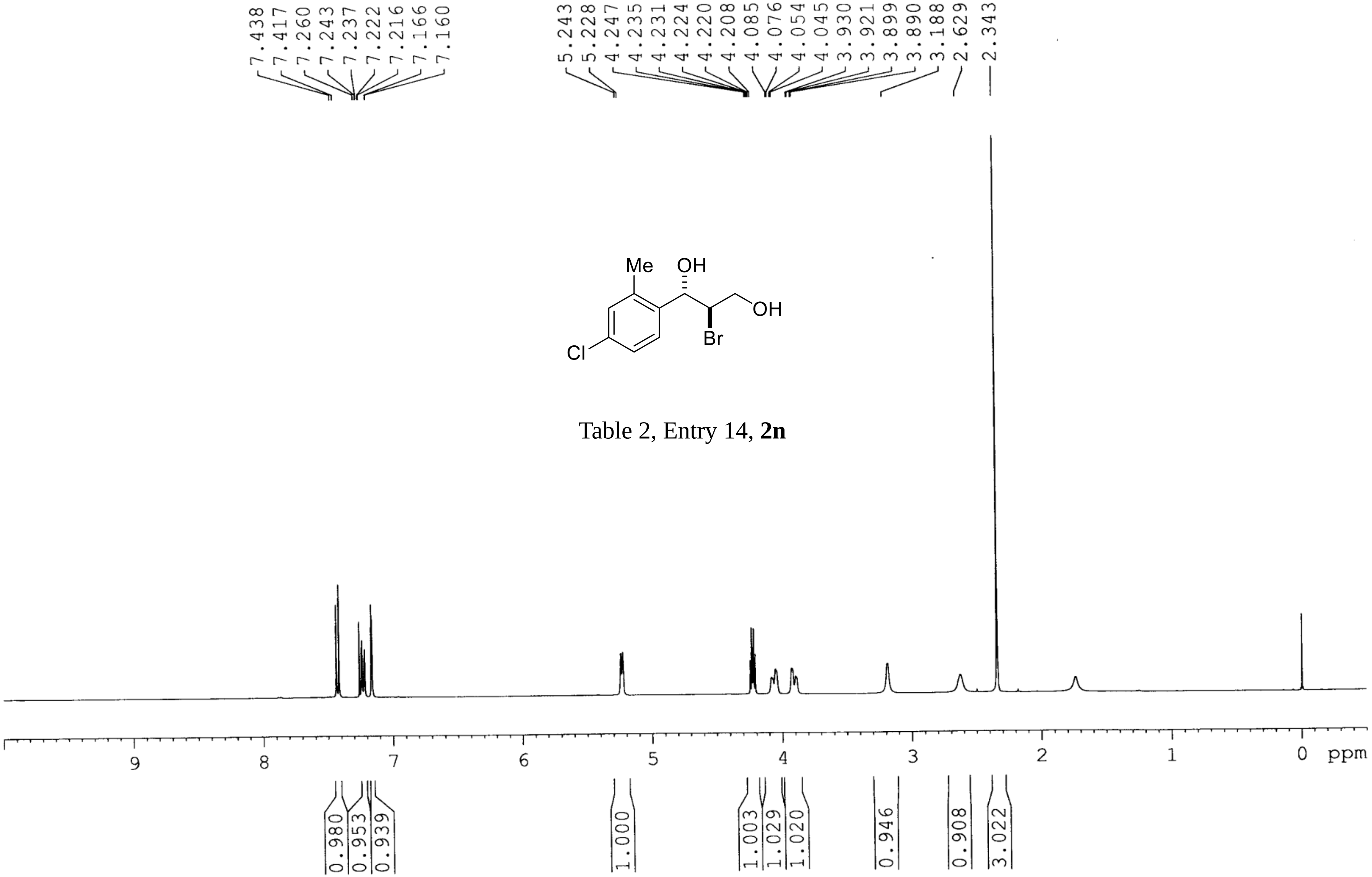

Table 2, Entry 14, **2n**

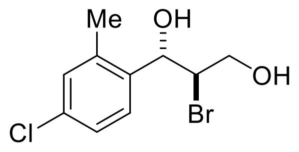

Table 2, Entry 14, **2n**

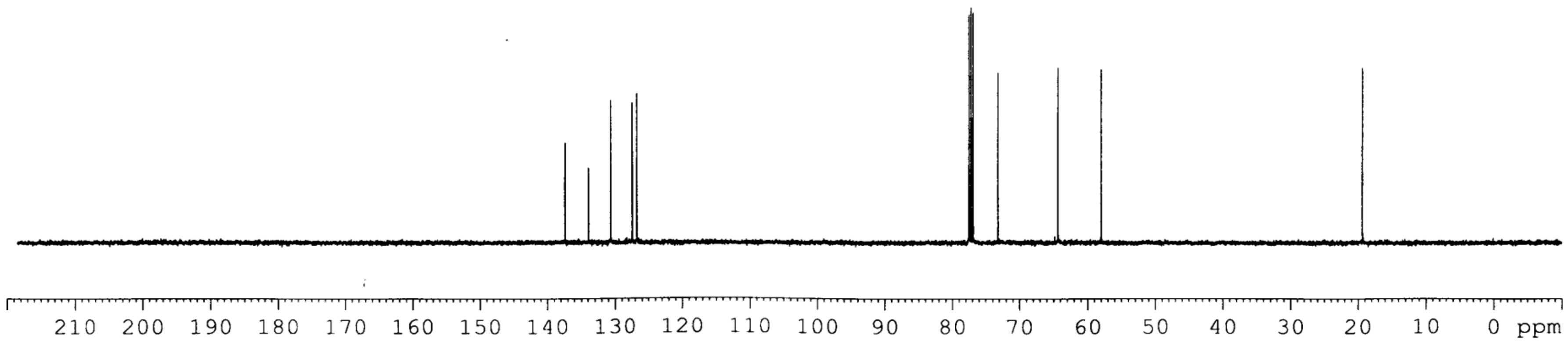

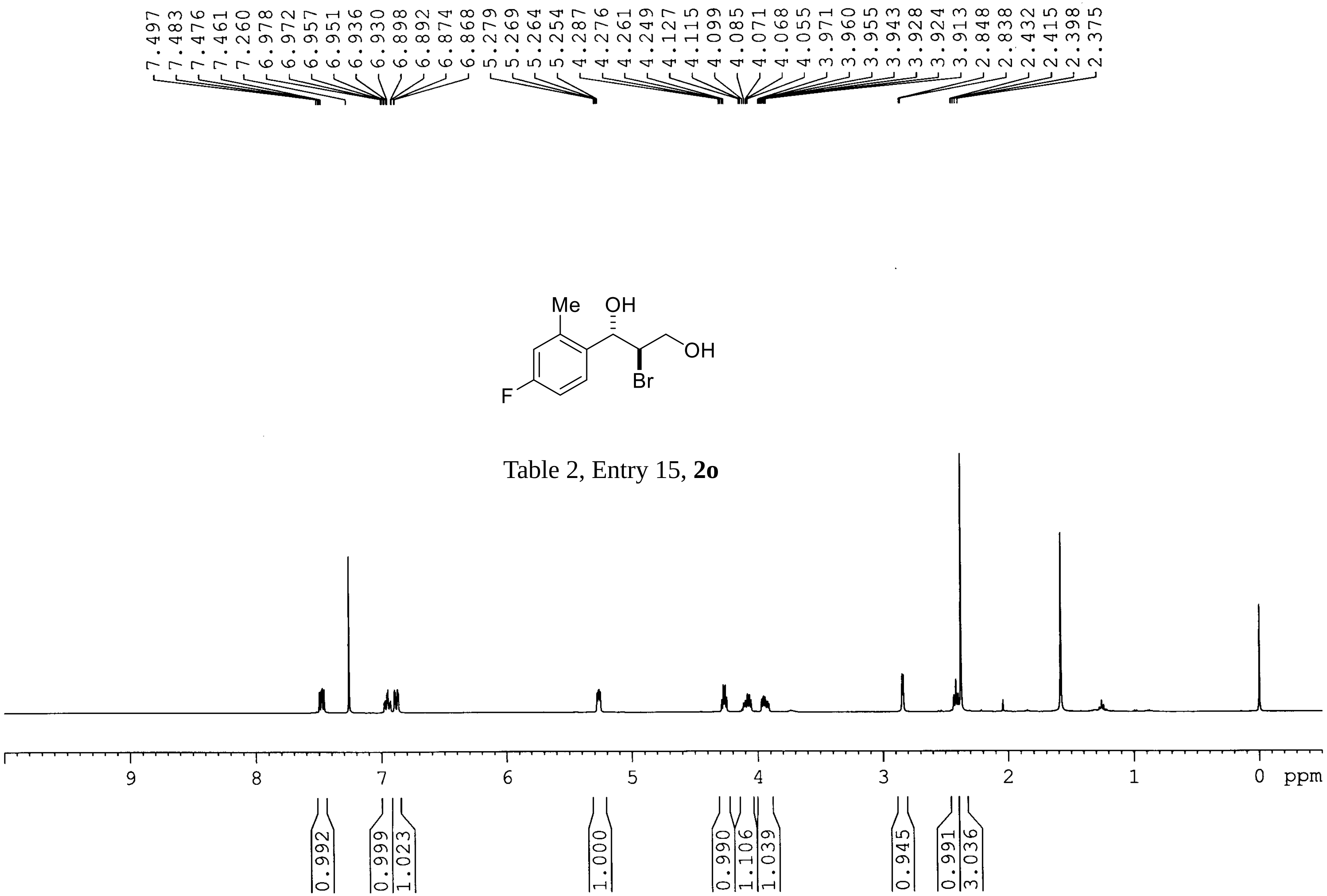

Table 2, Entry 15, **2o**

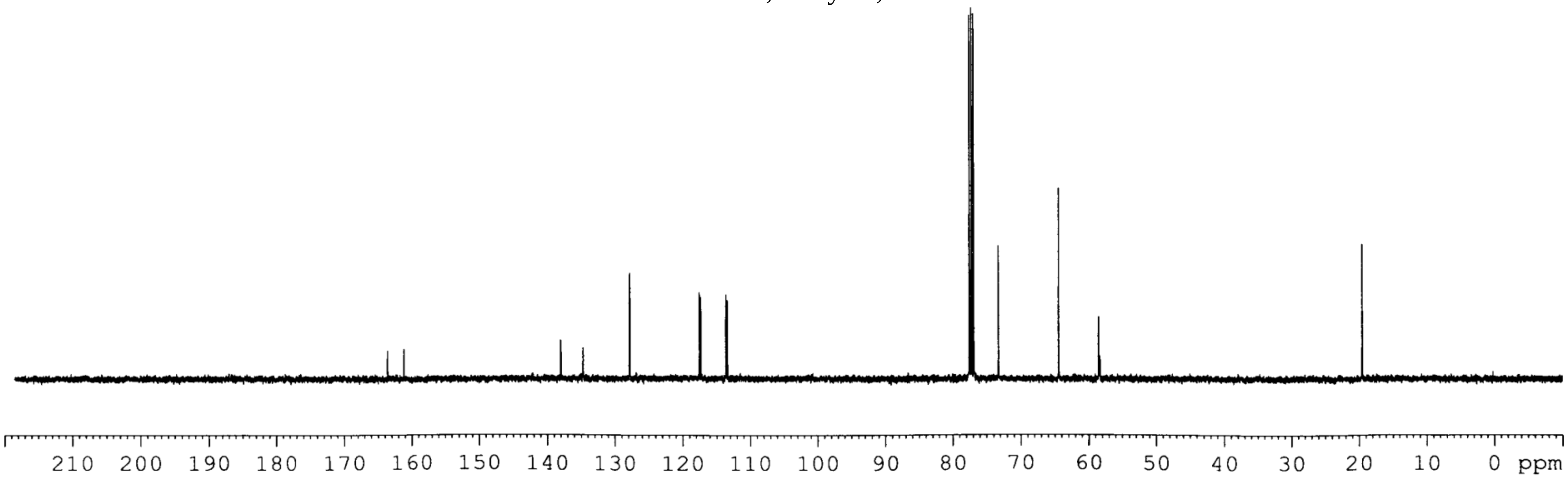

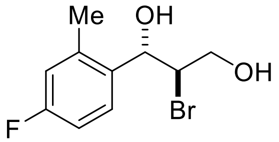

Table 2, Entry 15, **2o**

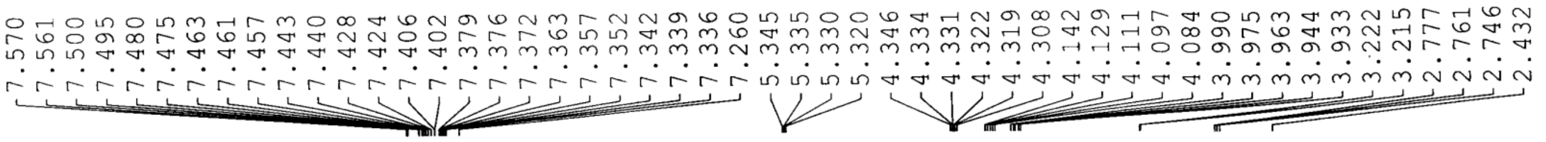

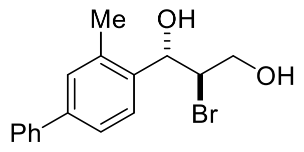

Table 2, Entry 16, **2p**

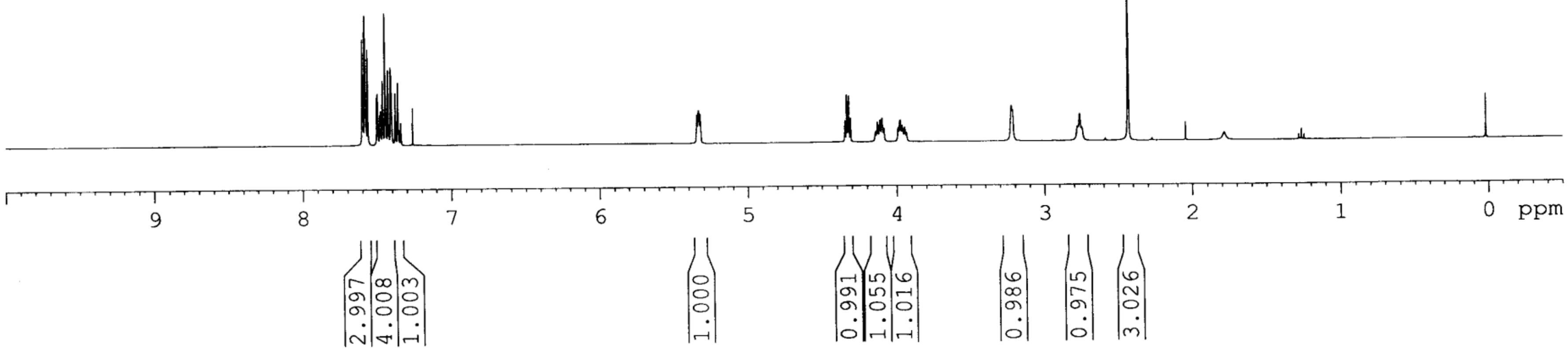

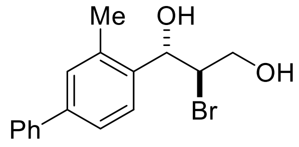

Table 2, Entry 16, **2p**

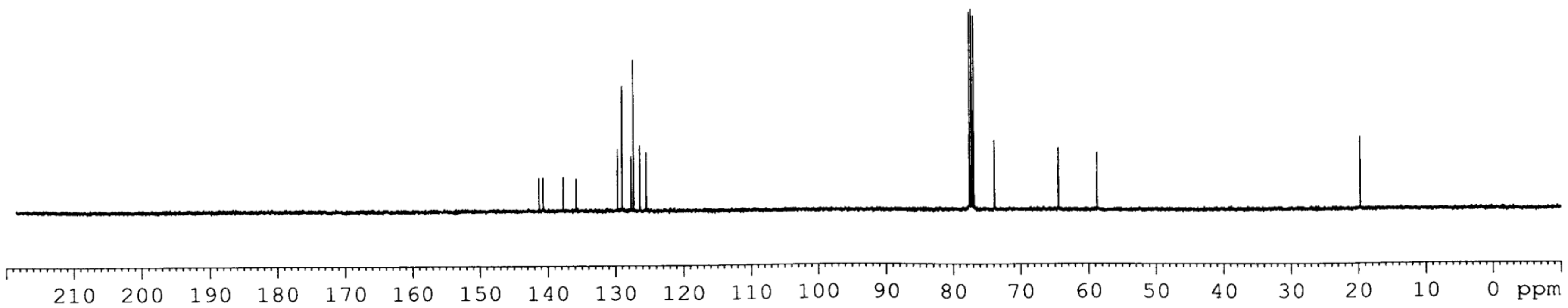

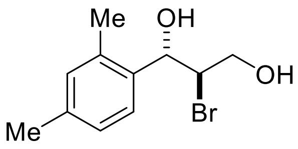

Table 2, Entry 17, **2q**

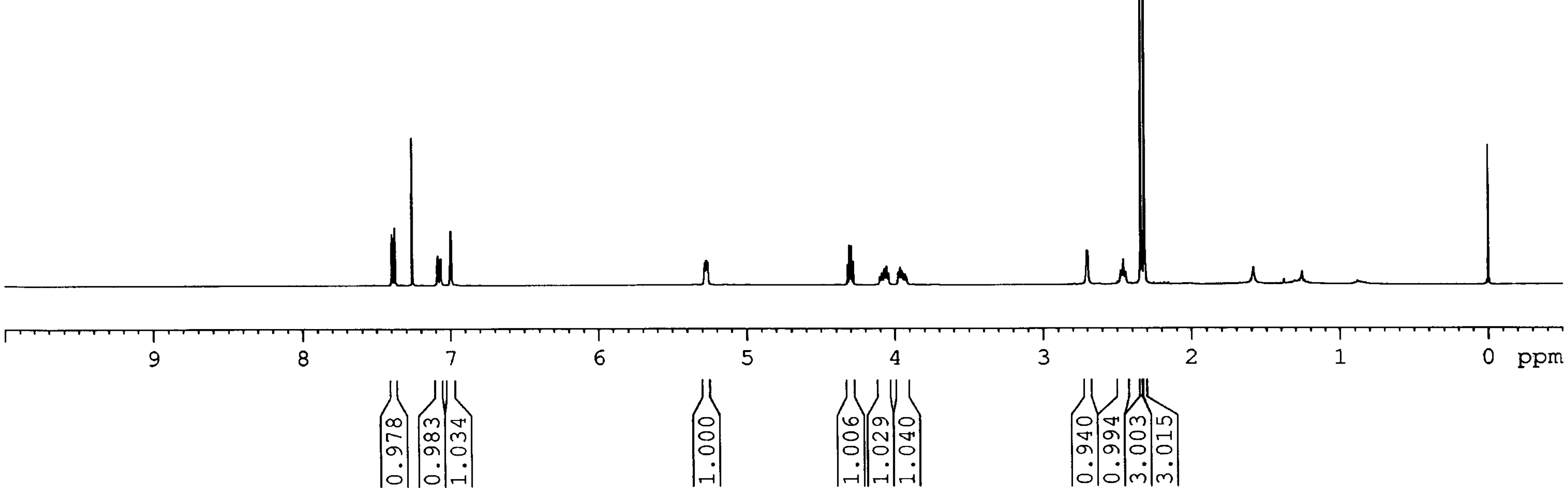

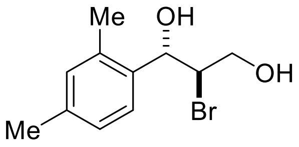

Table 2, Entry 17, **2q**

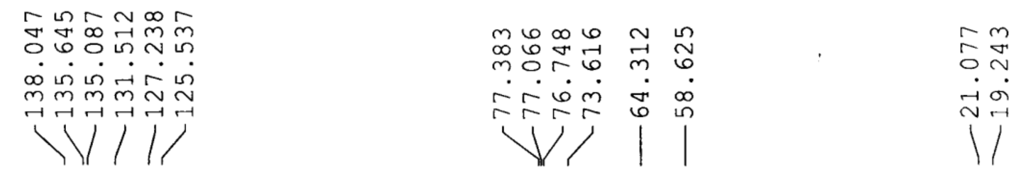

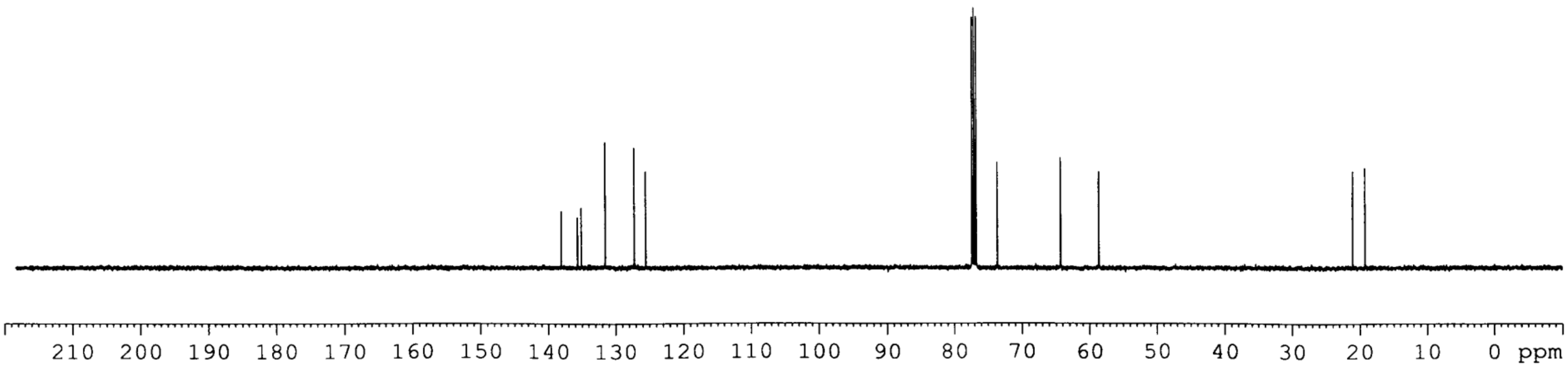

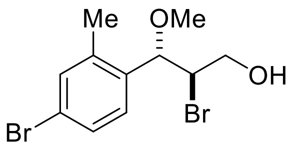

Table 2, Entry 18, **2r**

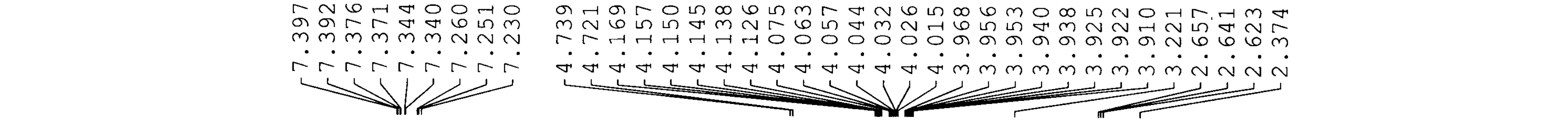

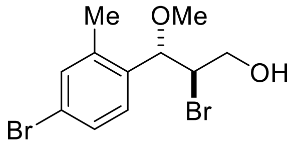

Table 2, Entry 18, **2r**

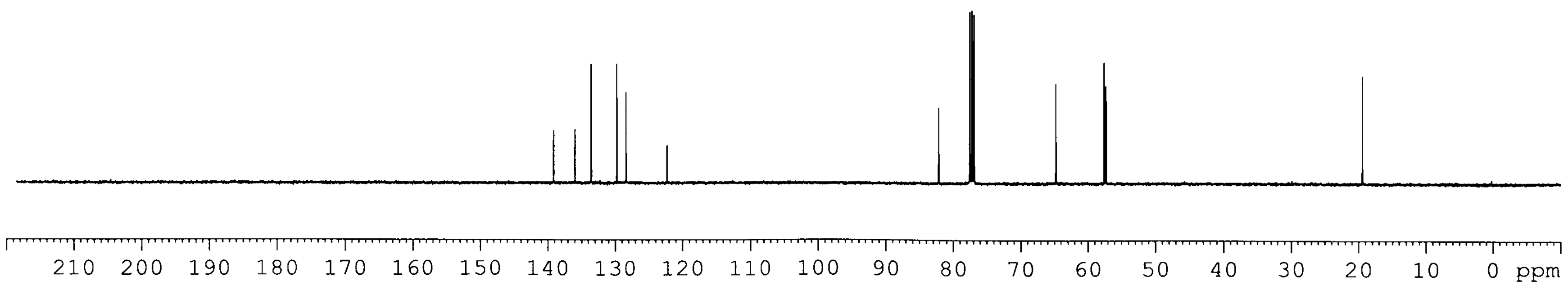

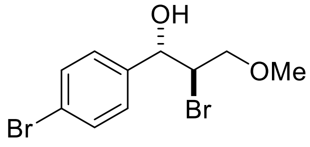

Table 2, Entry 19, **2s**

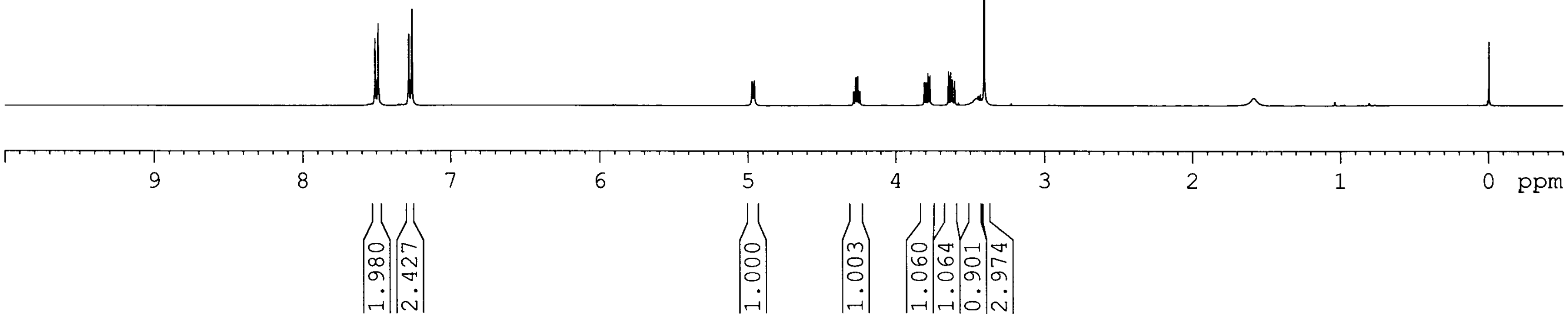

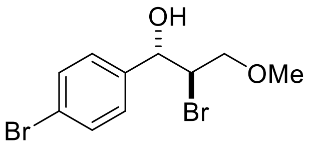

Table 2, Entry 19, **2s**

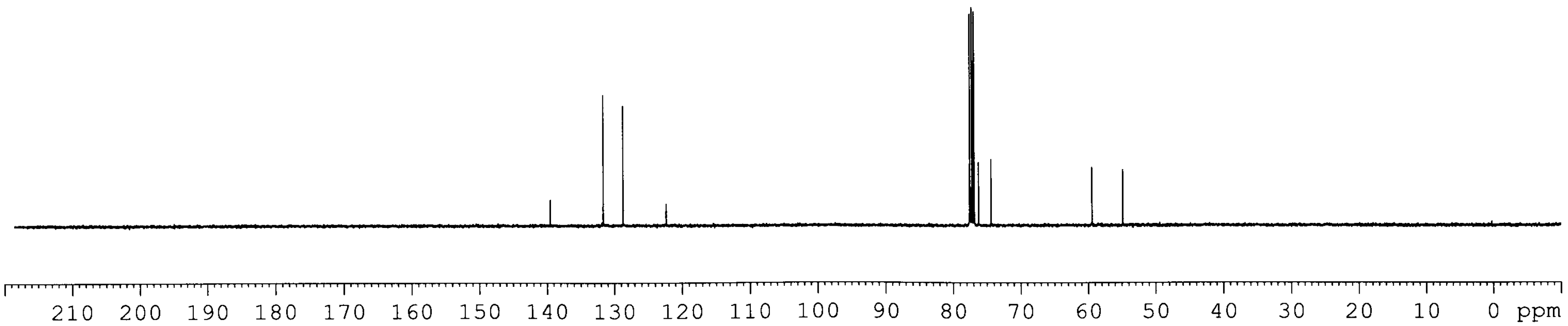

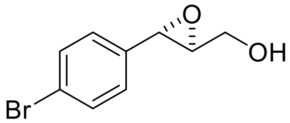

Scheme 3, epoxide **4**

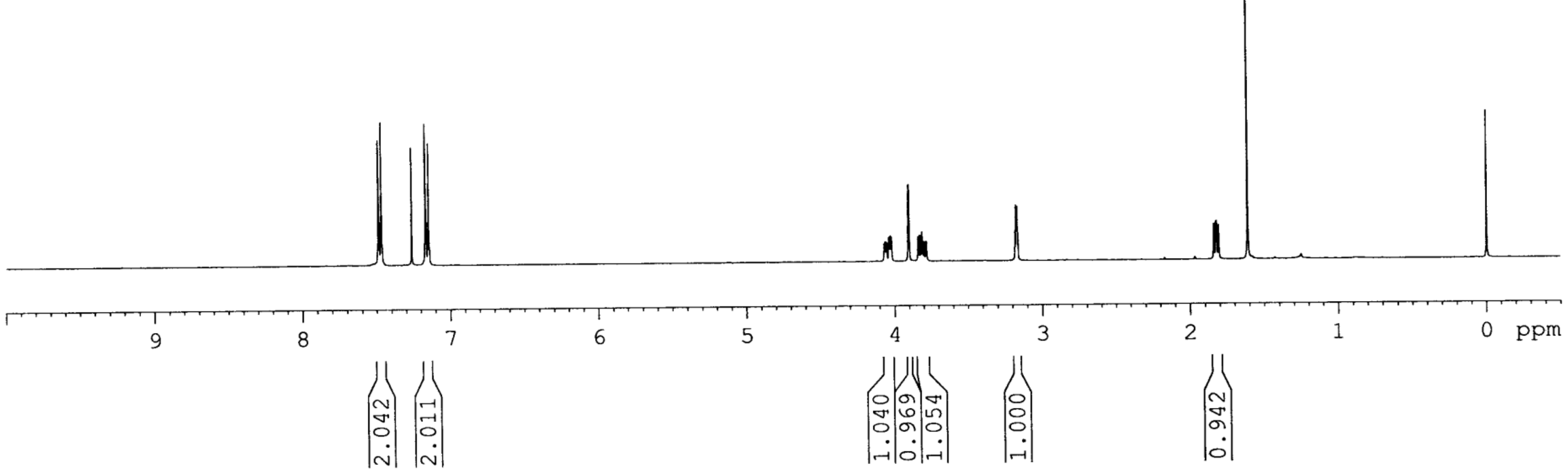

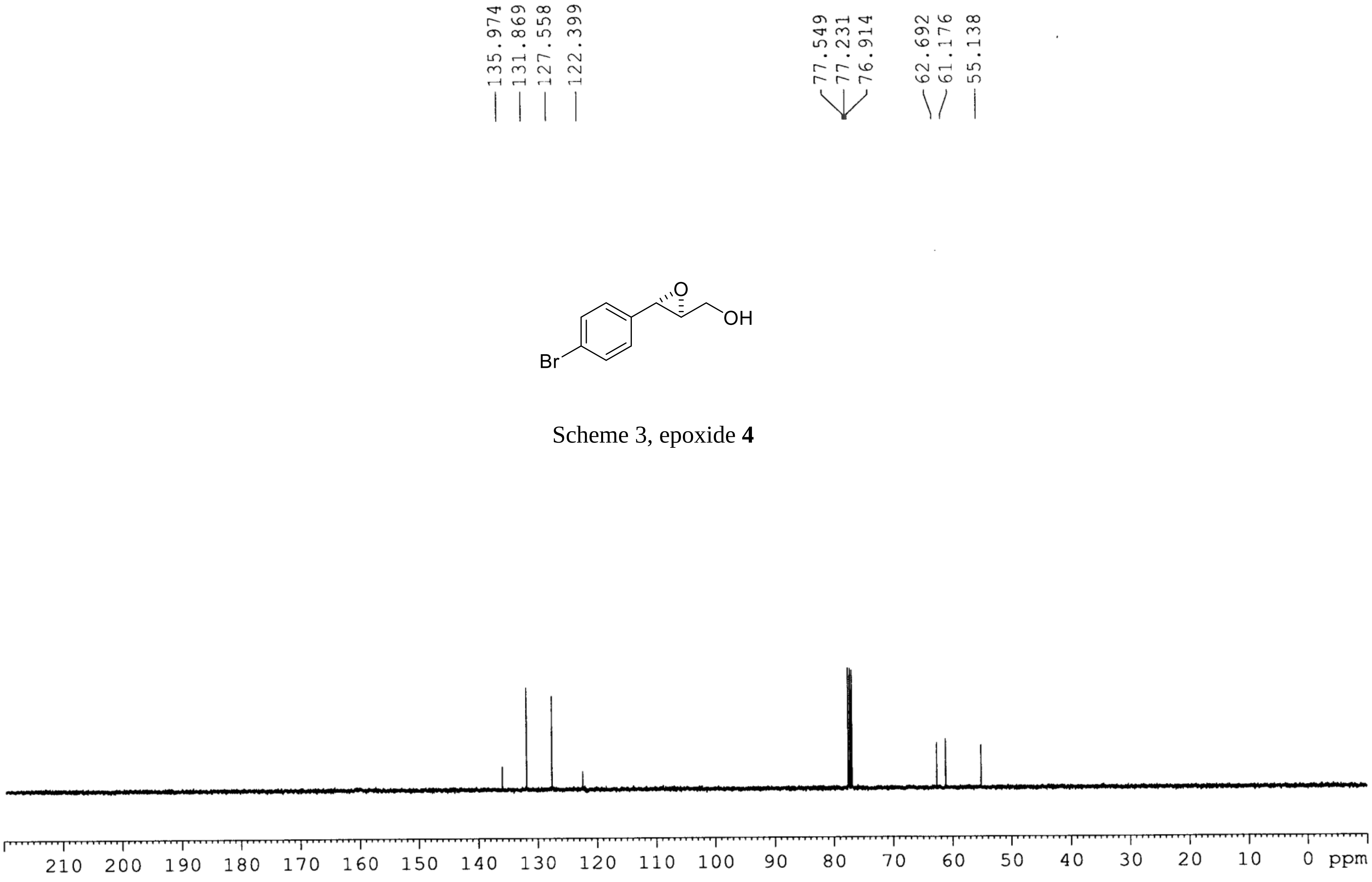

Scheme 3, epoxide **4**

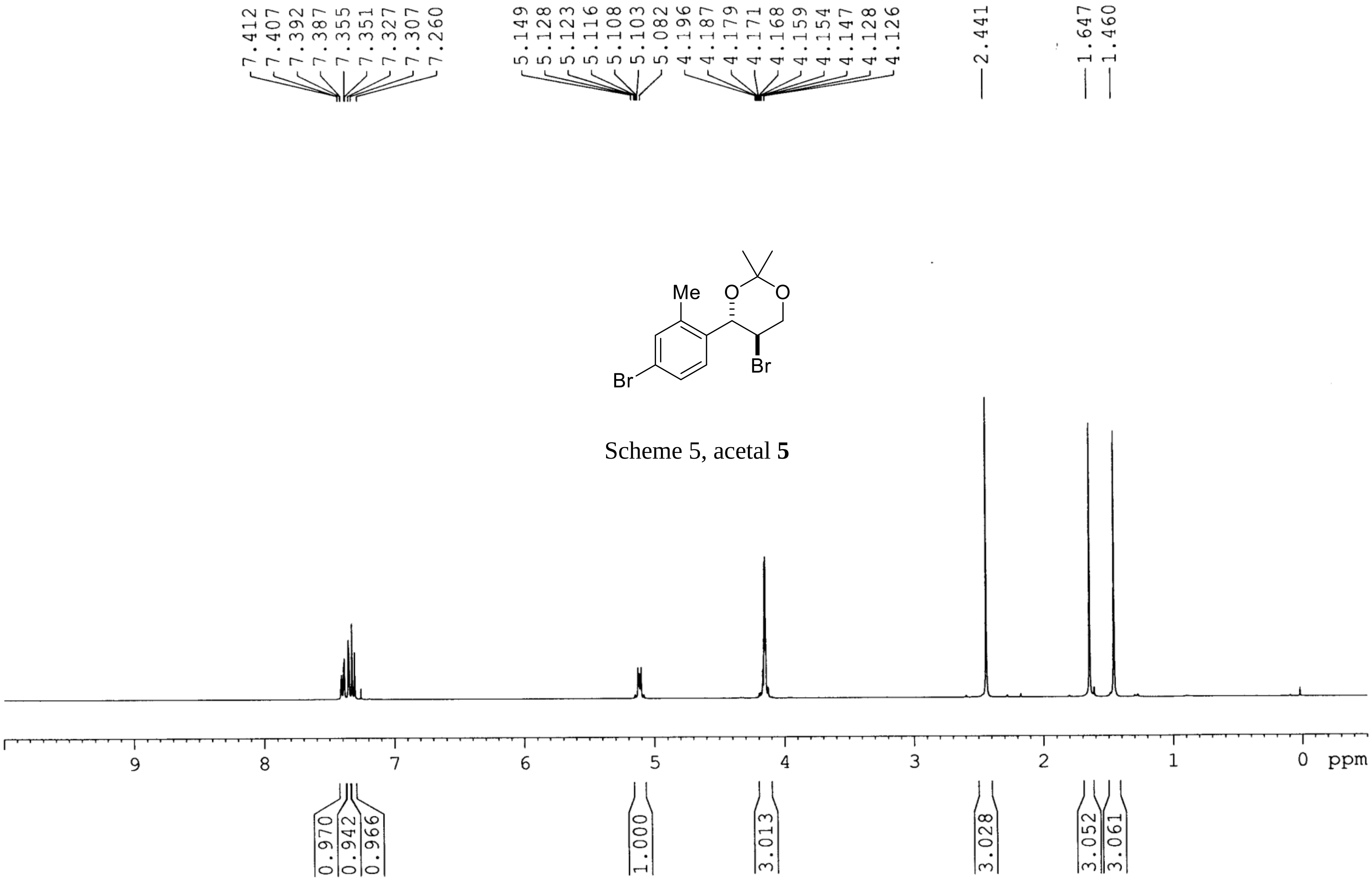

Scheme 5, acetal **5**

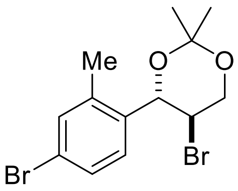

Scheme 5, acetal **5**

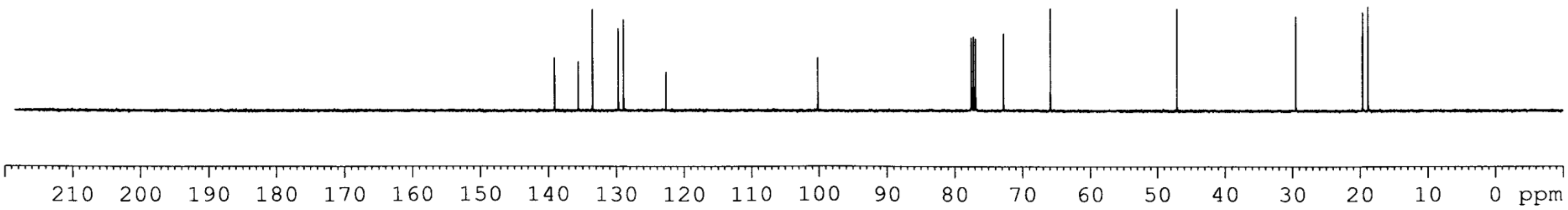

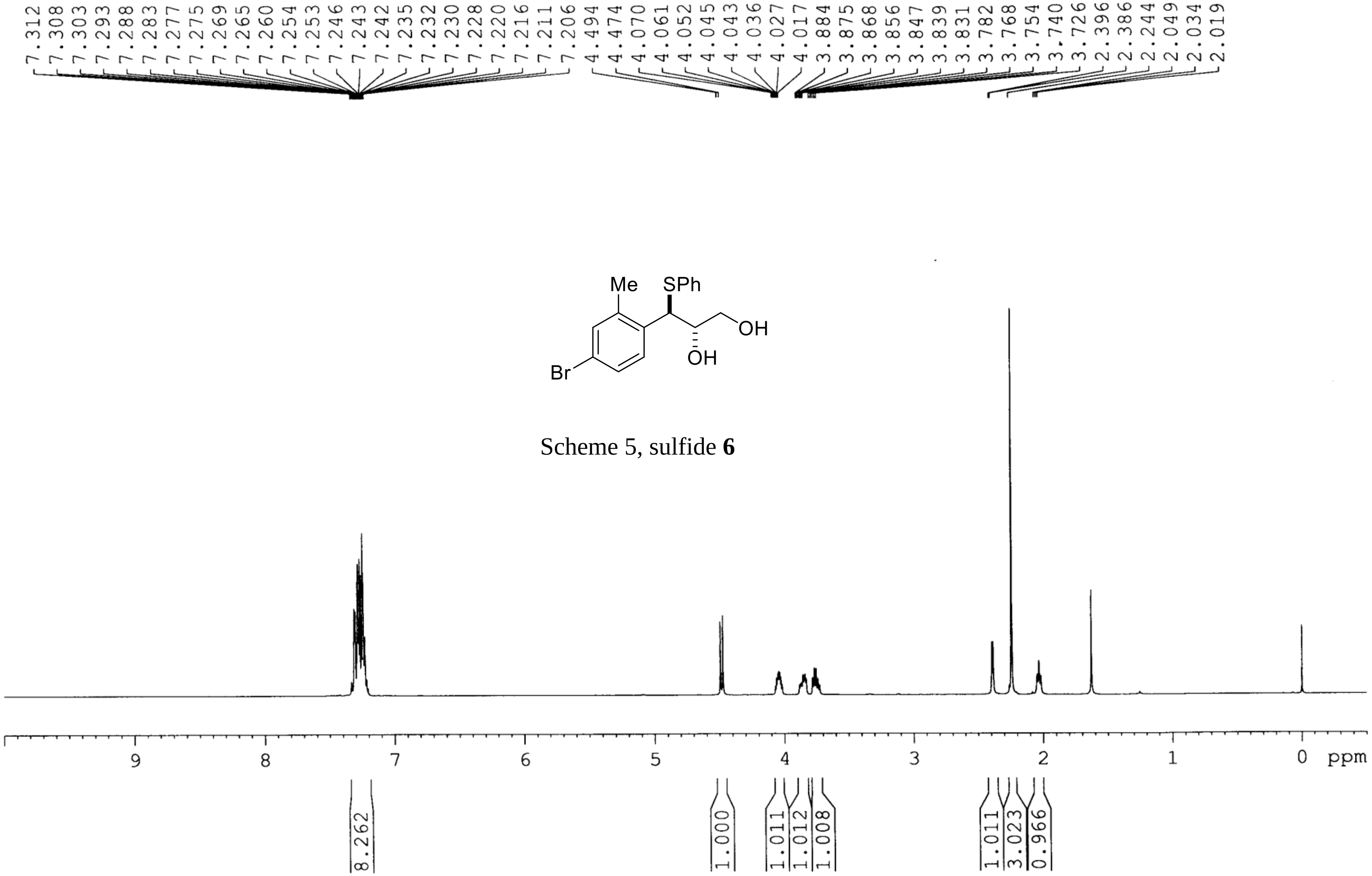

Scheme 5, sulfide **6**

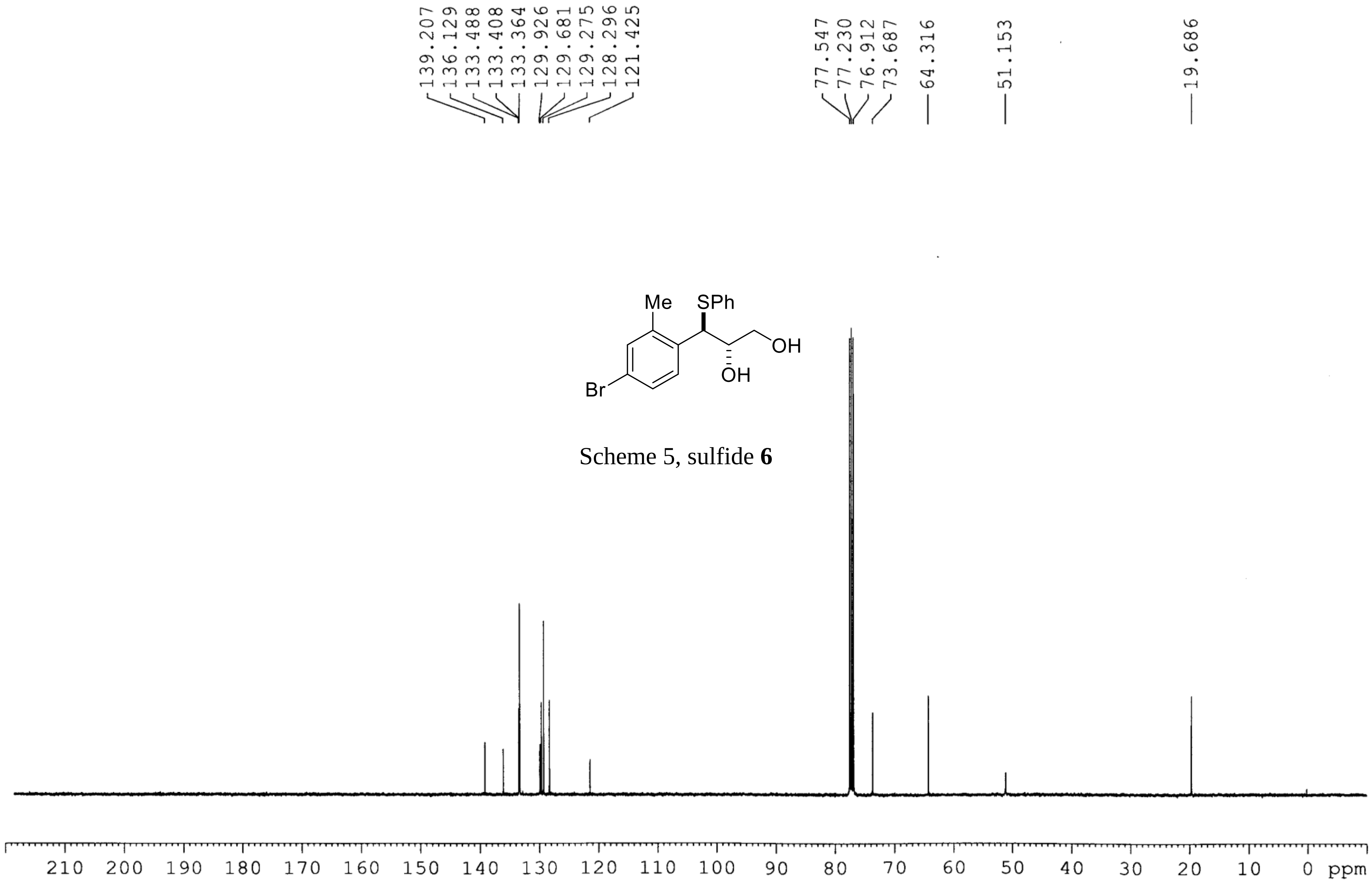

Scheme 5, sulfide **6**

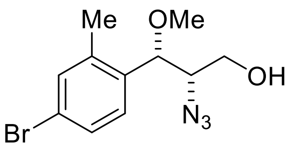

Scheme 6, azide **7**

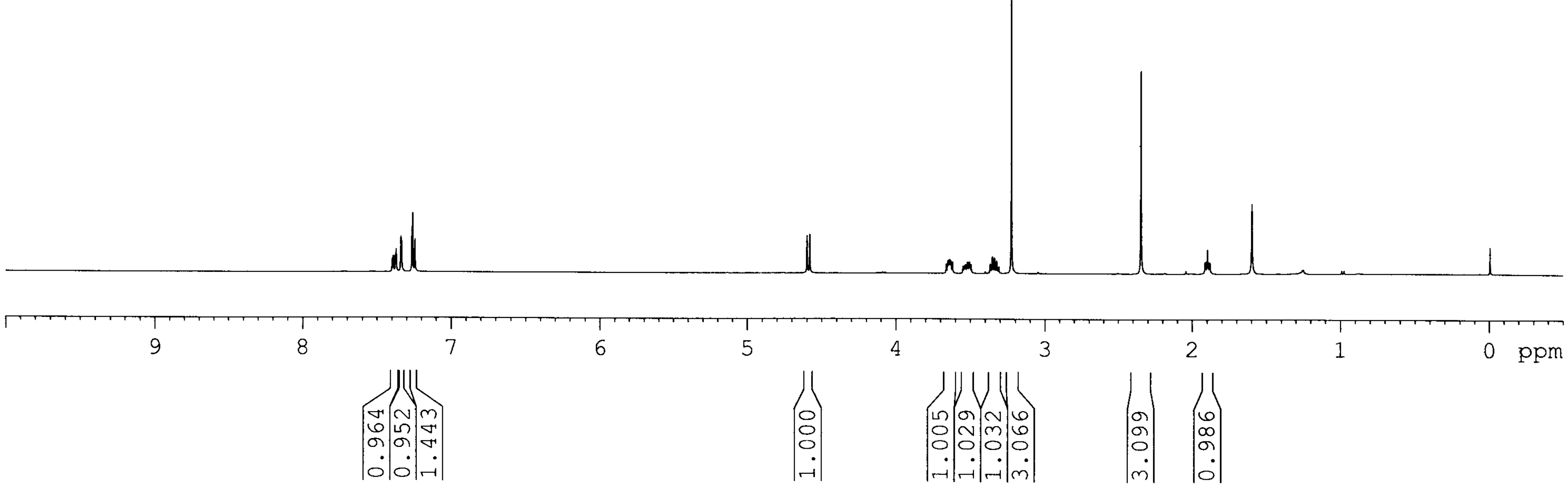

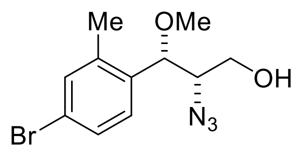

Scheme 6, azide **7**

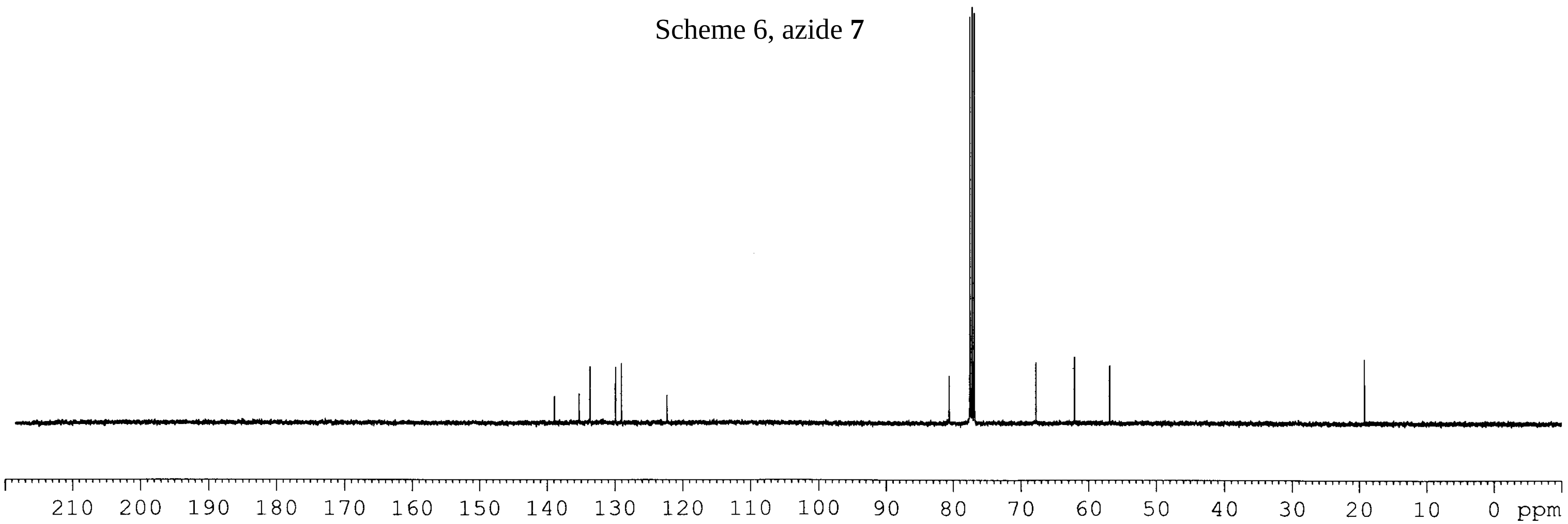

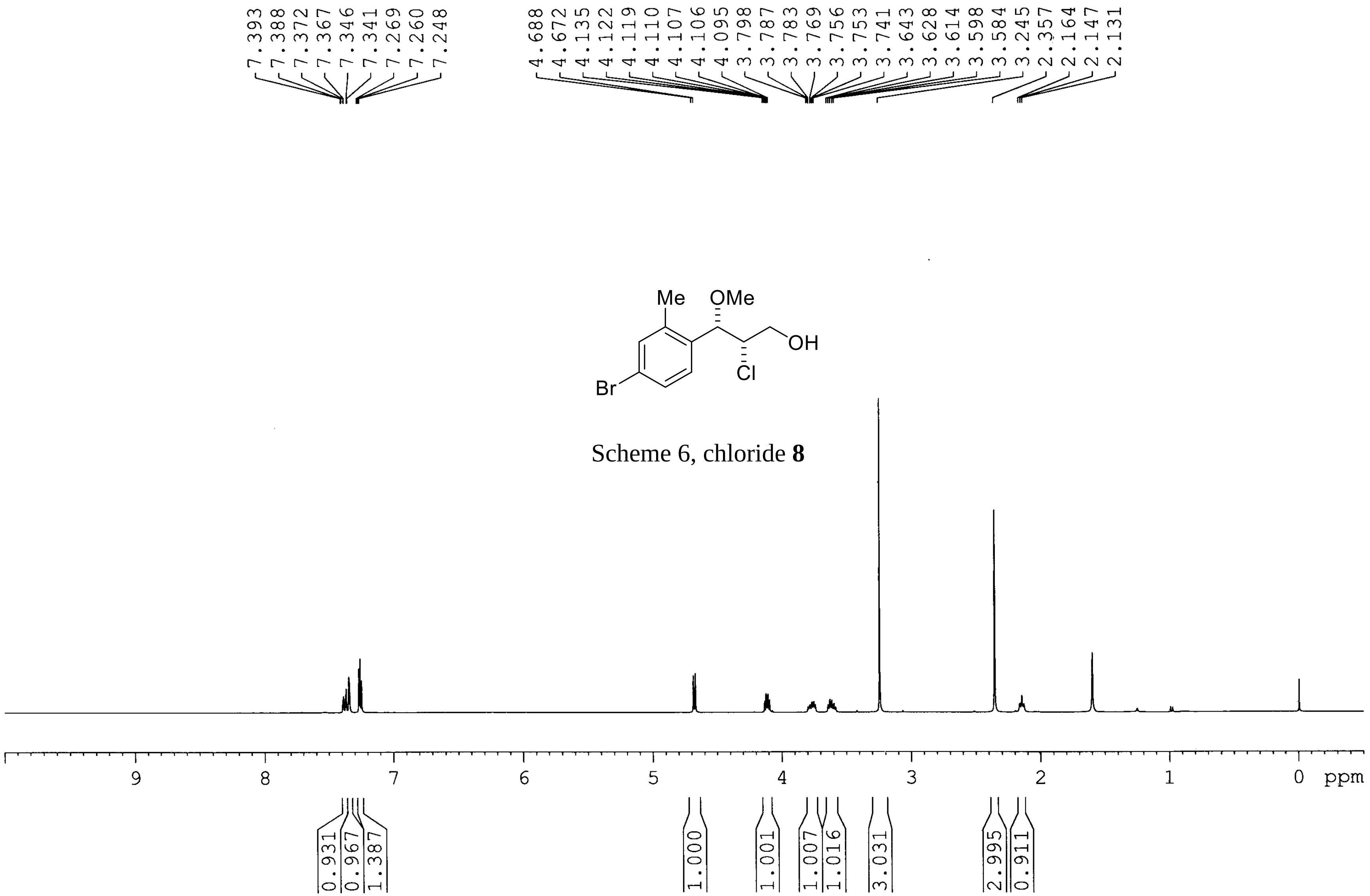

Scheme 6, chloride **8**

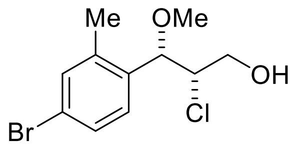

Scheme 6, chloride **8**

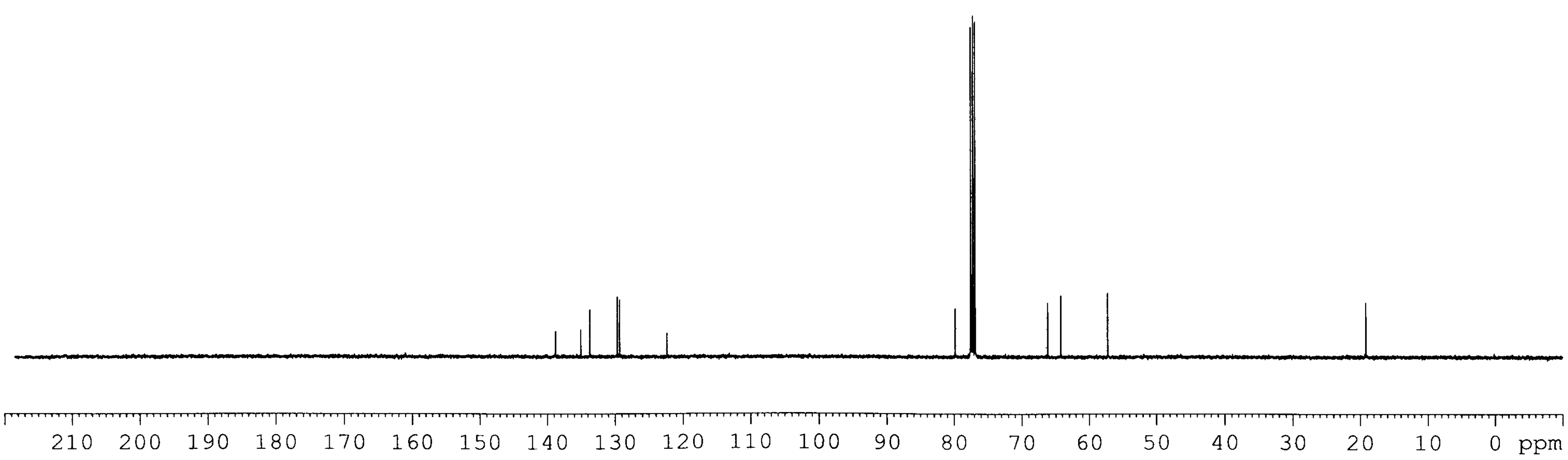

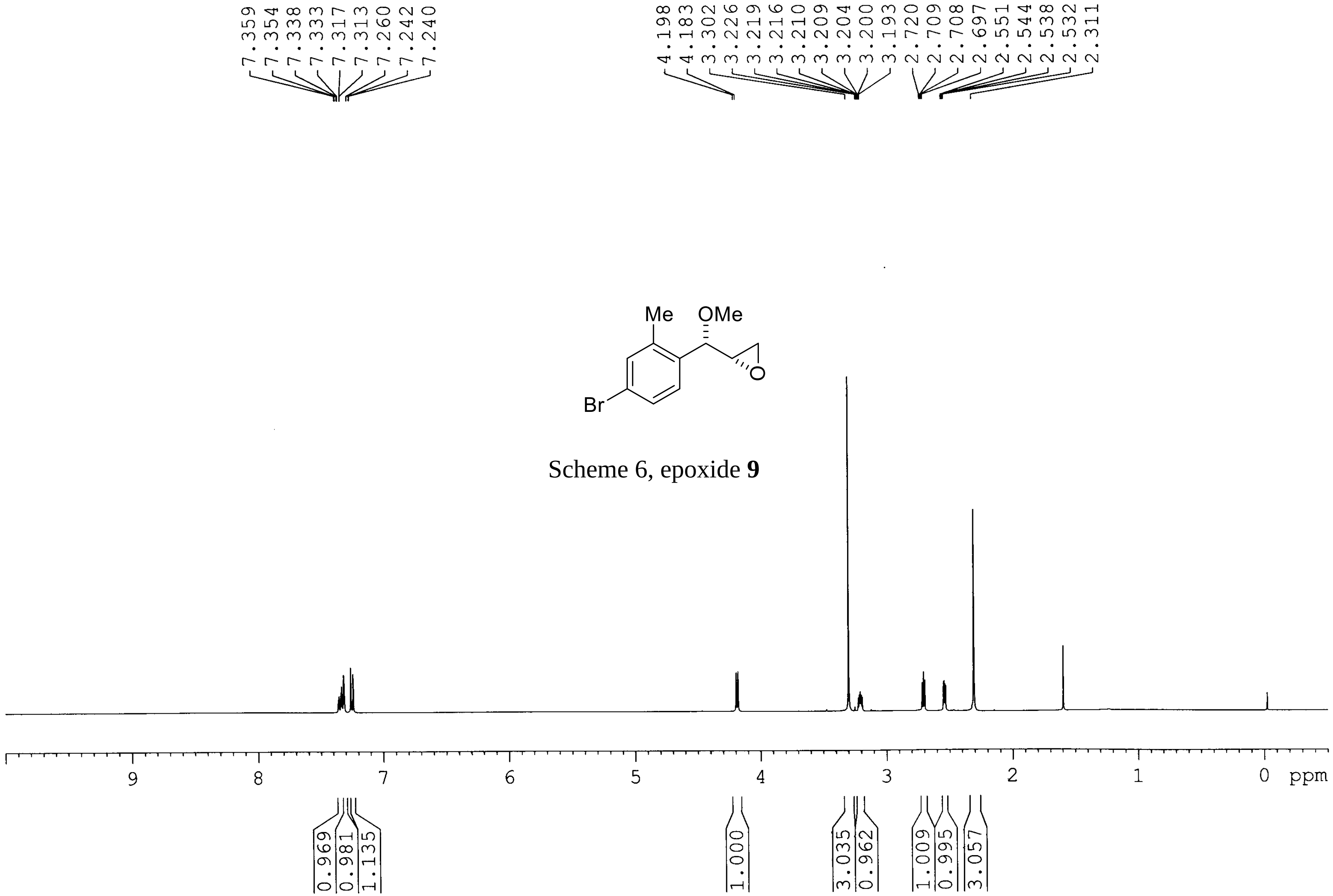

Scheme 6, epoxide **9**

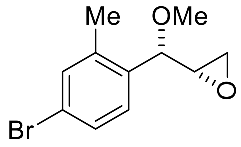

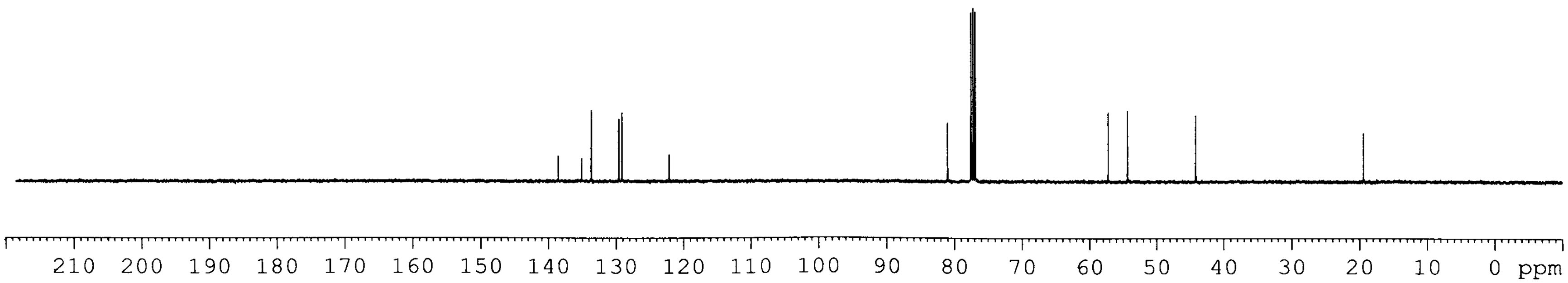

Scheme 6, epoxide **9**

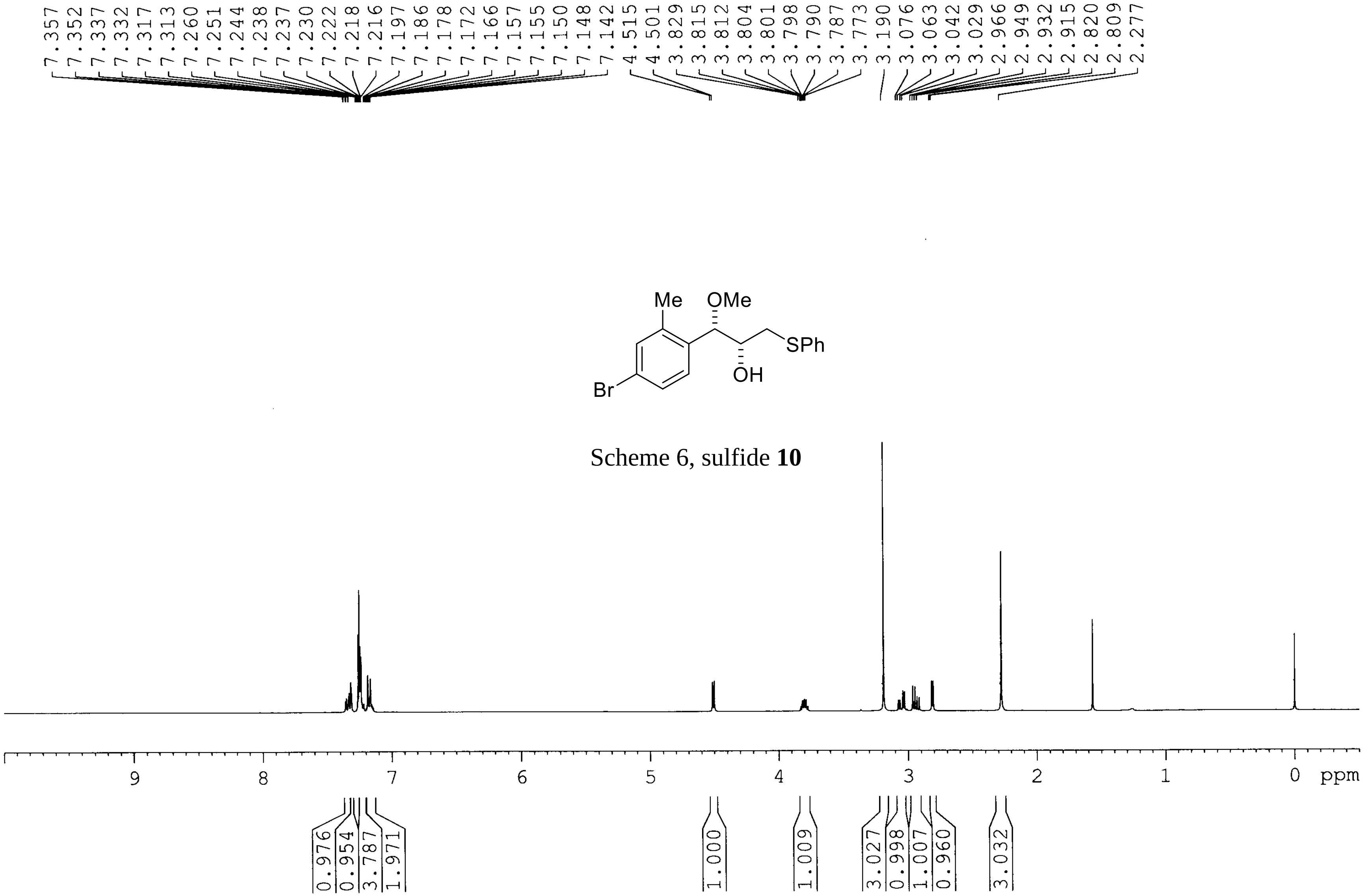

Scheme 6, sulfide **10**

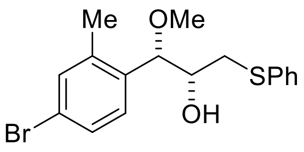

Scheme 6, sulfide **10**

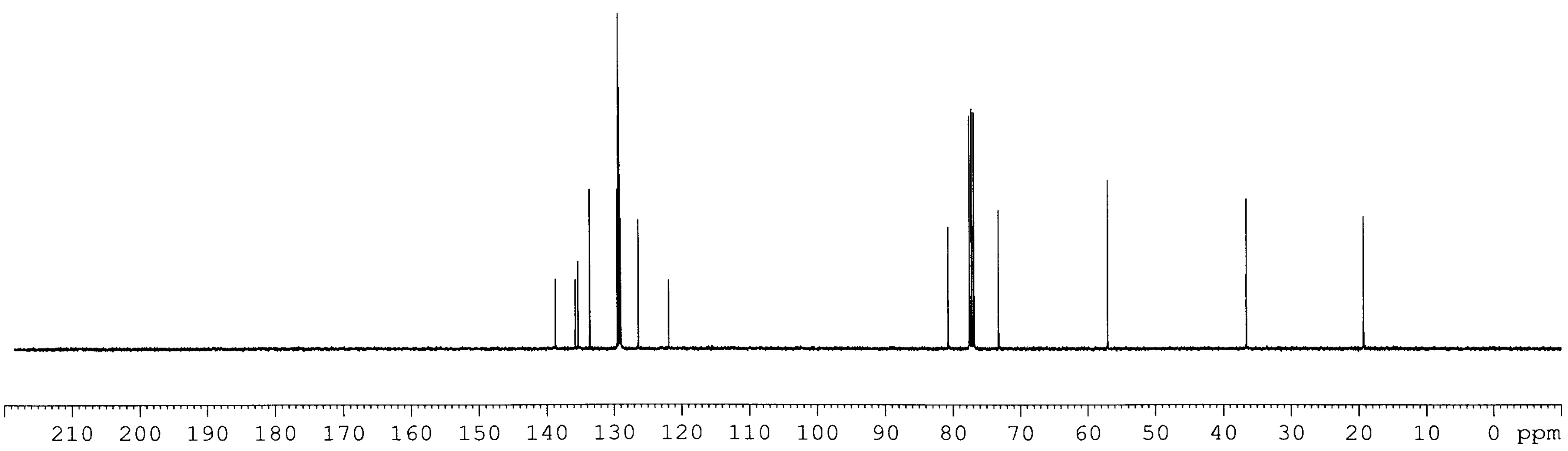

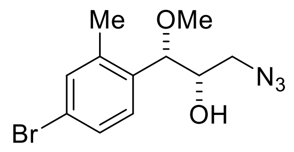

Scheme 7, azide **11**

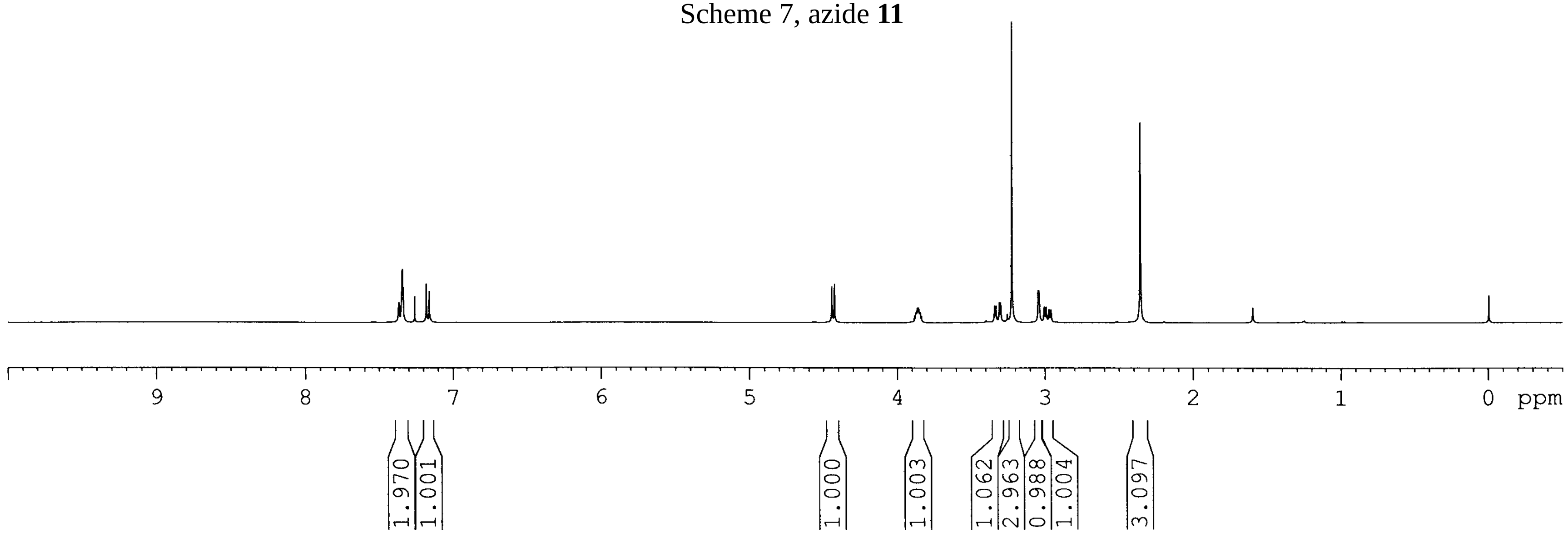

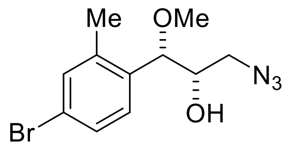

Scheme 7, azide **11**

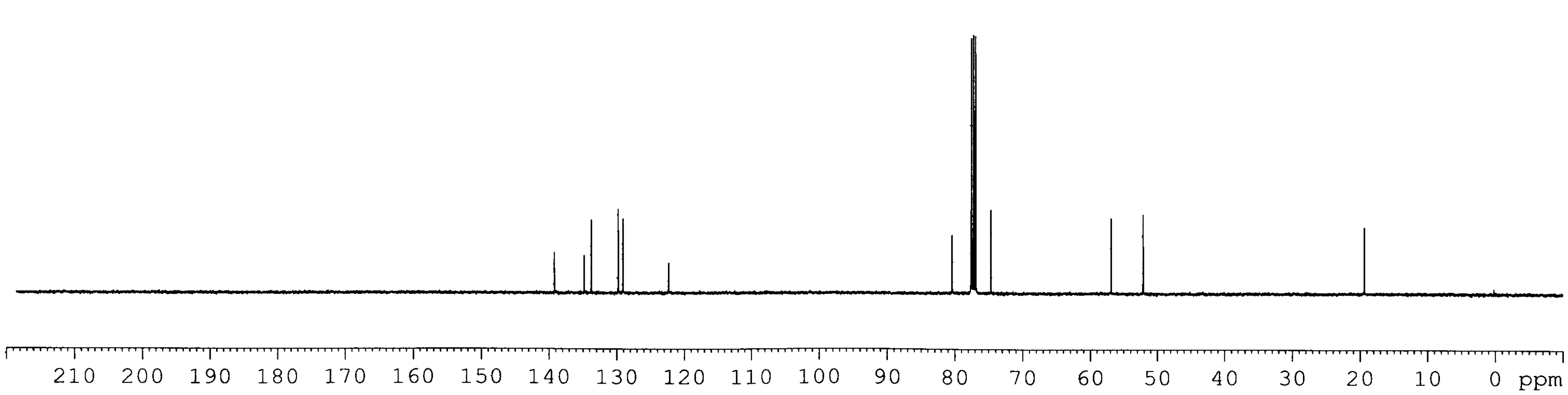

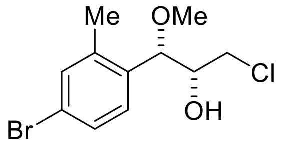

Scheme 7, chloride **12**

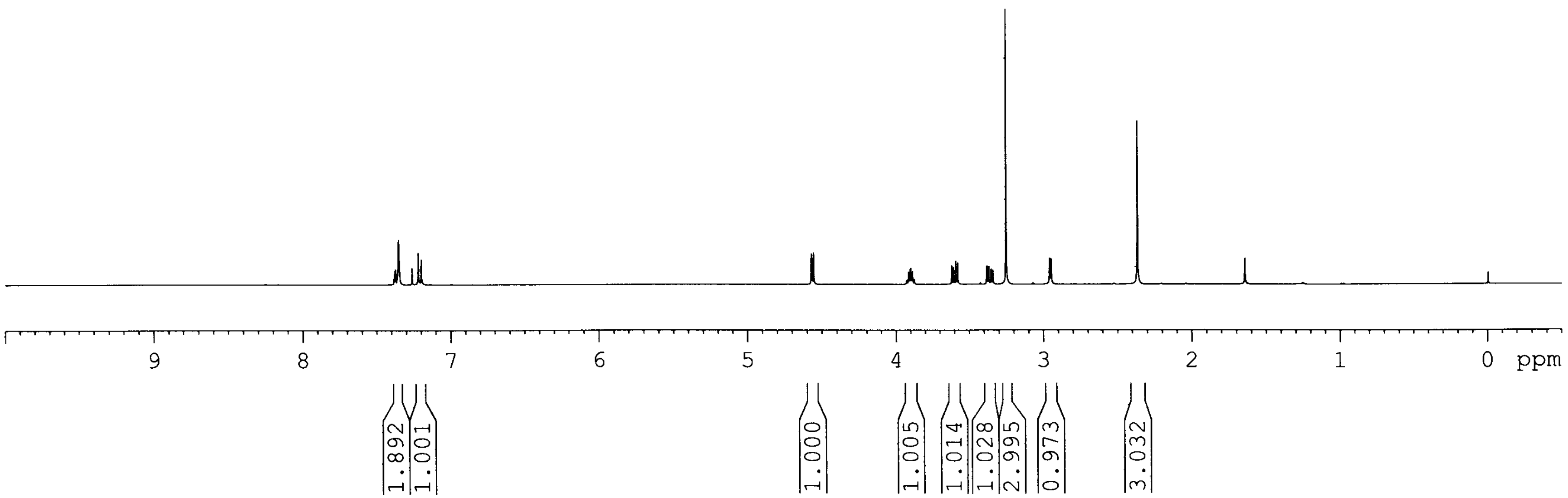

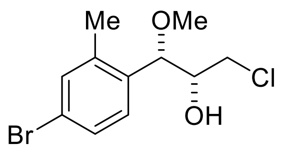

Scheme 7, chloride **12**

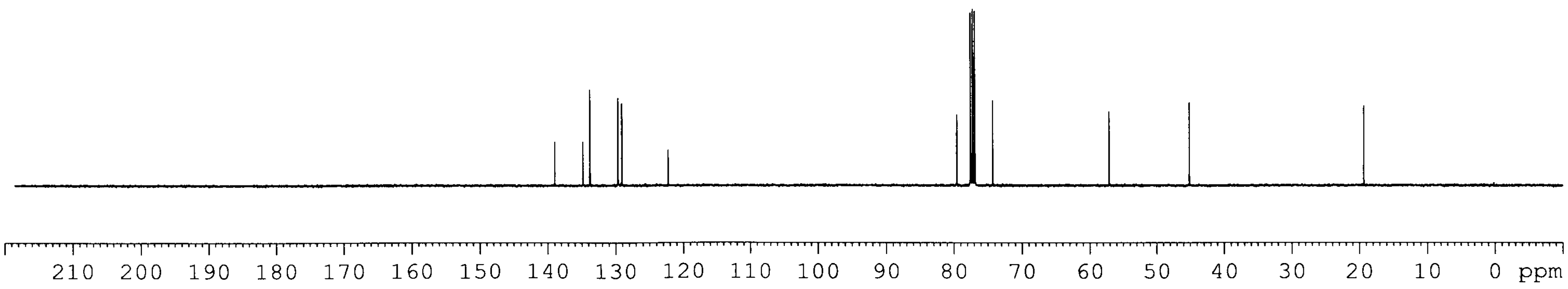

Supplement: RA-011-D1RA02297K-s001 [file RA-011-D1RA02297K-s001.pdf]
